# Nanobody Engineered and Photosensitiser Loaded Bacterial Outer Membrane Vesicles Potentiate Antitumour Immunity and Immunotherapy

**DOI:** 10.1002/jev2.70069

**Published:** 2025-04-16

**Authors:** Peng Xia, Chengming Qu, Xiaolong Xu, Ming Tian, Zhifen Li, Jingbo Ma, Rui Hou, Han Li, Felix Rückert, Tianyu Zhong, Liang Zhao, Yufeng Yuan, Jigang Wang, Zhijie Li

**Affiliations:** ^1^ Zhongnan Hospital of Wuhan University, TaiKang Center for Life and Medical Sciences, Clinical Medicine Research Center for Minimally Invasive Procedure of Hepatobiliary & Pancreatic Diseases of Hubei Province Wuhan University Wuhan Hubei P. R. China; ^2^ Department of Critical Care Medicine, Guangdong Provincial Clinical Research Center for Geriatrics, Shenzhen Clinical Research Centre for Geriatrics, Department of Nuclear Medicine Shenzhen People's Hospital (The First Affiliated Hospital, Southern University of Science and Technology; The Second Clinical Medical College, Jinan University) Shenzhen Guangdong P. R. China; ^3^ Department of Chemistry The University of Chicago Chicago Illinois USA; ^4^ School of Chemistry and Chemical Engineering Shanxi Datong University Datong Shanxi Province P. R. China; ^5^ Harry Perkins Institute of Medical Research, QEII Medical Centre and Centre for Medical Research The University of Western Australia Nedlands WA Australia; ^6^ Department of Visceral Surgery Diakonissen Hospital Speyer Germany; ^7^ Department of Laboratory Medicine Huadong Hospital, Fudan University Shanghai P. R. China; ^8^ Department of Pathology Nanfang Hospital, Southern Medical University Guangzhou P. R. China; ^9^ Department of Pathology & Guangdong Province Key Laboratory of Molecular Tumor Pathology, School of Basic Medical Sciences Southern Medical University Guangzhou P. R. China; ^10^ School of Traditional Chinese Medicine and School of Pharmaceutical Sciences, Guangdong Provincial Key Laboratory of New Drug Screening, School of Pharmaceutical Sciences Southern Medical University Guangzhou Guangdong P. R. China; ^11^ State Key Laboratory for Quality Ensurance and Sustainable Use of Dao‐di Herbs, Artemisinin Research Center, Institute of Chinese Materia Medica China Academy of Chinese Medical Sciences Beijing P. R. China; ^12^ State Key Laboratory of Antiviral Drugs School of Pharmacy Henan University Kaifeng P. R. China

**Keywords:** bacterial outer membrane vesicle, CDH17, nanobody, photoimmunotherapy, STING pathway, tumour‐associated macrophages

## Abstract

Bacterial outer membrane vesicles (OMVs) are promising as antitumour agents, but their clinical application is limited by toxicity concerns and unclear mechanisms. We engineered OMVs with cadherin 17 (CDH17) tumour‐targeting nanobodies, enhancing tumour selectivity and efficacy while reducing adverse effects. These engineered OMVs function as natural stimulator of interferon genes (STING) agonists, activating the cyclic GMP‐AMP synthase (cGAS)‐STING pathway in cancer cells and tumour‐associated macrophages (TAMs). Loading engineered OMVs with photoimmunotherapy photosensitisers further enhanced tumour inhibition and STING activation in TAMs. Combining nanobody‐engineered OMV‐mediated photoimmunotherapy with CD47 blockade effectively suppressed primary and metastatic tumours, establishing sustained antitumour immune memory. This study demonstrates the potential of nanobody‐engineered OMVs as STING agonists and provides insights into novel OMV‐based immunotherapeutic strategies harnessing the innate immune system against cancer. Our findings open new avenues for OMV applications in tumour immunotherapy, offering a promising approach to overcome current limitations in cancer treatment.

## Graphical abstract

Nanobody engineered OMVs loaded with IR700 systemically induce STING signaling in cancer cells and TAMs to enhance antitumor innate immunity and synergistically improve the efficacy of immune checkpoint blockades, potentially triggering sustained antitumor memory.

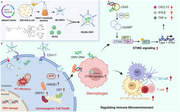

## 1 Introduction

Outer membrane vesicles (OMVs) are nanoscale lipid bilayer structures naturally produced by Gram‐negative bacteria (Xie et al. 2022). These 20–250 nm vesicles inherit surface components and internal cargo from parental bacteria, allowing them to interact with host cells through pathogen‐associated molecular patterns (PAMPs) and pattern‐recognition receptors (PRRs) (Kim et al. 2017; Dhital et al. 2021). OMVs can modulate immune responses and deliver internal cargo into host cells via various mechanisms (Dhital et al. 2021). Recent years have seen growing interest in OMVs for developing novel therapeutic strategies, particularly in cancer treatment. Their intrinsic immunomodulatory properties and nanoscale structures make them attractive candidates for anticancer therapies. Notably, OMVs derived from *Escherichia coli* have shown potential in tumour elimination and induction of long‐term immune responses (Kim et al. 2017).

Despite their promise, two critical issues must be addressed for the successful translation of OMVs into clinical anticancer therapy. First, while interferon‐gamma (IFN‐γ) has been identified as crucial for OMV‐mediated tumour eradication, its activity alone cannot fully explain the potent anticancer performance of OMVs since IFN‐γ can also promote tumour immune evasion and progression through the regulation of immunosuppressive factors (Mojic et al. 2017; Jorgovanovic et al. 2020). Second, safety concerns persist, as OMVs can potentially promote systemic inflammation and tissue damage, even when derived from attenuated or nonpathogenic bacteria (Dhital et al. 2021). To enhance the clinical potential of OMVs, it is essential to improve their tumour selectivity and reduce distribution in normal organs. One approach is to confer active targeting ability to OMVs through genetic modification of parental bacteria, expressing tumour‐targeting peptides or nanobodies on the bacterial membrane surface. This strategy results in engineered OMVs with enhanced tumour selectivity.

Cadherin 17 (CDH17) has emerged as a well‐characterised biomarker for gastrointestinal tumours, including pancreatic ductal adenocarcinoma (Feng et al. 2022), gastric cancers (Ma et al. 2022) and colorectal cancers (Jacobsen et al. 2024). Gastrointestinal tumours account for 26% of the global cancer incidence and 35% of all cancer‐related deaths (Arnold et al. 2020); therefore, therapeutic strategies targeting CDH17 have become a research hotspot for gastrointestinal cancers (Feng et al. 2022; Ma et al. 2022; Xia et al. 2022). We have previously identified several nanobodies that could be used to specifically target CDH17 for gastrointestinal cancer imaging and therapy, suggesting that CDH17 is a promising target for GI cancers and that nanobodies hold great potential for CDH17‐based therapeutics (Xia et al. 2022; Ma et al. 2022).

Innate antitumour immunity, which exerts tumour‐inhibitory effects through the engagement of PRRs with PAMPs, has garnered increasing attention in the field of cancer immunotherapy (Samson and Ablasser 2022; Kwon and Bakhoum 2020). Among the various innate immunity pathways, the cyclic GMP‐AMP synthase‐stimulator of interferon genes (cGAS‐STING) pathway plays a pivotal role in enhancing antitumour immunity. Activation of cGAS‐STING in tumour cells or antigen‐presenting cells (APCs) within the tumour microenvironment (TME) has been shown to promote phagocytosis of tumour antigens, enhance antigen presentation, induce the release of type I interferons (IFNs) and facilitate T cell infiltration, thereby reversing immunosuppressive conditions within the TME (Samson and Ablasser 2022; Low et al. 2024). Given its immunostimulatory potential, a plethora of agonists targeting cGAS‐STING pathway have been preclinically and clinically evaluated across various cancers. Meanwhile, to minimise systemic toxicity while maximising therapeutic efficacy, diverse innovative delivery strategies have been developed to ensure the targeted activation of the STING pathway within the TME. Among these, nanoparticle‐based delivery systems have emerged as a promising approach for encapsulating STING agonists or developing prodrug formulations capable of precise STING activation (Dane et al. 2022; Xian et al. 2024; Chen et al. 2023; Wang et al. 2024), demonstrating significant potential for TME reprogramming and the enhancement of antitumour immunity. Despite these advancements, current STING‐targeting therapies have encountered substantial challenges in clinical translation, often failing to achieve the anticipated therapeutic outcomes. The limited clinical success may be attributed to insufficient tumour‐targeting capability, structural instability of STING agonists, unintended toxicity to T cells and unexpected activation of immunosuppressive B cells, which can dampen antitumour responses (Low et al. 2024; Li et al. 2022). Given these obstacles, refining strategies to achieve localised and selective STING activation presents a compelling direction for the next generation of immunotherapeutic development targeting the STING pathway.

Given the potent antitumour effects of OMVs, this study systematically evaluated the efficacy and mechanistic basis of modified OMVs derived from engineered *E. coli* MG1655 in CDH17‐positive colorectal (CRC) and pancreatic ductal adenocarcinoma (PDAC) models. We demonstrated that CDH17‐targeted OMVs, engineered with nanobody Nb289, significantly enhance therapeutic safety, even at high doses and frequent administration—by selectively sparing normal colon epithelia where CDH17 is confined to basolateral surfaces. Critically, the antitumour activity of Nb289‐OMVs was further amplified by conjugating IRDye700DX (IR700), a clinically approved photoimmunotherapy agent, which enabled synergistic tumour suppression under irradiation. Mechanistically, we reveal that OMVs act as natural STING agonists: their intrinsic bacterial dsDNA cargo directly activates the cGAS‐STING pathway in cancer cells, potentially in tumour‐associated macrophages (TAMs) upon phagocytosis. This triggers immunogenic cell death (ICD) in tumour cells, while IR700 conjugation potentiates ICD via irradiation‐induced ROS, releasing endogenous dsDNA into the TME to broadly activate STING in TAMs. Strikingly, combining IR700@Nb289‐OMVs (single dose) with CD47 nanobodies (two doses) achieved partial tumour eradication and durable immunological memory in subcutaneous and metastatic CRC models, outperforming PD‐1 blockade. This regimen underscores the unique advantage of targeting the CD47‐SIPRα axis to reprogram macrophage‐associate immunosuppression. Collectively, our findings establish nanobody‐engineered OMVs as a translatable STING agonist platform for cancer therapy, particularly when integrated with innate immune checkpoint blockade.

## 2 Materials and Methods

### 2.1 Cell Lines and Cell Culture

Various gastric, colon and pancreatic cancer cell lines were used in this study, obtained from the Cell Bank of Chinese Academy of Sciences (Shanghai, China) and Fuheng Biology (Shanghai, China). The culture conditions were as follows: MKN45 and Colon26 cells were cultured in RPMI1640 (Gibco, USA) supplemented with 10% FBS and 2 mM l‐glutamine. Panc02 cells were maintained in high‐glucose Dulbecco's Modified Eagle's Medium (DMEM) supplemented with 10% FBS and 2 mM l‐glutamine. All cell lines were incubated at 37°C in a humidified atmosphere containing 5% CO_2_.

### 2.2 Preparation of Nanobody‐Engineered OMVs

The plasmid expressing the nanobody fusion protein was transformed into MG1655 bacteria. Subsequently, monoclones were picked in 5 mL LB medium containing 50 µg/mL chloramphenicol and incubated at 37°C at 200 rpm until OD600 ≈ 0.6. Next, the bacteria‐containing medium was cooled on ice for 30 min, and 0.5 mM IPTG was added to induce protein expression. The bacteria were incubated at 200 rpm at 15°C for 16 h. A 1 mL aliquot of bacteria was stained with HA‐Tag mouse mAb (Alexa Fluor®488 Conjugate), and the percentage of positive bacteria was analysed by flow cytometry. The sample was then centrifuged at 10,000 × *g* at 4°C for 10 min using a high‐speed centrifuge, and the supernatant of the nanobody‐engineered bacteria was collected. This supernatant was filtered using a membrane with a pore size of 0.45 µm. Subsequently, an ultracentrifuge process was utilised, with parameters set to 400,000 × *g*, 4°C, for 90 min. The resultant precipitate was resuspended in 1 mL of sterile phosphate buffered saline (PBS). To ensure purity, an endotoxin removal kit was employed to eliminate any endotoxins present. Detailed methods are available in the Supporting Information.

## 3 Results

### 3.1 Preparation and Characterisation of CDH17 Targeting Nanobody Engineered OMVs

**FIGURE 1:**
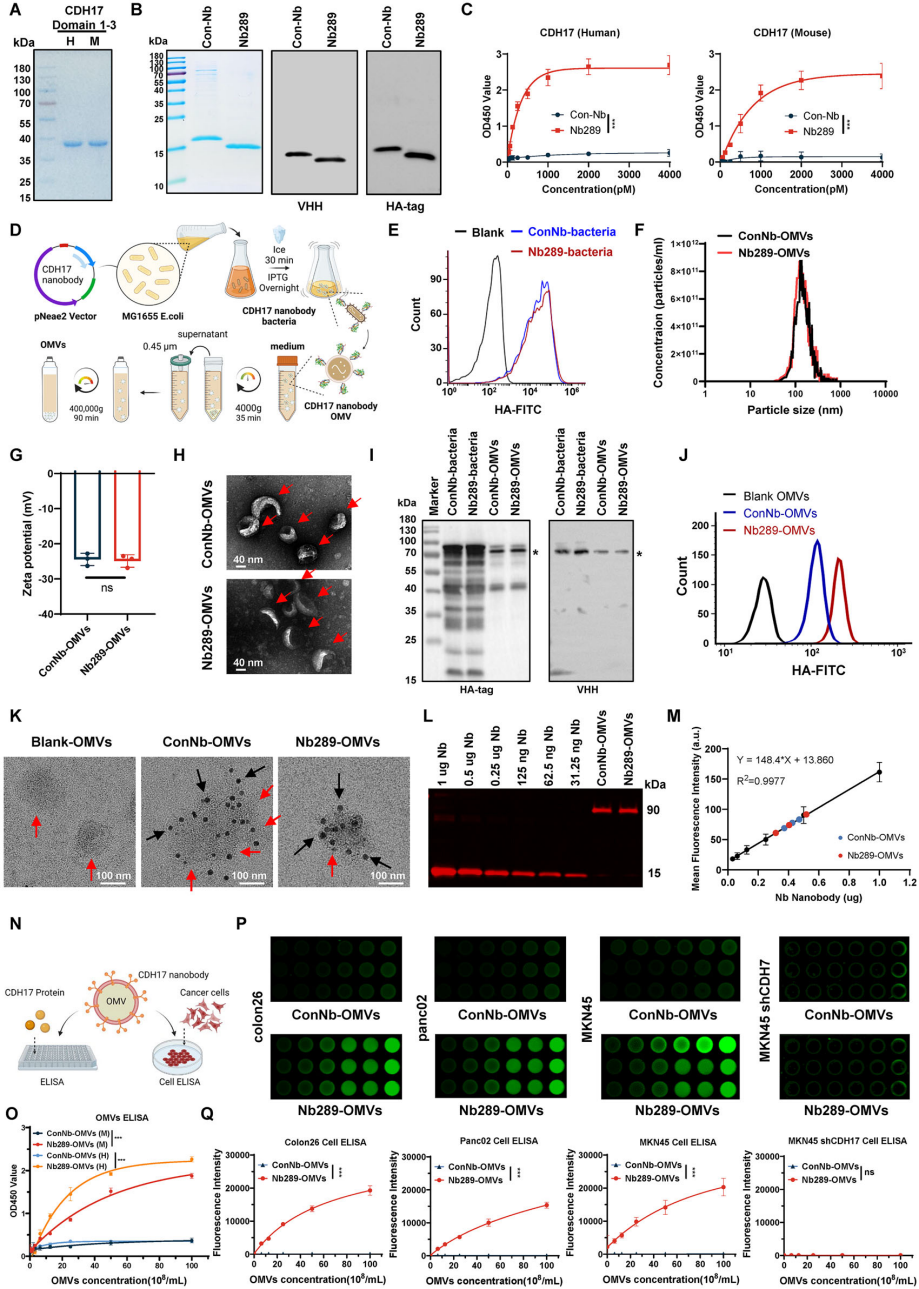
Preparation and characterisation of CDH17 nanobody engineered OMVs. (A) SDS‐PAGE gel analysis of purified human (H) and murine (M) CDH17 Domains 1–3. (B) Purified control (Con‐Nb) and Nb289 were confirmed with VHH and HA tag antibodies by western blot. (C) Binding activity of Nb289 to human and murine CDH17 determined by ELISA (*n* = 3). (D) Nanobody (Nb)‐OMV isolation procedure. (E) Flow cytometry analysis of Nb‐engineered MG1655 using fluorescein isothiocyanate (FITC)‐labelled HA antibody (*n* = 3). (F) Average particle size of Nb‐OMVs measured by nanoparticle tracking analysis (NTA). (G) Zeta potentials of Nb‐OMVs determined by NTA (*n* = 3). (H) Representative images of Nb‐OMVs visualised by transmission electron microscopy (TEM). Scale bars, 40 nm. (I) Detection of Nb‐OMVs by western blot using VHH and HA antibodies. (J) Surface expression of Nbs in engineered OMVs analysed by nanoflow cytometry using an FITC‐labelled HA antibody. (K) Visualisation of engineered OMVs using HA magnetic beads by immunoelectron microscopy. Red and black arrows indicate the OMVs and the HA magnetic beads attached to the OMVs, respectively. Scale bars, 100 nm. (L) Fluorescent western blot analysis of increasing concentrations of Nbs and engineered OMVs using IRDye 680RD‐conjugated anti‐rabbit IgG (*n* = 3). (M) Standard curve established based on the fluorescent intensity–concentration correlation (black dots) from (L) and the determination copy number of displayed Nbs per OMV (blue and red dots). (N) Schematic diagram of ELISA and in‐cell ELISA to measure Nb‐OMVs binding. (O) Binding activity of Nb289‐OMVs to CDH17 determined by ELISA (*n* = 3). (P) Binding activity of Nb289‐OMVs to CDH17 determined by in‐cell ELISA (*n* = 3). (Q) Statistical results of (P). Data are represented as mean ± SD. Statistical significance was calculated using two‐tailed unpaired *t* test analysis (G) or two‐way ANOVA with Tukey's posttest (C, O, Q). ns, no significance. *p < 0.05, **p < 0.01 and ***p < 0.001. ANOVA, analysis of variance; CDH17, cadherin 17; ELISA, enzyme‐linked immunosorbent assay; OMV, outer membrane vesicle; SD, standard deviation.

Nb289 bound to human and murine CDH17 proteins was identified previously through deep sequencing as depicted in the flow chart (Figure S1A) (Xu et al. 2024). In this study, we further confirmed the Nb289 binding capacity to CDH17. The antigens of the human and murine CDH17 Domains 1–3 were purified using a prokaryotic expression system (Figure 1A) and Nb289 and an irrelevant control nanobody (ConNb) were also isolated from the *E. coli* system, which were verified using antibodies against nanobody frames (VHH) and an HA tag that was integrated at their C‐terminal sequences (Figure 1B and Figure S1B). Previously, we demonstrated that the Nb289 bound to human and murine CDH17 with high affinity (*K*_D_ value of 20.1 and 32.5 nM, respectively) (Xu et al. 2024). Now, we confirmed by enzyme‐linked immunosorbent assay (ELISA) that Nb289 could efficiently bind to human and murine CDH17 proteins as compared to the ConNb, which did not indicate any binding ability (Figure 1C).

Our previous data demonstrated that Nb289 could be successfully displayed on the surface of nonpathogenic *E. coli* MG1655 cells through fusion into the pNeae2‐HA vector, as previously described, with the C‐terminal fragment of EHEC intimin (Neae; Residues 1–654) (Xu et al. 2024; Salema et al. 2013). Moreover, the resulting bacteria exhibited an outstanding tumour‐homing ability towards CDH17‐positive cancers. Given that OMVs stem from Gram‐negative bacteria through vesiculation of the outer membrane, we subsequently isolated OMVs by differential centrifugation to investigate whether the nanobodies were properly displayed on the surface of OMVs from Nb‐engineered MG1655 and whether purified OMVs retained their activity against CDH17 proteins (Figure 1D). Flow cytometry analysis showed that ConNb and Nb289 were effectively expressed on the surface of the bacterial membrane (Figure 1E), with the purified ConNb‐OMVs and Nb289‐OMVs having average sizes of 119 ± 25 and 115±31 nm, respectively (Figure 1F) and their corresponding Zeta potentials of −24.4 ± 3.1 and −24.8 ±4.7, respectively (Figure 1G). Morphological analysis by transmission electron microscopy (TEM) revealed heterogeneous round/oval spherical structures with 50–120 nm of diameter (Figure 1H). Western blot and nanoflow cytometry analyses further confirmed that the nanobodies could be detected in the engineered OMVs (Figure 1I) and were localised on the surface of the OMVs (Figure 1J). Immunoelectron microscopy data demonstrated that the nanobody‐engineered OMVs could effectively be labelled and identified using HA magnetic beads, whereas the nonengineered OMVs could not be labelled (Figure 1K). To verify the multivalent surface display of nanobodies on OMVs, we quantified the copy number of nanobodies per OMV. Overall, approximately 70 copies of nanobodies were displayed on the surface of each OMV in both Con Nb‐OMVs and Nb289‐OMVs (Figure 1L, M). In addition, the particle size and concentration of engineered OMVs did not show any detectable changes after 28‐day incubation in PBS solution at 4°C, indicating that the engineered OMVs were highly stable (Figure S2A, B).

Next, to assess whether Nb289‐modified OMVs could specifically recognise CDH17 proteins, we conducted ELISA and in‐cell ELISA (Figure 1N). The data confirmed that Nb289‐OMVs exhibited strong binding capability to human and murine CDH17 proteins, whereas no binding activity was detected for ConNb‐OMVs (Figure 1O). Similarly, in‐cell ELISA data revealed that Nb289‐OMVs could recognise and bind to the natural CDH17 present on the membranes of murine colorectal (Colon26), murine pancreatic (panc02) and human gastric (MKN45) cancer cells. CDH17 knockdown in MKN45 cells almost completely abolished the binding signals of Nb289‐OMVs (Figure 1P, Q), indicating the outstanding specificity of Nb289‐OMVs for CDH17. Collectively, these results demonstrate that nanobody‐engineered OMVs are successfully prepared with the expected nanovesicle structures, and that designed Nb289‐OMVs can specifically recognise CDH17 expressed in human and murine cancer cells.

### 3.2 CDH17 Nanobody‐Engineered OMVs Significantly Improve Safety and Tumour Selectivity

**FIGURE 2:**
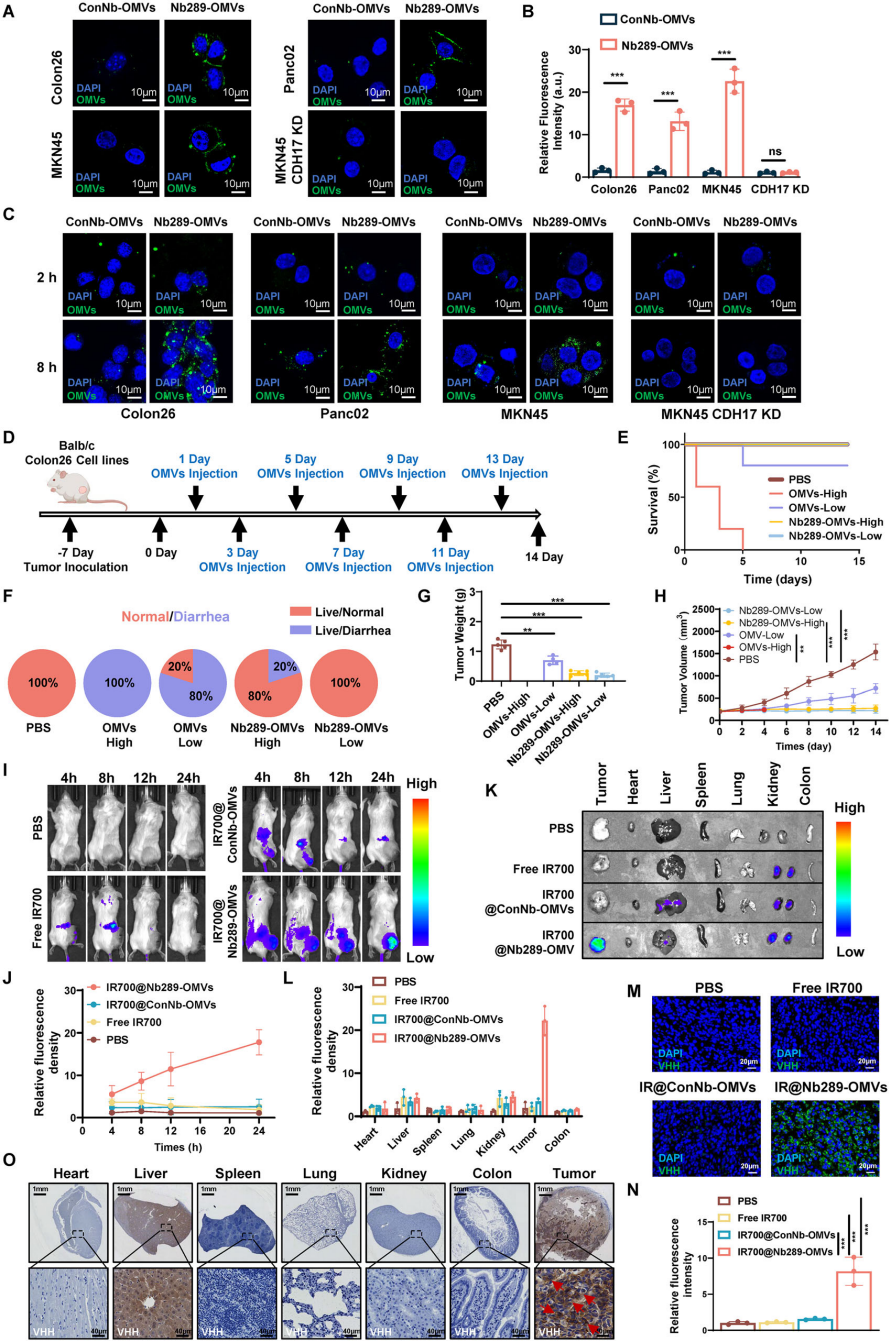
Nanobodies engineered onto OMVs improve the tumour homing ability and safety profile of OMV‐based tumour therapies. (A) Immunofluorescence analysis of CDH17‐associated cancer cells with PKH67‐labelled Nb‐OMVs (green). Scale bars, 10 µm. (B) Quantification of immunofluorescence intensity in (A). (C) Internalisation analysis of Nb‐OMVs in cancer cells overexpressing or lacking CDH17. Scale bars, 25 µm (*n* = 3). (D) OMVs treatment schedule. Colon26 tumour‐bearing mice were treated with PBS (200 µL), OMV‐High (1 × 10^12^ particles/injection), OMV‐Low (1 × 10^11^ particles/injection), Nb289‐OMV‐High (1 × 10^12^ particles/injection) or Nb289‐OMV‐Low (1 × 10^11^ particles/injection) injected via the tail vein at the indicated timepoints. (E) Survival rates of mice treated with different doses of nonengineered or engineered OMVs (*n* = 5). (F) Incidence of diarrhoea in OMV‐treated mice (*n* = 5). (G) Tumour weight at the end of the experiment (*n* = 5). (H) Tumour growth curves of different treatment groups (*n* = 5). (I) In vivo tumour imaging to determine the tumour targeting efficiency of IR700@Nb289‐OMVs (corresponding IR700 dose: 100 µg in each group; *n* = 3). Colon26 tumour‐bearing mice were imaged at four time points (*n* = 3). (J) Quantification of tumour fluorescence intensity in (I). (K) Ex vivo imaging of major organs and tumours collected from mice in (I). (L) Quantification of fluorescence intensity of the various organs in (K) (*n* = 3). (M) Nb‐OMV detection via VHH antibody binding in tumour tissues dissected from mice in (K) (*n* = 3). (N) Quantification of Nb‐OMV fluorescence intensity in (M). (O) Distribution of Nb289‐OMVs in the main organs after 24 h circulation (*n* = 3). The data are presented as mean ± SD. Statistical significance was calculated using two‐tailed unpaired *t* test analysis (B) or one‐way ANOVA with Tukey's post‐test (G, N) or two‐way ANOVA (H, J, L). *p < 0.05, **p < 0.01 and ***p < 0.001. ANOVA, analysis of variance; CDH17, cadherin 17; OMV, outer membrane vesicle; SD, standard deviation.

CDH17 mediates the internalisation of its corresponding nanobodies or nanobody‐modified extracellular vesicles (Xia et al. 2022; Ma et al. 2022). To explore whether Nb289‐OMVs could also be internalised by CDH17‐positive cancer cells, we labelled OMVs with the PKH67 dye and incubated them with immobilised tumour cells. Nb289‐OMVs were found to be significantly enriched in tumour cell membranes with high CDH17 expression compared to ConNb‐OMVs, and this enrichment was almost eliminated upon CDH17 knockdown (Figure 2A, B). In addition, Nb289‐OMVs were efficiently internalised in a time‐ and CDH17 expression‐dependent manner in live tumour cells. Of note, nontargeting ConNb‐OMVs were only slightly internalised, and this process was not affected by CDH17 knockdown (Figure 2C and Figure S3). These results indicate that Nb289 could increase OMVs internalisation via CDH17, potentially implying that Nb289‐OMVs have a high CDH17 targeting ability.

Safety is a crucial issue for OMVs therapeutic application (Xie et al. 2022; Dai et al. 2023). Therefore, we first tested whether the CDH17 nanobody could enhance the safety and enlarge the therapeutic dosing windows for OMVs in vivo. CDH17^+^ Colon26 tumour‐bearing mice were treated every other day with two doses of OMVs (1 × 10^11^ and 1 × 10^12^ particles/injection) (Figure 2D), and the mice were meticulously monitored for potential adverse effects. The animals were euthanised once their body weight was reduced by 20% or any discomfort was identified. All mice injected with a high dose of nonengineered OMVs (1 × 10^12^ particles) exhibited obvious body weight reduction and diarrhoea, and the experiment had to be terminated within 5 days. However, a lower dose of nonengineered OMVs (1 × 10^11^ particles) was relatively well tolerated, and only one mouse was euthanised due to adverse effects, but 80% of the mice (4/5) experienced slight diarrhoea. In contrast, none of the mice treated with Nb289‐OMVs showed significant body weight reduction regardless of the dose administrated, but 20% of mice (1/5) receiving the high dose of Nb289‐OMVs had slight diarrhoea, which proved that the nanobody modification for tumour selectivity ameliorated the systemic side effects of OMVs and enhanced their safety (Figure 2E, F). Concerning their antitumour effect, animal that received nonengineered OMVs (low dose) and engineered Nb289‐OMVs (low and high doses) showed significant tumour regression compared with those treated with PBS (Figure 2G, H). Of note, the engineered Nb289‐OMVs exhibited much better antitumour efficiency than the nonengineered OMVs, but this positive effect remained unaltered regardless of the dose used (Figure 2H and Figure S4A, B). Comprehensive routine blood and biochemical analyses of mice treated with a low dose of Nb289‐OMVs confirmed that various blood parameters, as well as the liver and kidney functions, remained within normal ranges (Figure S4C, D), suggesting a good safety profile. Supported by these data, low dose Nb289‐OMVs (1 × 10^11^ particles) was chosen for subsequent in vivo experiments. Taken together, these results indicate that naturally nonengineered OMVs are toxic and have a very narrow therapeutic dosing window, and the limitations can be overcome via targeting modification. Moreover, a low dose of engineered OMVs showed to be sufficient to hamper tumour growth, which is of particular importance for OMV clinical translation.

Given the relatively high tolerance to a low dose of OMVs (1 × 10¹¹ particles per injection), we next comprehensively compared the safety profiles of unmodified OMVs, ConNb‐OMVs, and Nb289‐OMVs in the Colon26 mouse model at this dosage. Our findings revealed that Nb289‐OMVs exhibited significantly enhanced antitumour effects compared to OMVs and ConNb‐OMVs, the latter two of which demonstrated comparable levels of tumour suppression (Figure S5A–D), consistent with previous findings (Figures 2G, H). Importantly, no mortality due to toxicity was observed in any experimental group at this low dose. However, both unmodified OMVs and ConNb‐OMVs induced similar and significant systemic side effects, including elevated white blood cell counts, liver dysfunction, diarrhoea and weight loss, highlighting their associated toxicity. In contrast, Nb289‐OMVs did not induce such adverse effects (Figure S6A–D). Again, these results underscore that Nb289‐OMVs not only enhance the therapeutic efficacy of OMVs via CDH17 targeting but also significantly improve their safety profile. Untargeted OMVs, including ConNb‐OMVs, exhibit side effects and antitumour efficacy similar to unmodified OMVs. By achieving tumour‐specific targeting, Nb289‐OMVs minimise OMV accumulation in normal tissues, thereby reducing systemic toxicity and demonstrating considerable potential for clinical translation.

Next, we wanted to clearly demonstrate the improved tumour selectivity of Nb289‐OMVs in CDH17^+^ tumour models; to this end, we encapsulated IR700 into the engineered OMVs. IR700 is a new photosensitiser that is used clinically for photoimmunotherapy (PIT) via conjugation with tumour‐targeting antibodies (Nakajima and Ogawa 2024). In addition to PIT upon irradiation with a 685 nm laser, IR700 can also be deployed for tumour imaging in vivo (Inagaki et al. 2021). We attempted to encapsulate IR700 into engineered OMVs to conduct tumour‐homing imaging and simultaneously determine whether engineered OMVs could synergistically suppress tumour progression with IR700‐mediated PIT upon irradiation. Ultraviolet absorption spectrum and high‐performance liquid chromatography (HPLC) analyses confirmed that 500 µg of the highly soluble IR700 dye was effectively loaded into 0.5 × 10^12^ Nb‐OMVs particles (in 1 mL) at a loading efficiency of approximately 67% (Figure S7A–D). The NTA results further uncovered that IR700@Nb289‐OMVs retained the stable biophysical properties, such as concentration and particle size, under mimetic physiological conditions (PBS, 37°C) for 72 h without aggregating (Figure S7E, F). Meanwhile, the particle sizes and number of IR700@Nb‐OMVs did not differed significantly during 2 weeks when stored at 4°C, demonstrating that these nanobody‐engineer OMVs can be preserved for a long time (Figure S7G, H). Additionally, the fluorescence intensity of IR700 in IR700@Nb289‐OMVs remained above 90%, whereas that of free IR700 in aqueous solution decreased to 40% of the initial fluorescence intensity (Figure S7I), indicating that encapsulation by Nb‐OMVs can significantly enhance the stability of IR700. Further analysis of the features of IR700@Nb289‐OMVs was performed in a Colon26 subcutaneous tumour model. When the tumour size reached approximately 200 mm^3^, the mice were randomly treated with PBS, free IR700, or nontargeting or CDH17‐targeting Nb‐OMVs loaded with IR700 (identified as IR700@ConNb‐OMVs and IR700@Nb289‐OMVs, respectively). The OMVs were injected intravenously, and their biological distribution was observed at different time points after administration (4, 8, 12 and 24 h). Compared with free IR700 and IR700@ConNb‐OMVs, the fluorescence signals in tumours treated with IR700@Nb289‐OMVs were much stronger and gradually increased over time (Figure 2I, J). The fluorescence signals mainly accumulated at the tumour sites 24 h after injection with IR700@Nb289‐OMVs, whereas the signals were almost undetectable in the tumours of mice treated with free IR700 or IR700@ConNb‐OMVs (Figure 2I, J). These results indicate that IR700@Nb289‐OMVs have excellent specificity and homing ability for CDH17^+^ colorectal cancer. Ex vivo imaging of the main organs collected 24 h after injection revealed that the strongest signals were identified in tumour tissues from mice treated with IR700@Nb289‐OMVs (Figure 2K, L). Further results from VHH antibody staining showed that the nanobodies could be detected in IR700@Nb289‐OMVs‐treated tumour tissues, but not tissues from mice treated with IR700@ConNb‐OMVs (Figure 2M, N). Despite of the good selectivity of IR700@Nb289‐OMVs for the tumour tissues, some nanobodies were also detected in the liver and kidney of these mice, possibly owing to nonspecific OMVs uptake (Figure 2O).

Given the high expression of CDH17 in normal intestinal epithelial cells, previous studies have indicated that its localisation is restricted to the lateral membranes rather than the apical or basal surfaces, thereby limiting the accessibility of CDH17‐targeting agents to normal intestinal cells (Feng et al. 2022). In contrast, CDH17 is prominently expressed around tumour cells, making it accessible for CDH17‐targeting drugs. Our immunofluorescence results further confirmed that CDH17 was predominantly localised to the lateral membranes of colonic epithelial cells, while in subcutaneous tumours derived from Colon26 cells, CDH17 was prominently expressed around tumour cells (Figure S8A, B). This pericellular distribution in tumour cells enables more effective OMV accumulation within the tumour mass. Consequently, this unique localisation pattern restricts OMV access to normal colonic tissues, minimising interactions with CDH17 in healthy cells, consistent with prior studies (Feng et al. 2022).

To further confirm that CDH17 nanobody‐engineered OMVs enhance tumour selectivity and minimise off‐target accumulation, we employed the more sensitive near‐infrared (NIR)‐II imaging system using ICG‐conjugated OMVs. In vivo imaging demonstrated that Nb289‐OMVs exhibited significantly higher tumour accumulation at 24 h postinjection compared to ConNb‐OMVs (Figure S9A, C), highlighting their targeted delivery advantage. Ex vivo organ imaging further revealed that Nb289‐OMVs were primarily retained in tumours, with minimal distribution in normal tissues except for slight hepatic accumulation. In contrast, ICG signals in ConNb‐OMVs were predominantly localised in liver tissues, with minor presence in lungs (Figure S9B, D). Furthermore, IHC analysis confirmed that Nb289‐OMVs preferentially accumulated in tumours and liver tissues involved in OMV metabolism, whereas ConNb‐OMVs were mainly detected in normal liver tissues but not tumours (Figure S9E). These results collectively demonstrate that Nb289 modification enhances tumour‐specific OMV accumulation, improves drug delivery efficiency and reduces systemic toxicity.

Taken together, all the in vitro and in vivo results with dual‐imaging approach using IR700 and ICG provide compelling evidence that Nb289 modification significantly enhances tumour selectivity while reducing nonspecific accumulation in normal organs. This not only improves the safety profile of OMVs but also enhances the delivery efficiency of imaging probes and therapeutic agents such as IR700.

### 3.3 Nanobody‐Engineered OMVs‐Mediated Photoimmunotherapy Effectively Inhibits Tumour Growth

**FIGURE 3:**
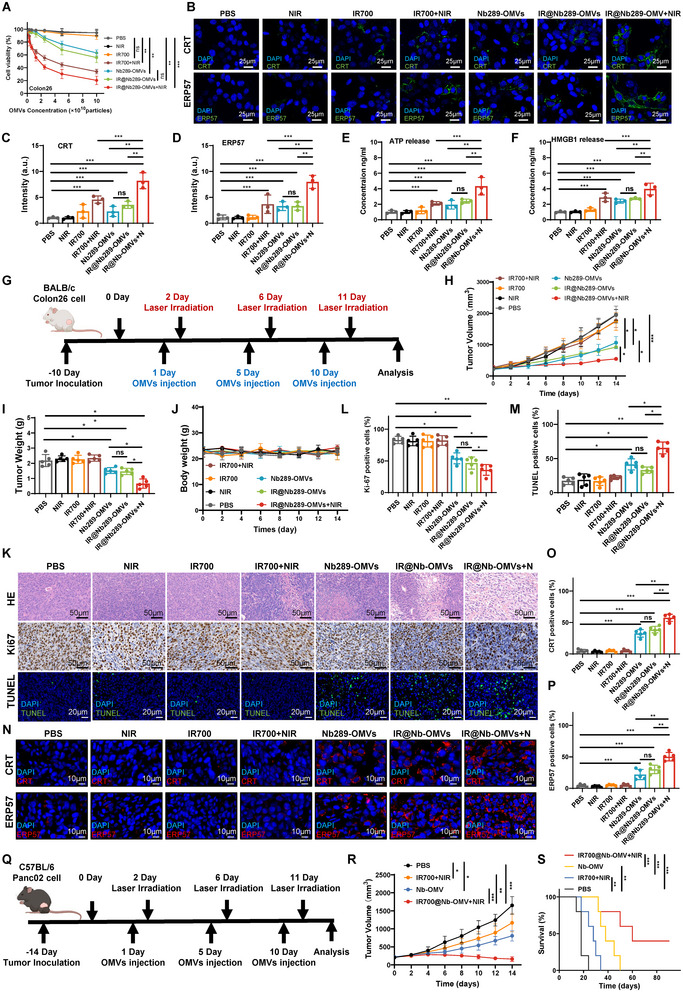
IR700‐loaded nanobody‐engineered OMVs effectively inhibit the growth of colorectal and pancreatic tumours in mice. (A) Viability of Colon26 cells measured by CCK‐8 assay upon treatment with PBS (200 µL), NIR (20 J/cm^2^), IR700 (10 µg/mL), IR700 (10 µg/mL) + NIR (20 J/cm^2^), Nb289‐OMVs (1 × 10^10^ particles/mL), IR700@Nb289‐OMVs (1 × 10^10^ particles/mL) and IR700@Nb289‐OMVs (1 × 10^10^ particles/mL) + NIR (20 J/cm^2^) (*n* = 3 per treatment). (B) Immunofluorescent analysis of translocated CRT and ERp57 in Colon26 cancer cells upon the same treatments as in (A). (C) Quantification of fluorescence intensity of CRT surface expression in (B). (D) Quantification of fluorescence intensity of ERp57 translocation in (B). (E, F) Quantification of ATP (E) and HMGB1 (F) released from Colon26 cancer cells after the indicated treatments (*n* = 3). (G) Treatment schedule of Colon26 tumour‐bearing mice with PBS (200 µL), NIR (50 J/cm^2^), IR700 (100 µg), IR700 (100 µg) + NIR (50 J/cm^2^), Nb289‐OMVs (1 × 10^11^ particles/injection), IR700@Nb289‐OMVs (1 × 10^11^ particles/injection) or IR700@Nb289‐OMVs (1 × 10^11^ particles/injection) + NIR (50 J/cm^2^). (H) Growth curves of Colon26 tumours upon treatment (*n* = 5). (I) Tumour weight at the end of the experiment upon different treatments (*n* = 5). (J) Body weight changes of mice during the treatment period (*n* = 5). (K) H&E, Ki67 and TUNEL staining of tumours from the different treatment groups (*n* = 5). (L) Quantification of Ki67‐positive cells in tumour samples. (M) Quantification of TUNEL staining in (L). (N) Immunofluorescent staining for the detection of CRT and ERp57 in cell membranes (*n* = 5). (O, P) Quantification of surface‐expressed CRT (O) and ERp57 (P) in (N). (Q) Treatment schedule of Panc02 tumour‐bearing mice with PBS (200 µL), IR700 (100 µg) + NIR (50 J/cm^2^), Nb289‐OMVs (1 × 10^11^ particles/injection) or IR700@Nb289‐OMVs (1 × 10^11^ particles/injection) + NIR (50 J/cm^2^). (R) Growth curves of Panc02 tumours upon treatment (*n* = 5). (S) Survival curves of Panc02 tumour‐bearing mice in different treatment groups (*n* = 5). The data are presented as mean ± SD. Statistical significance was calculated using two‐way ANOVA (A, H, J, R) or one‐way ANOVA with Tukey's post‐test (C–F, I, L, M, O, P) or Mantel–Cox test (S). *p < 0.05, **p < 0.01 and ***p < 0.001. ANOVA, analysis of variance; CRT, calreticulin; ERp57, endoplasmic reticulum protein 57; H&E, haematoxylin and eosin; NIR, near‐infrared; OMV, outer membrane vesicle; PBS, phosphate buffered saline; SD, standard deviation.

Having shown the excellent tumour‐homing ability of IR700@Nb289‐OMVs, we further investigated the antitumour effect of IR700@Nb289‐OMVs in vitro *and* in vivo. Firstly, we compared the cell‐killing ability of OMVs, ConNb‐OMVs and Nb289‐OMVs in CDH17^+^ Colon26 and MKN45 cell lines, as well as in CDH17 knockdown MKN45 cells. Nb289‐OMVs exhibited greater cell‐killing effects in Colon26 and MKN45 cells compared to OMVs and ConNb‐OMVs, which showed no notable difference from each other. In CDH17‐knockdown MKN45 cells, the cell‐killing ability of all three groups was comparable (Figure S10A, B). This is consistent with previous reports showing that standalone OMVs can induce cell death (Dhital et al. 2021; Jin et al. 2022). Moreover, Nb289‐OMVs demonstrated greater inhibitory activity against cancer cells through enhanced internalisation. Further, cell viability analysis of Colon26 cancer cells indicated that IR700 or its corresponding NIR laser irradiation (685 nm) alone had no effect on cell viability; however, free IR700 activated by NIR irradiation could significantly inhibit cell growth when compared with PBS (Figure 3A). As anticipated, IR700@Nb289‐OMVs plus irradiation led to the maximal cell growth inhibition among all treatments in a dose‐dependent manner (Figure 3A). Finally, we compared the therapeutic effects of IR700@Nb289‐OMVs and IR700@ConNb‐OMVs. In Colon26 and MKN45 cells, IR700@Nb289‐OMVs exhibited significantly enhanced cell‐killing ability compared to IR700@ConNb‐OMVs. However, in CDH17‐knockdown MKN45 cells, there was no significant difference between the two groups (Figure S10C, D). These results further validate the critical role of Nb289 targeting in improving therapeutic outcomes. This in vitro result was further confirmed by consistent clone formation assay, live/dead cell staining and Annexin V/PI flow cytometry data, showing that IR700@Nb289‐OMVs plus irradiation resulted in the most significant cell death and cell apoptosis among all groups, while free IR700 plus irradiation, Nb289‐OMVs and IR700@Nb289‐OMVs without light exposure also exhibited cell growth inhibition and apoptosis to a certain extent (Figure S11A–F). Scratch‐wound healing and Transwell invasion assays also showed that IR700@Nb289‐OMV with NIR irradiation efficiently reduced the migration and invasion ability of colon cancer cells (Figure S12A–D). Together, these results suggest that IR700 encapsulation in Nb289‐OMVs can improve the inhibitory efficacy of OMVs concerning the viability, migration and invasion of CDH17^+^ cancer cells, and ultimately induce more cancer cell death.

IR700, as a photoimmunotherapy agent, can elicit reactive oxygen species (ROS) production and induce ICD of cancer cells upon internalisation and light irradiation (Vranes et al. 2019; Mao et al. 2022). ICD is characterised by the translocation of calreticulin (CRT) and endoplasmic reticulum protein 57 (ERp57) from the endoplasmic reticulum to the cell surface, release of ATP, and increased secretion of high‐mobility group B1 (HMGB1) from the nucleus (Kroemer et al. 2013). Therefore, we analysed the ROS production and ICD induction in Colon26 cancer cells treated with IR700‐loaded Nb289‐OMVs. Treatment with IR700@Nb289‐OMVs or free IR700 produced ROS in the singlet oxygen form after irradiation, whereas NIR, Nb289‐OMVs or IR700@Nb289‐OMVs alone were incapable of generating ROS (Figure S13A–D). ICD induction was detected in colon cancer cells after various treatments. As expected, both free IR700 and IR700@Nb289‐OMVs plus NIR induced CRT and ERp57 surface exposure (Figure 3B–D), ATP release, and HMGB1 secretion in treated colon cancer cells, which aligns with the ROS production (Figure 3E, F). Notably, IR700@Nb289‐OMVs plus NIR light exhibited the strongest ICD effect. Intriguingly, we found that Nb289‐OMVs and IR700@Nb289‐OMVs alone could also increase CRT and ERp57 translocation, as well as release of ATP and HMGB1, clearly suggesting ICD induction. Although it has been reported that OMVs can lead to apoptosis, pyroptosis and autophagy (Dhital et al. 2021), no studies had previously suggested that OMVs could directly induce ICD.

We then established a subcutaneous transplantation tumour model using syngeneic Colon26 cancer cells in BALB/c mice and conducted in vivo antitumour treatment as the indicated schedule (Figure 3G). We observed that NIR alone, free IR700 and free IR700 plus NIR had almost no effect on tumour growth, whereas Nb289‐OMVs and IR700@Nb289‐OMVs showed similar tumour inhibitory effects after three administrations. However, IR700@Nb289‐OMVs plus NIR irradiation almost prevented tumour progression and showed the most significant tumour suppression among all treatments after three treatment cycles (Figure 3H, I and Figure S14A). Hence photoimmunotherapy mediated by IR700@Nb289‐OMVs could synergistically enhance the antitumour activity of Nb289‐OMVs, and free IR700 without tumour‐targeted delivery is ineffective on tumour growth. During the entire treatment period, the mice showed no obvious signs of toxicity, which was confirmed by no obvious major organs tissue damage detected by haematoxylin and eosin (H&E) staining at the end of the experiment (Figure S14B). Furthermore, routine blood and blood biochemical analyses for the systematic toxicity confirmed that all parameters were within normal ranges after treatment (Figure S15A, B). These results demonstrate the good biocompatibility and biosafety of engineered OMV‐mediated photoimmunotherapy. In contrast, H&E, Ki67 and TUNEL staining of tumour tissues unravelled that IR700@Nb289‐OMVs plus NIR treatment significantly inhibited cancer cell proliferation, and induced cell necrosis and apoptosis compared with the other treatments, including Nb289‐OMVs and IR700@Nb289‐OMVs alone (Figure 3K–M), both of which similarly suppressed Ki67 expression and promoted apoptosis, in line with the data from tumour growth curves and tumour weight (Figure 3H, I). Moreover, CRT and ERp57 translocation was significantly increased after treatment with IR700@Nb289‐OMVs plus NIR, while Nb289‐OMVs and IR700@Nb289‐OMVs treatment only slightly enhanced the translocation (Figure 3N–P), all of which aligned with the data for in vitro ICD detection (Figure 3B–F). To further elucidate the critical role of Nb289 in therapy, we systematically compared the therapeutic efficacy of various IR700‐loaded OMVs, including Nb289‐OMVs, ConNb‐OMVs and unmodified OMVs, in Colon26 tumour‐bearing mice. The results revealed that both IR700@ConNb‐OMVs plus NIR and IR700@OMVs plus NIR exhibited similar, moderate tumour suppression. However, IR700@Nb289‐OMVs plus NIR demonstrated significantly superior antitumour efficacy compared to all groups (Figure S16A–C). These findings indicate that IR700‐loaded untargeted nanobody‐engineered OMVs plus irradiation induce tumour suppression comparable to IR700@unmodified OMVs plus irradiation; they do not confer additional therapeutic benefits beyond unmodified IR700@OMVs. In contrast, targeted IR700@Nb289‐OMVs plus irradiation exhibit markedly enhanced tumour suppression, further supporting the tumour‐targeting capability and drug delivery efficiency of Nb289‐OMVs in vivo. Additionally, to extend our findings, we explored whether IR700@Nb289‐OMVs plus NIR could effectively regress tumour progression in another CDH17‐positive tumour model induced by the pancreatic cancer cell line Panc02 (Figure 3Q). We observed that IR700@Nb289‐OMVs plus NIR promoted sustained tumour inhibition and significantly prolonged survival even in this refractory desmoplastic tumour model. Three injections of Nb289‐OMVs alone partially inhibited tumour growth and extended survival to some extent (Figure 3Q–S).

These results clearly demonstrate that IR700@Nb289‐OMVs plus NIR can promote maximal tumour suppression efficacy by combining the IR700‐mediated photoimmunotherapeutic effect with the intrinsic tumour inhibitory capability of OMVs. Of note, IR700@Nb289‐OMVs plus NIR can efficiently promote ICD occurrence in tumour tissues, but engineered OMVs alone appear to also be able to induce ICD in vitro and in vivo as well. Interestingly, ICD occurrence implies that Nb289‐OMVs and IR700@Nb289‐OMVs can enable the TME to be potentially reprogramed.

### 3.4 Nanobody‐Engineered OMVs Alone or Coupled With Photoimmunotherapy Can Reshape the TME

**FIGURE 4:**
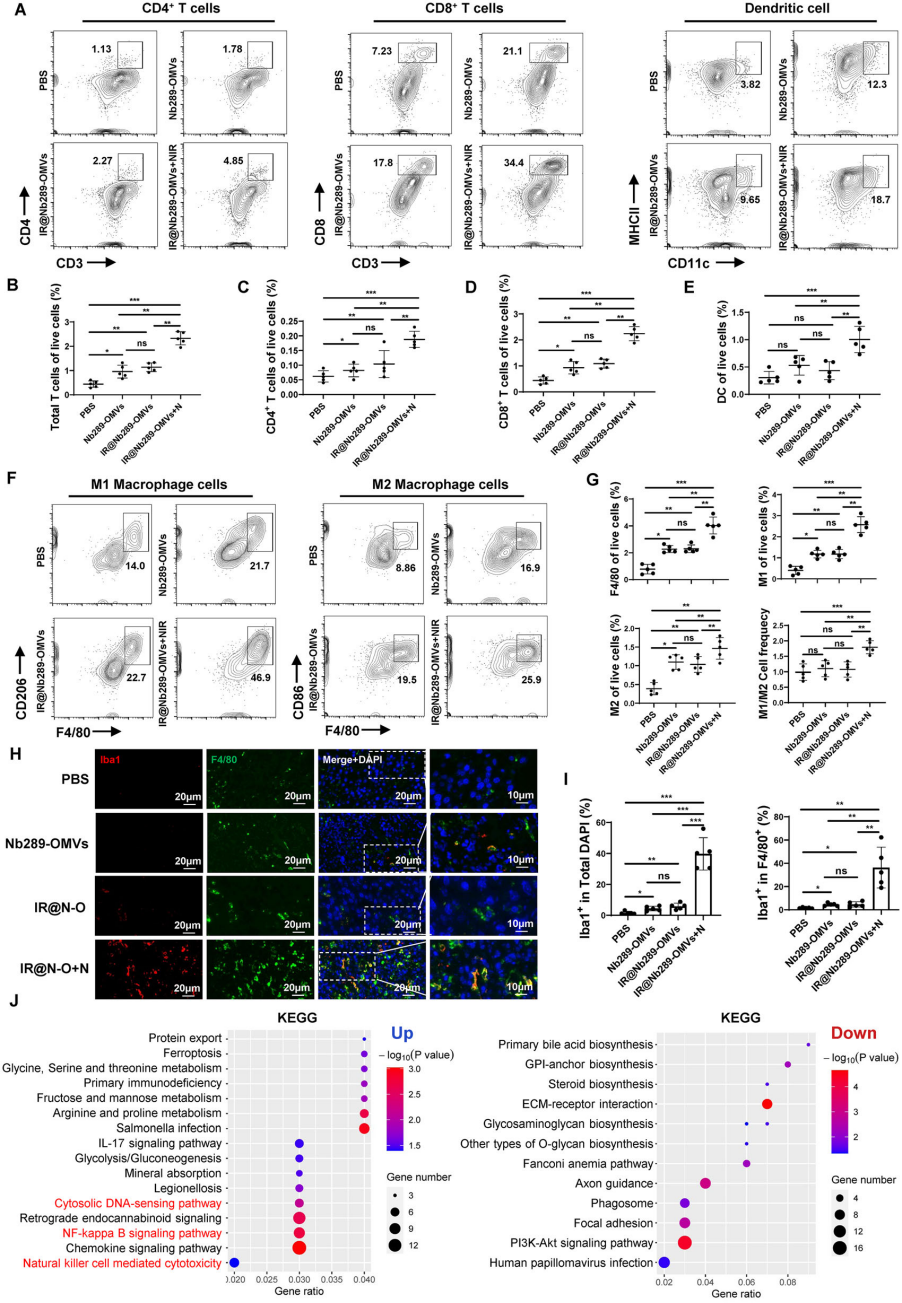
IR700@Nb289‐OMVs‐mediated photoimmunotherapy reprograms the TME. (A) Flow cytometry analysis of CD4^+^ T, CD8^+^ T and dendritic cells (DCs) in tumour tissues following treatment with PBS (200 µL), Nb289‐OMVs (1 × 10^11^ particles/injection), IR700@Nb289‐OMVs (1 × 10^11^ particles/injection) or IR700@Nb289‐OMVs (1 × 10^11^ particles/injection) + NIR (50 J/cm^2^) (*n* = 5 per treatment). The tissue samples were collected after three cycles of treatment. (B–E) Quantification of total T (CD3^+^), CD4^+^ T, CD8^+^ T and mature DCs within the tumours in (A). (F) Flow cytometry analysis of total and polarised Mφ (M1 and M2) in tumour tissues following different treatments (*n* = 5). (G) Quantification of total Mφ (F4/80^+^), M1 (F4/80^+^CD86^+^), M2 (F4/80^+^CD206^+^) and the ratio of M1/M2 in (F). (H) Expression and colocalisation of Iba1 and F4/80^+^ Mφ in tumour tissues after various treatments detected by immunofluorescence (*n* = 5). Scale bars, 20 or 10 µm as indicated. (I) Quantification for F4/80^+^ Mφ and Iba1^+^ staining. (J) KEGG pathway enrichment analysis of the upregulated and downregulated genes (*n* = 3). Enriched upregulated (left) and downregulated (right) pathways are shown. Data are presented as mean ± SD. Statistical significance was calculated using one‐way ANOVA with Tukey's post‐test (B–E, G, I). *p < 0.05, **p < 0.01 and ***p < 0.001. ANOVA, analysis of variance; NIR, near‐infrared; KEGG, Kyoto Encyclopedia of Genes and Genomes; OMV, outer membrane vesicle; PBS, phosphate buffered saline; SD, standard deviation; TME, tumour microenvironment.

Having demonstrated that IR700@Nb289‐OMVs plus NIR can significantly suppress and induce the ICD of CDH17‐positive colorectal and pancreatic cancers, we next comprehensively explored the underlying mechanisms involved. Based on the previous in vitro and in vivo data, we started by evaluating the microenvironment composition of Colon26 tumours upon treatment with Nb289‐OMVs alone, IR700@Nb289‐OMVs alone and IR700@Nb289‐OMVs plus NIR, all of which significantly inhibit tumour growth as shown in Figure 3H–P. Flow cytometry analysis uncovered that the infiltration of CD3^+^, CD4^+^ and CD8^+^ T cells was increased in treated tumours, among which the IR700@Nb289‐OMVs and NIR combo treatment resulted in the highest T cell recruitment, and no significant difference was observed between Nb289‐OMVs and IR700@Nb289‐OMVs treatment (Figure 4A–E and Figure S17). The number of mature dendritic cells (DCs; CD11c^+^MHCII^+^) in the tumours was also markedly elevated upon OMVs plus NIR treatment, with Nb289‐OMVs and IR700@Nb289‐OMVs alone inducing only a slight increase (Figure 4A–E). Moreover, the total macrophages (Mφ; F4/80^+^), M1 phenotypical Mφ (F4/80^+^CD86^+^) and M2 phenotypical Mφ (F4/80^+^CD206^+^) were all greatly increased in tumour tissues regardless of the treatment received when compared with PBS, but the OMVs plus NIR treatment triggered the greatest changes (Figure 4F, G). However, an increased ratio of M1/M2 in tumour tissues was only observed in the combo treatment group, suggesting that IR700@Nb289‐OMVs plus NIR has the greatest potential for tumour inhibition and TME reprogramming among all the therapeutic regimens explored in this study. Next, we examined whether these macrophages were activated and could potentially augment phagocytosis by determining the levels of ionised calcium‐binding adaptor molecule 1 (Iba1), a marker for macrophage activation and phagocytosis (Lu et al. 2022). Treatment with IR700@Nb289‐OMVs plus NIR increased significantly Iba1 expression in macrophages, whereas Nb289‐OMVs and IR700@Nb289‐OMVs administration just induced a slight but significant Iba1 upregulation in macrophages as compared with the control (Figure 4H, I), suggesting that tumour‐targeting OMVs can recruit and activate macrophages of the TME and that addition of IR700 can further boost this effect upon irradiation. Given the pronounced alterations observed in the macrophage population within the TME, it becomes clear that these immune cells are crucial for the antitumour activity promoted by engineered OMVs and IR700‐modified engineered OMVs plus NIR.

To elucidate the immune responses and view the global transcription profiling triggered by the IR700@Nb289‐OMVs plus NIR treatment, we also conducted RNA sequencing (RNA‐seq) on the tumour tissues upon administration of three IR700@Nb289‐OMVs plus NIR treatment cycles. Principal component analysis (PCA) showed that the variance of PC1/PC2 was less than 50%, indicating that the samples from each group were highly correlated and had low intersample variability (Figure S18A). To characterise the related pathways affected by OMVs plus NIR, Gene Ontology (GO) and Kyoto Encyclopedia of Genes and Genomes (KEGG) database were used. Differential gene expression analysis showed that 912 genes were differentially expressed after treatment (Figure S18B, C), with comprehensive alterations in the biological process (BP), cellular component (CC) and molecular function (MF) being involved (Figure S18D–F). Among them, the upregulated BP terms suggested that the identified enriched genes were involved in immune‐related processes, including the inflammatory response, innate immune response, defence response and leukocyte activation, etc., whereas those genes in the downregulated BP terms were involved in cell proliferation, cell cycle and cell growth (Figure S18D). Moreover, KEGG analysis suggested that the upregulated genes were highly associated with the cytosolic DNA‐sensing pathway, NF‐κB signalling pathway, chemokine signalling pathway and natural killer cell‐mediated cytotoxicity, whereas genes related to the PI3K/AKT pathway were prominently downregulated, which is consistent with cell proliferation inhibition (Figure 4J). These results collectively indicate that various immune responses are involved in the tumour suppression and TME reprogramming responses triggered by IR700@Nb289‐OMVs plus NIR, which highly aligns with the results of flow cytometry for TME analysis.

### 3.5 Nb289‐OMVs and IR700@Nb289‐OMVs‐Mediated Tumour Suppression Require cGAS‐STING Pathway Activation

**FIGURE 5:**
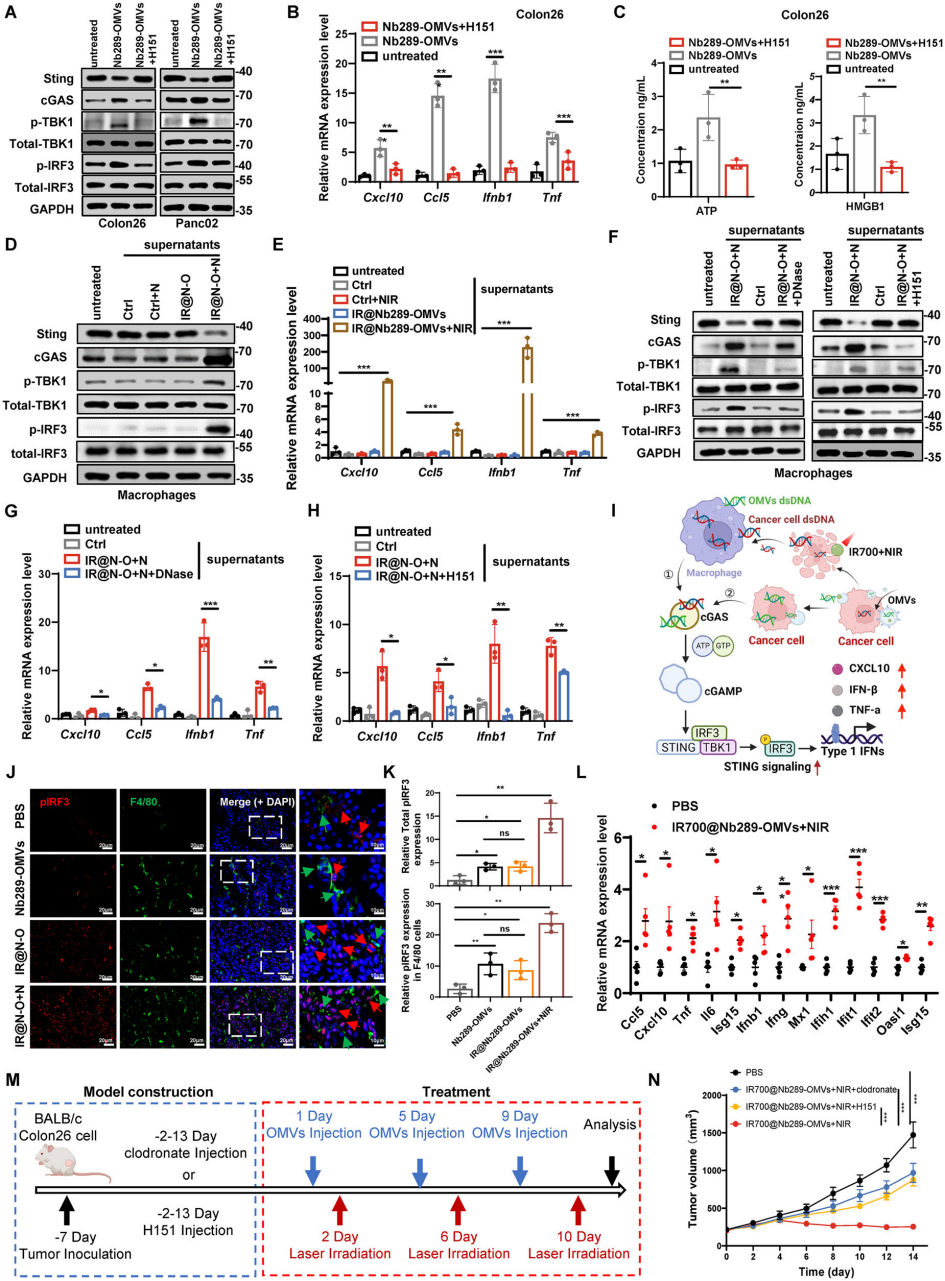
cGAS‐STING pathway is crucial for Nb289‐OMVs and IR700@Nb289‐OMVs‐initated tumour suppression. (A) Western blot analysis of the STING pathway in cancer cells treated overnight with PBS, Nb289‐OMVs (1 × 10^10^ particles/mL) or Nb289‐OMVs (1 × 10^10^ particles/mL) + H151 (2 µM). (B) mRNA expression of STING pathway downstream genes in Colon26 cells treated with Nb289‐OMVs alone or in combination with H151 (*n* = 3). (C) Changes in ATP and HMGB1 release in Colon26 cancer cells treated with Nb289‐OMVs alone or in combination with H151 (*n* = 3). (D) Assessment of the STING pathway in macrophages treated with preconditioned supernatants collected from Colon26 cells upon the indicated treatments (Ctrl + N: PBS plus NIR irradiation alone; IR@N‐O: IR700@Nb289‐OMVs alone; IR@N‐O + N: combo IR700@Nb289‐OMVs plus NIR irradiation) (*n* = 3). (E) mRNA expression of STING pathway downstream genes in macrophages upon the indicated treatments (*n* = 3). (F) Western blot analysis of the STING pathway in macrophages treated with various preconditioned supernatants containing DNase I or H151 (*n* = 3). (G, H) mRNA expression of STING pathway downstream genes in macrophages treated with various preconditioned supernatants containing DNase I or H151 as described in (E) (*n* = 3). (I) Schematic diagram of the activation of the STING pathway by Nb289‐OMVs or IR700@Nb289‐OMVs plus NIR. (J) Immunofluorescence analysis of p‐IRF3 and F4/80 marker in tumour tissues after various OMV‐related treatments. The inlets show the colocalisation of p‐IRF3 and F4/80^+^ Mφ. (K) Quantification of total expression of p‐IRF3 and the colocalisation of p‐IRF3 and F4/80^+^ Mφ for (J). (L) Relative expression of ISGs in tumours after treatment with IR700@Nb289‐OMVs plus NIR (*n* = 5). (M) Treatment schedule for macrophage depletion and STING inhibition in tumour‐bearing mice treated with PBS, IR700@Nb289‐OMVs (1 × 10^11^ particles/injection) + NIR (50 J/cm^2^) + clodronate (200 µL, 5 mg/mL, peritumoural injection for 7 consecutive days), IR700@Nb289‐OMVs (1 × 10^11^ particles/injection) + NIR (50 J/cm^2^) + H151 (10 mg/kg, intraperitoneal injection for 7 consecutive days) and IR700@Nb289‐OMVs (1 × 10^11^ particles/injection) + NIR (50 J/cm^2^) (*n* = 5 per group). (N) Tumour growth curves upon the indicated treatments coupled with macrophage depletion and STING inhibition (*n* = 5 per treatment). Data are presented as mean ± SD. Statistical significance was calculated using two‐tailed unpaired *t* test analysis (L) or one‐way ANOVA with Tukey's post‐test (B, C, E, G, H, K) or two‐way ANOVA (N). *p < 0.05, **p < 0.01 and ***p < 0.001. ANOVA, analysis of variance; cGAS, cyclic GMP‐AMP synthase; HMGB1, high‐mobility group B1; NIR, near‐infrared; OMV, outer membrane vesicle; PBS, phosphate buffered saline; SD, standard deviation; STING, stimulator of interferon genes; TME, tumour microenvironment.

The RNA sequencing data highlighted that innate immune responses were significantly upregulated by IR700@Nb289‐OMVs plus NIR treatment, particularly via the cytosolic DNA‐sensing and NF‐κB signalling pathways (Figure 4J). Noteworthily, these pathways point to a crucial innate immune pathway for antitumour immunity, the cGAS‐STING pathway, which effectively responds to cytosolic dsDNA and promotes proinflammatory effects based on type I INF‐stimulated genes (ISGs) (Samson and Ablasser 2022). OMVs inherit various components from their parental bacteria, including outer membrane proteins, phospholipids and nucleic acids (DNA and RNA) (Bitto et al. 2017). Two important studies have demonstrated that extracellular membrane vesicles (MVs) from the gut microbiota and *Candida albicans* promote the activation of the cGAS/STING/IFN‐I axis and enhance antiviral immunity (Erttmann et al. 2022; Brown Harding et al. 2024). In addition, cell debris produced after treatment with paclitaxel or ultraviolet irradiation, two typical inducers of ICD (Lau et al. 2020; Zang et al. 2023), can efficiently transactivate the cGAS‐STING pathway of APCs, such as DCs or macrophages (Ahn et al. 2018; de Mingo Pulido et al. 2021). Hence, we hypothesised that (i) Nb289‐OMVs could directly activate the cGAS‐STING pathway in cancer cells and possibly in macrophages upon OMVs uptake, and (ii) IR700@Nb289‐OMVs could transactivate the cGAS‐STING pathway in recruited macrophages in the TME through cancer cell debris produced by NIR irradiation‐induced ICD like paclitaxel and irradiation. To address these hypotheses, we first examined whether the Nb289‐OMVs contained nucleic acids. Agarose gel electrophoresis clearly showed the presence of nucleic acids in the Nb289‐OMVs, which were similar to those present in the bacterial supernatants (Figure S19A). Furthermore, we determined the copy number of bacterial DNA through the detection of *16S* rDNA, which suggested that each Nb289‐OMV contained approximately 20 copies of *16S* rDNA (Figure S19B, C). Next, Colon26 and Panc02 cells were incubated with Nb289‐OMVs and the indicators for the cGAS‐STING pathway were evaluated. Overall, cGAS, phosphorylated TBK1 and IRF3 were upregulated upon treated, events that were accompanied by STING downregulation, which indicated that the cGAS‐STING pathway was activated (Figure 5A). Of note, the addition of the STING antagonist, H151, could abolished these effects in both cell lines (Figure 5A). The activation of the STING pathway can drive the expression of downstream type I IFN cytokines and ISGs. Indeed, incubation with Nb289‐OMVs significantly induced the mRNA expression of several ISGs such as *CXCL10*, *CCL5*, *IFNB1* and *TNF‐α*, and H151 reversed their expression in cancer cells (Figure 5B). These results demonstrate that the activation of cGAS‐STING pathway in cancer cells is induced by Nb289‐OMVs. Since chemotherapeutic drugs can induce ICD through STING activation (Wang‐Bishop et al. 2020; Wang et al. 2019), and we have observed that Nb289‐OMVs can induce ICD in the tumour cells in vitro and in vivo (Figure 3B, K), we wonder whether STING activation by Nb289‐OMVs promotes ICD in Colon26 cells. In line with our previous results, we indicated that Nb289‐OMVs induced the translocation of CRT/ERp57 and increased the secretion of ATP and HMGB1 in colon cancer cells, implying ICD induction; however, the STING inhibitor H151 almost abolished these effects (Figure 5C and Figure S20A, B), implying that Nb289‐OMVs not only activate the cGAS‐STING pathway and enhance the expression of type I IFNs and ISGs but also lead to ICD upon STING activation. TAMs are the major target cells of STING agonists, and STING activation in macrophages within the TME has exhibited tumour‐suppressive potency (Pan et al. 2020; Chin et al. 2020). OMVs in the TME might be ingested by TAMs through phagocytosis. Thus, we investigated next whether the Nb289‐OMVs could also activate the STING pathway in macrophages. The STING pathway biomarkers, such as cGAS, phosphorylated STING and IRF3, were markedly elicited, and clear nuclear localisation of IRF3 was observed when macrophages isolated from the mouse peritoneal cavity were incubated directly with Nb289‐OMVs, demonstrating that internalised OMVs can activate the cGAS‐STING pathway in macrophages (Figure S21A, B). Collectively, these results indicate that Nb289‐OMVs alone can initiate the activation of the cGAS/STING/IFN‐I axis, which may result in ICD induction in cancer cells. OMVs may also stimulate the cGAS‐STING pathway in TAMs upon their engulfment, suggesting the potential of OMVs as natural STING agonists.

To address whether IR700@Nb289‐OMVs could transactivate the cGAS‐STING pathway in TME macrophages through cancer cell debris produced by NIR irradiation‐induced ICD, primary macrophages were treated with supernatants collected from Colon26 cells treated with NIR irradiation alone, IR700@Nb289‐OMVs alone and OMVs plus NIR. Indeed, only the supernatants from Colon26 cells treated with IR700@Nb289‐OMVs plus NIR promoted the activation of the STING pathway, as indicated by the upregulated levels of cGAS/p‐TBK1/p‐IRF3 and downregulated STING (Figure 5D and Figure S21C); accompanied by STING activation, ISGs were significantly induced (Figure 5E), implying that the supernatants from Colon26 treated with OMVs plus NIR contain the components for activation of cGAS‐STING pathway, such as abundant DNA released from dying cells with ICD. Of note, these signalling changes in macrophages were completely abrogated upon incubation with supernatants from irradiated Colon26 cells pretreated with DNase, similar to the effects observed upon treatment with the STING antagonist H151 (Figure 5F) demonstrating that DNA released from dying cells with ICD after NIR irradiation is the key component for STING activation in macrophages. Moreover, incubation with either DNases or H151 reversed the increased STING signalling‐induced expression of the ISGs (Figure 5G, H). Taken together, these in vitro results disclose that OMVs can directly activate the STING pathway and consequently promote ICD. Moreover, IR700‐modified nanobody‐engineered OMVs can transactivate the STING pathway in macrophages via the abundant release of DNA from NIR irradiation‐induced dying cancer cells (Figure 5I).

To further confirm the above described in vitro results, we subsequently assessed the STING pathway status in tumour tissues collected from mice treated with Nb289‐OMVs, IR700@Nb289‐OMVs or OMVs plus NIR. The phosphorylated IRF3 (p‐IRF3) localised in nuclei as an indicator for STING activation was examined. An overall increase was observed for the total expression of p‐IRF3 in the tumour mass and for its specific expression in F4/80^+^ TAMs after treatment (Figure 5J, K). Notably, combo OMVs plus NIR treatment resulted in the most significant increase in p‐IRF3 (Figure 5J, K). Moreover, a panel of ISGs were prominently upregulated after the combo treatment (Figure 5L), as well as some systemic proinflammatory cytokines, such as tumour necrosis factor α (TNF‐α), IFN‐γ and interleukin‐6 (IL‐6) (Figure S22A–C). These in vivo results are highly in agreement with the in vitro data, thereby demonstrating that the cGAS‐STING pathway is crucial for OMVs‐ and IR700‐modified OMV‐mediated tumour suppression, potentially via activation of the systemic antitumour immunity.

Finally, to clarify the important role of the STING pathway in TAMs for IR700@Nb289‐OMVs‐mediated antitumour activity, we used the STING antagonist H151 and the macrophage depletion agent clodronate to determine whether they could reverse the antitumour effect of IR700@Nb289‐OMVs plus NIR. Peritumoural administration of clodronate was found to effectively deplete TAMs without affecting the tumour growth (Figure S23A–D), whereas H151 effectively reversed the antitumour activity of MSA2, a well‐documented STING agonist (Pan et al. 2020) (Figure S23D). Colon26 tumour‐bearing mice were then administered IR700@Nb289‐OMVs plus NIR, along with clodronate or H151 (Figure 5M). Both clodronate and H151 significantly abrogated the tumour‐suppressive effect of IR700@Nb289‐OMVs plus NIR (Figure 5N, S24A, B), indicating the importance of the STING pathway in TAMs for OMVs and NIR‐initiated antitumour activity. Collectively, we demonstrate that CDH17‐targeting nanobody‐engineered OMVs can directly initiate the activation of the STING pathway in cancer cells, which in turn induces ICD occurrence. In addition, IR700‐modified Nb289‐OMVs can further boost the STING activation in TAMs by inducing DNA release from dying cancer cells promoted by NIR irradiation.

### 3.6 IR700@Nb289‐OMVs Plus NIR Enhance the Antitumour Efficacy of Immune Checkpoint Blockades

**FIGURE 6:**
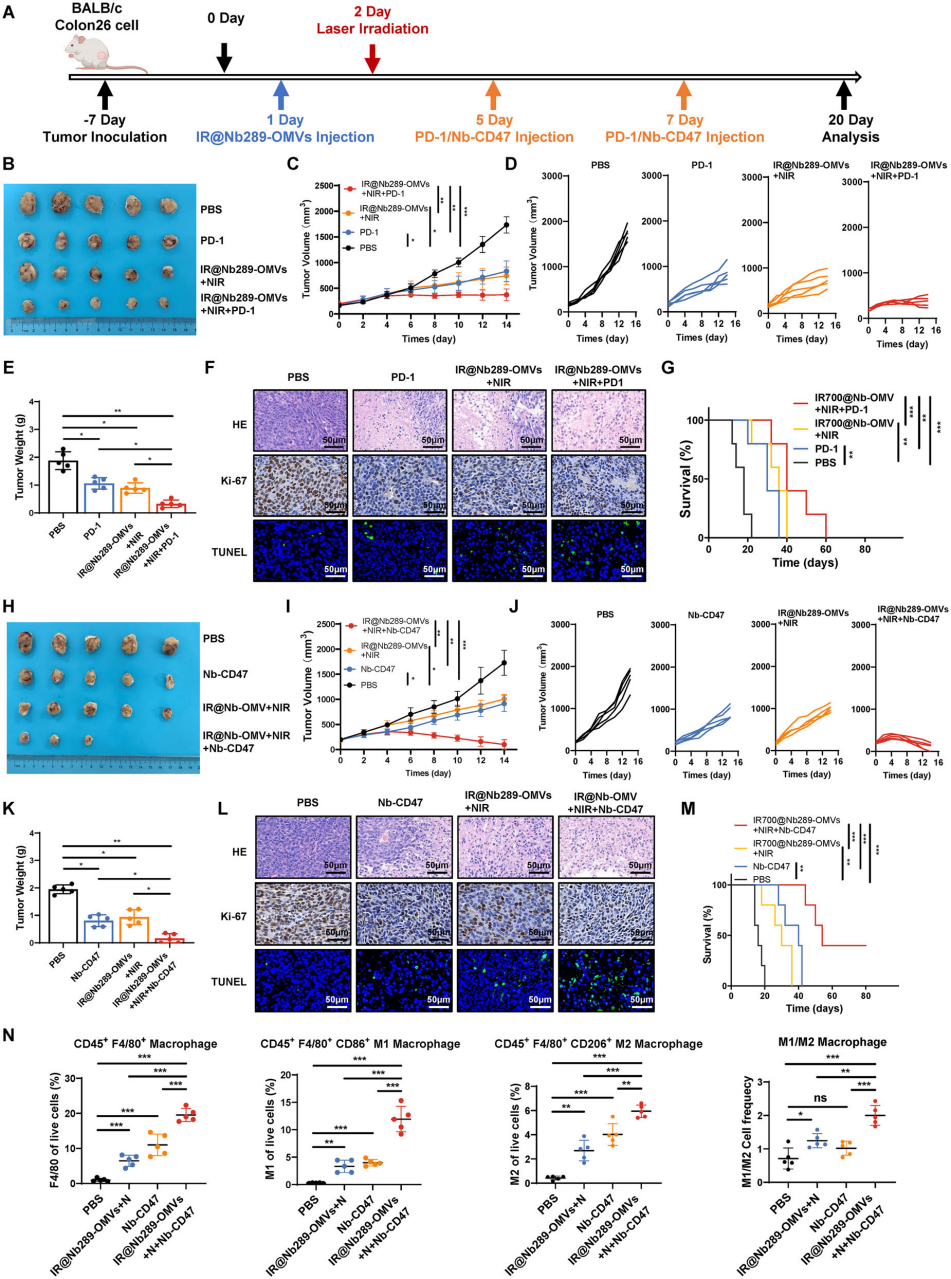
One cycle of engineered OMVs‐mediated photoimmunotherapy significantly augments the antitumour efficacy of immune checkpoint blockade therapies. (A) Schematic representation of the combo treatment schedule with PBS (200 µL), PD‐1 antibodies (12.5 mg/kg)/Nb‐CD47 (10 mg/kg), IR700@Nb289‐OMVs (1 × 10^11^ particles/injection) + NIR (50 J/cm^2^), and IR700@Nb289‐OMVs (1 × 10^11^ particles/injection) + NIR (50 J/cm^2^) + PD‐1 antibodies (12.5 mg/kg)/Nb‐CD47 (10 mg/kg). The mice were treated with NIR for one cycle, followed by two injections of PD‐1 antibodies or Nb‐CD47 (*n* = 5 per group). (B) Sizes of the tumours collected at the end of the experiment with PD‐1 antibodies (*n* = 5). (C) Tumour growth curves under various treatments regiments with the PD‐1 antibody (*n* = 5). (D) Individual tumour growth curves at the end of the experiment with the PD‐1 antibody (*n* = 5). (E) Tumour weights of the different treatment groups with the PD‐1 antibody (*n* = 5). (F) Survival analysis of tumour‐bearing mice receiving OMV‐based photoimmunotherapy and/or PD‐1 antibody (*n* = 5). (G) H&E, Ki67 and TUNEL staining of the tumours from (B) (*n* = 5). (H) Tumour appearance at the end of the experiment with the CD47 nanobodies (*n* = 5). (I) Tumour growth curves for various treatments with the CD47 nanobodies (*n* = 5). (J) Individual tumour growth curves at the end of the experiment with the CD47 nanobodies (*n* = 5). (K) Tumour weights of the different groups with the CD47 nanobodies from (H) (*n* = 5). (L) Survival analysis of the tumour‐bearing mice receiving OMV‐based photoimmunotherapy and/or CD47 nanobodies (*n* = 5). (M) H&E, Ki67 and TUNEL staining of the tumours from (H) (*n* = 3–5). (N) Changes in the macrophage populations within the TME at the end of the experiment were determined by flow cytometry (*n* = 5). Three days after the end of the treatment, the tumour tissues were collected for flow cytometry analysis. Data are presented as mean ± SD. The data are presented as mean ± SD. Statistical significance was calculated using one‐way ANOVA with Tukey's post‐test (E, K, N) or two‐way ANOVA (C, I) or Mantel–Cox test (G, M). *p < 0.05, **p < 0.01 and ***p < 0.001. ANOVA, analysis of variance; H&E, haematoxylin and eosin; OMV, outer membrane vesicle; PBS, phosphate buffered saline; SD, standard deviation; TME, tumour microenvironment.

We disclose that IR700@Nb‐OMVs plus NIR show the highest antitumour efficiency in CDH17‐positive Colon26 and Panc02 models and profoundly reprogram the TME including the increase of T cell infiltration, mature DCs and recruitment of macrophages with the clear switch of M2 to M1 phenotype; meanwhile, cGAS‐STING pathway is broadly activated. Despite the important antitumour proprieties, it remained unclear whether this new therapeutic approach could be coupled and synergise with clinically relevant immune checkpoint blockade therapies against PD‐1/PDL‐1 or CD47/SIRPα axes, two pivotal immune checkpoints with extensive clinical focuses. Therefore, we designed an in vivo treatment strategy that combined IR700@Nb289‐OMVs plus NIR irradiation with a PD‐1 antibody or CD47 nanobody (Sockolosky et al. 2016) (Figure 6A). We found out that one cycle of IR700@Nb289‐OMVs plus NIR combined with two injections of PD‐1 blockade remarkably hampered the development of the Colon26 cancer model compared with any of these therapies alone, both of which also exhibited tumour suppression potential compared with the control PBS treatment (Figure 6B–E). H&E, Ki67 and TUNEL staining of tumour tissues revealed that OMVs photoimmunotherapy combined with PD‐1 blockade significantly reduced cell proliferation while promoted cell necrosis and apoptosis (Figure 6F and S25A). Similarly, mice treated with IR700@Nb289‐OMV plus NIR combined with PD‐1 blockade had a longer lifespan than those in the other treatment groups (Figure 6G). Safety profiles based on body weight changes, H&E staining of various major organs and blood tests showed that none of the treatments was associated with obvious adverse effects (Figures S25B–D, S26).

Next, we assessed the antitumour efficacy of IR700@Nb289‐OMVs plus NIR irradiation and CD47 nanobodies using the same tumour model. One cycle of IR700@Nb289‐OMV plus NIR or two injections of the CD47 nanobody alone showed similar tumour inhibitory efficacy. Surprisingly, the combination of these two treatments not only retarded tumour growth more effectively than each treatment alone but also resulted in tumour disappearance in 40% of the mice (2/5) (Figure 6H–K, M). In addition, combo therapy with CD47 blockade significantly inhibited cell proliferation, and increased necrosis and apoptosis of the tumour tissues (Figure 6L and Figure S27A). Similarly, none of the treatments tested resulted in discernible side effects based on mouse body weight, pathological analysis of major organs or blood tests (Figures S27B–D, S28), demonstrating the good biosafety of engineered OMVs‐mediated photoimmunotherapy with CD47 blockade. Given the strong antitumour activity induced by IR700@Nb289‐OMVs plus NIR in combination with a CD47 nanobody, we further analysed the changes in the TME after treatment with these regimens. Flow cytometry analysis of the collected tumours showed that this combo treatment greatly increased the recruitment of macrophages (CD45^+^F4/80^+^), in particular of the M1 phenotype, as compared with the IR700@Nb289‐OMVs plus NIR or the CD47 nanobody alone although both of treatments alone have also resulted in increased recruitment of macrophages and the M1 phenotype (Figure 6N), indicating that the CD47 blockade combo treatment can promote the greatest innate immune activation. Furthermore, the combinational regimen was also found to slightly increase the population of CD4^+^ and CD8^+^ T cells when compared with the PBS control, but this response was similar to that observed with the individual therapies (Figure S29), thereby implying that innate antitumour immunity may be more relevant in this therapeutic context than adaptive immunity.

Altogether, we demonstrate that IR700@Nb289‐OMVs plus NIR can synergistically enhance the antitumour efficacy of PD‐1 and CD47 blockade therapies; it seems that, combined with the CD47 blockade, IR700@Nb289‐OMVs plus NIR can exert better antitumour activity and partially result in tumour eradication, possibly inducing durable antitumour immune memory.

### 3.7 Engineered OMV‐Mediated Photoimmunotherapy plus CD47 Blockade Inhibit the Progression of Metastatic Cancers and Produce Sustained Antitumour Immune Memory

**FIGURE 7:**
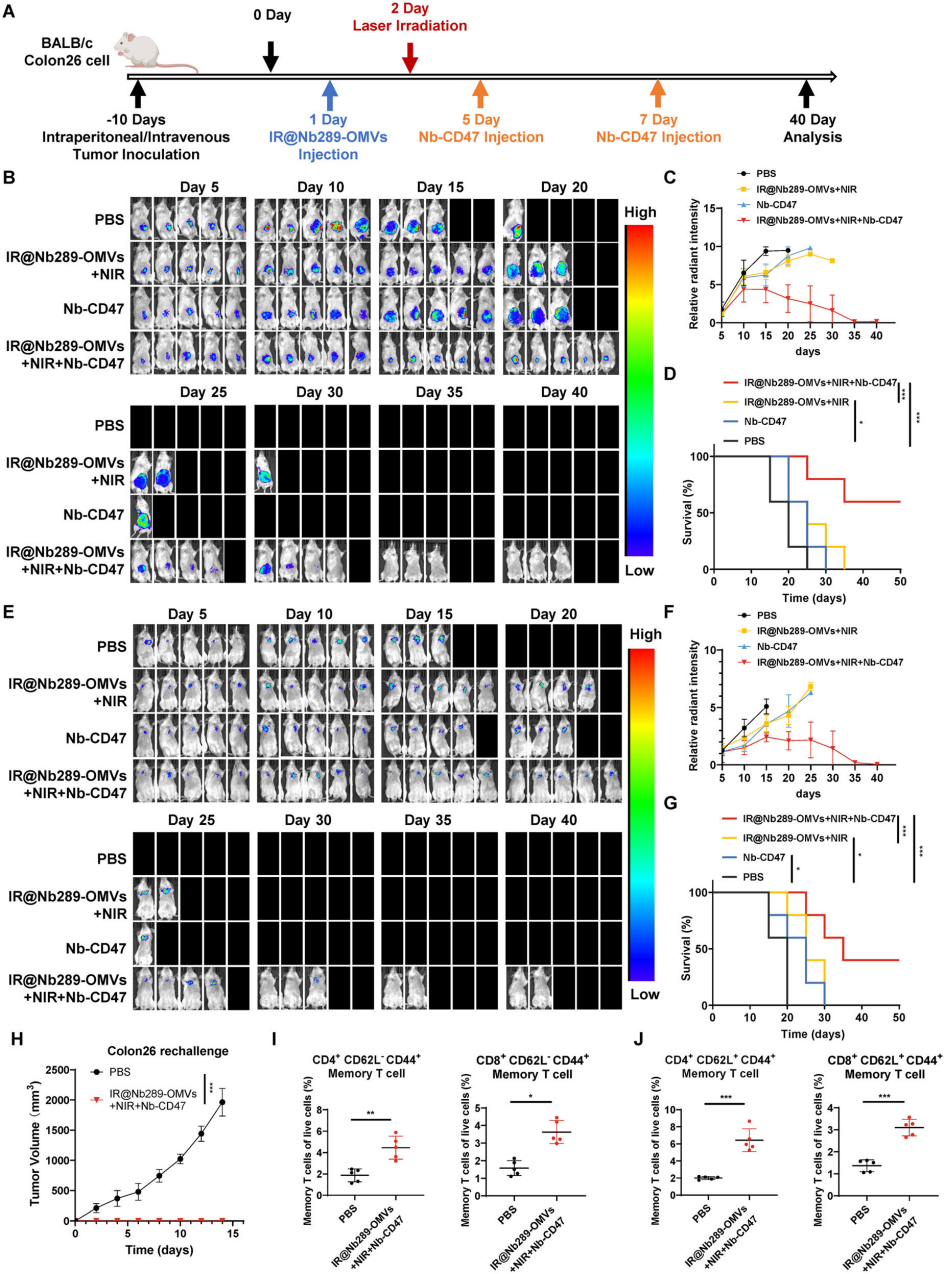
Engineered OMVs‐mediated photoimmunotherapy with CD47 nanobody synergistically inhibits the progression of metastatic CRC and elicits sustained immune memory. (A) Schematic representation of the combination treatment schedule with PBS (200 µL), IR700@Nb289‐OMVs (1 × 10^11^ particles/injection) + NIR (50 J/cm^2^), Nb‐CD47 (10 mg/kg) and IR700@Nb289‐OMVs (1 × 10^11^ particles/injection) + NIR (50 J/cm^2^) + Nb‐CD47 (10 mg/kg). Mice were treated with NIR irradiation for one cycle, followed by two injections of Nb‐CD47. (B) Bioluminescence imaging of abdominal metastatic colorectal tumours with various treatments (*n* = 5). (C) Quantification of bioluminescent signals to estimate abdominal metastatic tumour burden. (D) Survival curves of abdominal metastatic tumour‐bearing mice receiving different treatments (*n* = 5). (E) Bioluminescence imaging of metastatic lung colorectal tumours treated with various regimens (*n* = 5). (F) Quantification of the bioluminescent signal to estimate the lung metastatic tumour burden (*n* = 5). (G) Survival curves for lung metastatic tumour‐bearing mice upon different treatment regimens (*n* = 5). (H) Tumour growth curves for mice rechallenged with Colon26‐Luc cancer cells 90 days after tumour eradication (*n* = 5). (I) Flow cytometry analysis of effector memory T cells (CD62L^−^CD44^+^) in the spleens of rechallenged or age‐matched control mice at the end of the experiment (*n* = 5). (J) Flow cytometry analysis of central memory T cells (CD62L^+^CD44^+^) in the spleens of rechallenged or age‐matched control mice at the end of the experiment (*n* = 5). Data are presented as mean ± SD. Statistical significance was calculated using two‐tailed unpaired *t* test analysis (I, J) or two‐way ANOVA (H) or Mantel–Cox test (D, J). *p < 0.05, **p < 0.01 and ***p < 0.001. ANOVA, analysis of variance; CRC, colorectal; NIR, near‐infrared; OMV, outer membrane vesicle; PBS, phosphate buffered saline; SD, standard deviation; TME, tumour microenvironment.

Because IR700@Nb289‐OMVs plus NIR irradiation combined with CD47 blockade showed better antitumour outcomes, we further extended this combination therapy to metastatic colorectal cancer models. Colorectal cancer tends to spread to the liver, lungs and abdominal cavity to form metastatic foci. Hence, we attempted to determine whether this combination regimen is also effective against metastatic colorectal cancers. Given that IR700 can penetrate the lungs and abdominal wall, we conducted subsequent experiments using lung and abdominal metastatic colorectal models. Colon26‐Luc cells were intraperitoneally injected into the mouse abdominal cavities or were intravenously administered to establish abdominal or lung metastatic models; bioluminescence imaging was used to monitor tumour progression and the therapeutic effects (Figure 7A). Individual CD47 nanobody or IR700@Nb289‐OMVs plus NIR irradiation resulted in a slight delay in tumour progression in the abdominal metastatic model compared with the control treatment (Figure 7B–D). In contrast, administration of IR700@Nb289‐OMVs plus NIR irradiation along with CD47 nanobodies significantly impeded metastatic tumour progression and markedly extended the mouse lifespan, ultimately resulting in the complete elimination of abdominal metastatic foci in 60% of the tumour‐bearing mice (3/5) (Figure 7B–D). Similar results were obtained in a lung metastasis model induced by the same Colon26‐Luc cells, with the combo treatment significantly repressing tumour development in the lungs and prolonged mouse survival among all the groups, eventually leading to tumour disappearance in 40% of the mice (2/5) (Figure 7E–G). CD47 nanobody or IR700@Nb289‐OMVs plus NIR irradiation alone slightly inhibited metastatic lung and abdominal tumours, and extended survival, but all mice succumbed due to the high tumour burden at the end of the experiment (Figure 7B, D, E, G). Since the IR700@Nb289‐OMVs plus NIR irradiation and CD47 nanobody combo regimen could partially result in the complete elimination of CRC metastatic tumours, we further rechallenged the mice whose tumours had been eliminated to determine whether immune memory was generated. Mice with eliminated tumours and age‐matched control animals were rechallenged with Colon26‐Luc cells being placed on their flanks 90 days after tumour disappearance. No tumour growth was observed in the surviving mice previously treated with the combo regimen, whereas tumours rapidly developed in the age‐matched control mice (Figure 7H and Figure S30). These findings indicate that this combinational therapeutic approach initiates a durable T‐cell antitumour immune memory, which potentially prevents tumour recurrence. In line with this hypothesis, both the effector (CD62L^−^CD44^+^) and central memory T cells (CD62L^+^CD44^+^) in the CD4^+^ or CD8^+^ T cell compartments were significantly increased in the previously treated mice (Figure 7I, J and Figure S31), clearly indicating the production of antitumour immune memory T cells.

Collectively, these results demonstrate that engineered OMV‐mediated photoimmunotherapy combined with CD47 nanobodies holds enormous potential to suppress primary and metastatic CDH17‐positive CRC and promote complete tumour eradication. Durable antitumour immune memory can be established in vivo, which can potentially prevent tumour relapse.

## 4 Discussion

Our results demonstrated that Nb289‐engineered OMVs significantly improved the therapeutic efficacy, safety profile and tumour‐bearing mice tolerance, even at higher doses and administration frequencies. The antitumour activity of these engineered OMVs was further enhanced when loaded with IRDye700DX (IR700), a photoimmunotherapeutic agent, upon irradiation. Mechanistically, we revealed that engineered OMVs directly induced activation of cGAS‐STING pathway in tumour cells and potentially in TAMs. This activation was mediated by internal dsDNA inherited from parental bacteria upon internalisation, resulting in ICD of cancer cells. Loading IR700 into engineered OMVs synergistically promoted ICD induction upon irradiation, leading to the abundant release of endogenous dsDNA into the TME. This comprehensively stimulated STING activation in TAMs and enhanced antitumour immunity. Importantly, when combined with a CD47 nanobody, IR700@Nb289‐OMVs markedly extended survival and partially eradicated tumours in subcutaneous and metastatic (lung and peritoneal cavity) CRC mouse models. This combination therapy evoked the formation of long‐term antitumour immune memory.

Erttmann et al. (2022) disclosed that OMVs from the gut microbiota can stimulate the cGAS‐STING pathway through the release of internal dsDNA in host cells and evoke a systemic antiviral response. They also found that membrane structures in MVs can protect internal dsDNA from degradation by extracellular nucleases (Erttmann et al. 2022), suggesting excellent OMVs systemic stability, which is conducive to cGAS‐STING activation. Recently, Brown Harding et al. (2024) uncovered that extracellular vesicles from *C. albicans* can also trigger the cGAS‐STING pathway in macrophages to combat fungus‐impending infections. These studies suggest that OMVs containing dsDNA from the parental bacteria have considerable potential as robust cGAS‐STING agonists that prime antitumour immunity and suppress tumour development. Our study confirmed that engineered OMVs contained DNA from parental bacteria and efficiently stimulated the cGAS‐STING pathway in cancer cells and macrophages both in vitro and in vivo. Engineered OMVs alone showed significant tumour regression and TME reprogramming activity, suggesting their potential as strong STING agonists with reduced systemic side effects compared to untargeted STING agonists.

The combination of IR700@Nb289‐OMVs with CD47 blockade not only significantly repressed primary and metastatic tumours but also partially eliminated them and produced durable antitumour immune memory. These findings suggest that cGAS‐STING agonists combined with CD47 blockades may be an alternative strategy for antitumour immunotherapy. Kosaka et al. (2021) reported that an anti‐CD47 monoclonal antibody combined with the STING agonist cGAMP (intratumourally administrated) could efficiently enhance the systemic antitumour immune response, prevent tumour metastasis and partially cure tumour‐bearing mice, which is consistent with our results. In another report, Shi et al. (2020) revealed that *Bifidobacteria* from the gut microbiota can colonise tumours and stimulate STING signals, which can greatly improve the efficacy of anti‐CD47 immunotherapy, reminiscent of STING activation in host cells by OMVs secreted by the gut microbiota for antiviral infection in Erttmann's study (Erttmann et al. 2022). Taken together, these findings suggest that STING agonists, including OMVs, when used in combination with innate immune checkpoint inhibitors may be a valuable and generalisable strategy for cGAS/STING‐based immunotherapy.

We genetically modified OMVs with CDH17‐targeting nanobodies, imparting them with tumour‐homing capabilities and improved antitumour performance and biosafety. Encapsulation of IR700 further strengthened the antitumour activity of OMVs and shortened the treatment course. Mechanistically, we demonstrated that OMVs act as natural STING agonists, specifically delivering internal dsDNA into cancer cells and triggering activation of the cGAS/STING/IRF3/IFN‐I axis and ICD occurrence. IR700 loading boosted ICD induction in cancer cells upon irradiation and transactivated the cGAS/STING/IRF3/IFN‐I axis in TAMs by promoting the release of endogenous dsDNA from dying cancer cells. TME remodelling by engineered OMVs or IR700‐loaded engineered OMVs‐mediated photoimmunotherapy resulted in increased recruitment and activation of TAMs and the M1 phenotype, synergistically maximising antitumour efficacy with CD47 blocking therapies in primary and metastatic tumour models.

Although our study focused on the antitumour mechanism of OMVs and cGAS‐STING activation in TAMs, future research should investigate the potential involvement of other innate immune cells, such as dendritic cells. Additionally, further studies are needed to determine the extent to which similar effects can be achieved in human tumours.

## Author Contributions

**Peng Xia**: Conceptualization (lead), data curation (lead), formal analysis (equal), resources (lead), writing–original draft (lead). **Chengming Qu**: conceptualization (equal), data curation (lead), resources (equal). **Xiaolong Xu**: data curation (supporting), formal analysis (supporting), validation (supporting). **Ming Tian**: data curation (supporting), software (supporting), supervision (supporting). **Zhifen Li**: formal analysis (supporting), validation (supporting). **Jingbo Ma**: formal analysis (supporting), validation (supporting). **Rui Hou**: investigation (supporting), validation (supporting). **Han Li**: project administration (supporting), writing–original draft (supporting). **Felix Rückert**: formal analysis (supporting), supervision (supporting). **Tianyu Zhong**: funding acquisition (equal). **Liang Zhao**: formal analysis (supporting), validation (supporting). **Yufeng Yuan**: investigation (supporting), supervision (supporting). **Jigang Wang**: formal analysis (supporting), supervision (supporting). **Zhijie Li**: conceptualization (lead), data curation (lead), formal analysis (lead). **Zhijie Li**: conceptualization (lead), data curation (lead), formal analysis (lead).

## Ethics Statement

All animal experiments were approved by the Institutional Animal Care and Use Committee (IACUC) of Shenzhen People's Hospital and performed in accordance with relevant institutional and national guidelines and regulations (AUP‐220516‐LZJ‐0346‐01 and AUP‐230220‐LZJ‐0109‐01).

## Consent

The authors have nothing to report.

## Conflicts of Interest

The authors declare no conflicts of interest.

## Supporting information



Supporting Information

## Data Availability

The main data supporting the results in this study are available within the article and its Supporting Information. High‐throughput‐sequencing data are available through the GEO database via the accession number GSE272295. The data that support the findings of this study are available from the corresponding author upon reasonable request.
