# Supplementary material for: Nanobody Engineered and Photosensitiser Loaded Bacterial Outer Membrane Vesicles Potentiate Antitumour Immunity and Immunotherapy
Source: J Extracell Vesicles. 2025 Apr 16;14(4):e70069. doi: 10.1002/jev2.70069 (PMC12003094; doi:10.1002/jev2.70069)
Supplement: Supplementary file 1 — Supporting Information [file JEV2-14-e70069-s001.docx]

**Nanobody Engineered and Photosensitiser Loaded Bacterial Outer Membrane Vesicles Potentiate Antitumour Immunity and Immunotherapy**

Peng Xia^1, 2, 3#^, Chengming Qu^1#^, Xiaolong Xu^2#^, Ming Tian^1#^, Zhifen Li^4^, Jingbo Ma^2^, Rui Hou^5^, Han Li^3^, Felix Rückert^6^, Tianyu Zhong^7*^, Liang Zhao^8, 9*^, Yufeng Yuan^1^^*^, Jigang Wang^2, 10, 11, 12*^, Zhijie Li^2*^

^1^Zhongnan Hospital of Wuhan University, TaiKang Center for Life and Medical Sciences, Clinical Medicine Research Center for Minimally Invasive Procedure of Hepatobiliary & Pancreatic Diseases of Hubei Province, Wuhan University, Wuhan, Hubei 430071, P. R. China

^2^Department of Critical Care Medicine, Guangdong Provincial Clinical Research Center for Geriatrics, Shenzhen Clinical Research Centre for Geriatrics, Department of Nuclear Medicine, Shenzhen People’s Hospital (The First Affiliated Hospital, Southern University of Science and Technology; The Second Clinical Medical College, Jinan University), Shenzhen, Guangdong 518020, P. R. China

^3^Department of Chemistry, The University of Chicago, Chicago 60615, IL, USA

^4^School of Chemistry and Chemical Engineering, Shanxi Datong University, Xing Yun Street, Pingcheng District, Datong, Shanxi Province 037009, P. R. China

^5^Harry Perkins Institute of Medical Research, QEII Medical Centre and Centre for Medical Research, The University of Western Australia, Nedlands, WA 6009, Australia

^6^Department of Visceral Surgery, Diakonissen Hospital, 67346 Speyer, Germany

^7^Department of Laboratory Medicine, Huadong Hospital, Fudan University, Shanghai 200040, P. R. China

^8^Department of Pathology, Nanfang Hospital, Southern Medical University, Guangzhou, 510515, P. R. China

^9^Department of Pathology & Guangdong Province Key Laboratory of Molecular Tumor Pathology, School of Basic Medical Sciences, Southern Medical University, Guangzhou, 510515, P. R. China

^10^School of Traditional Chinese Medicine and School of Pharmaceutical Sciences, Guangdong Provincial Key Laboratory of New Drug Screening, School of Pharmaceutical Sciences, Southern Medical University, Guangzhou 510515, Guangdong, P. R. China.

^11^State Key Laboratory for Quality Ensurance and Sustainable Use of Dao-di Herbs, Artemisinin Research Center, Institute of Chinese Materia Medica, China Academy of Chinese Medical Sciences, Beijing 100700, P. R. China.

^12^State Key Laboratory of Antiviral Drugs, School of Pharmacy, Henan University, Kaifeng 475004, P. R. China.

**Content**

1. **Supplementary Figures S1-S31**
2. **Supplementary materials and methods**
3. **The primary antibodies used in this study**
4. **Supplementary Tables S1-S3**

**Supplementary Figures**


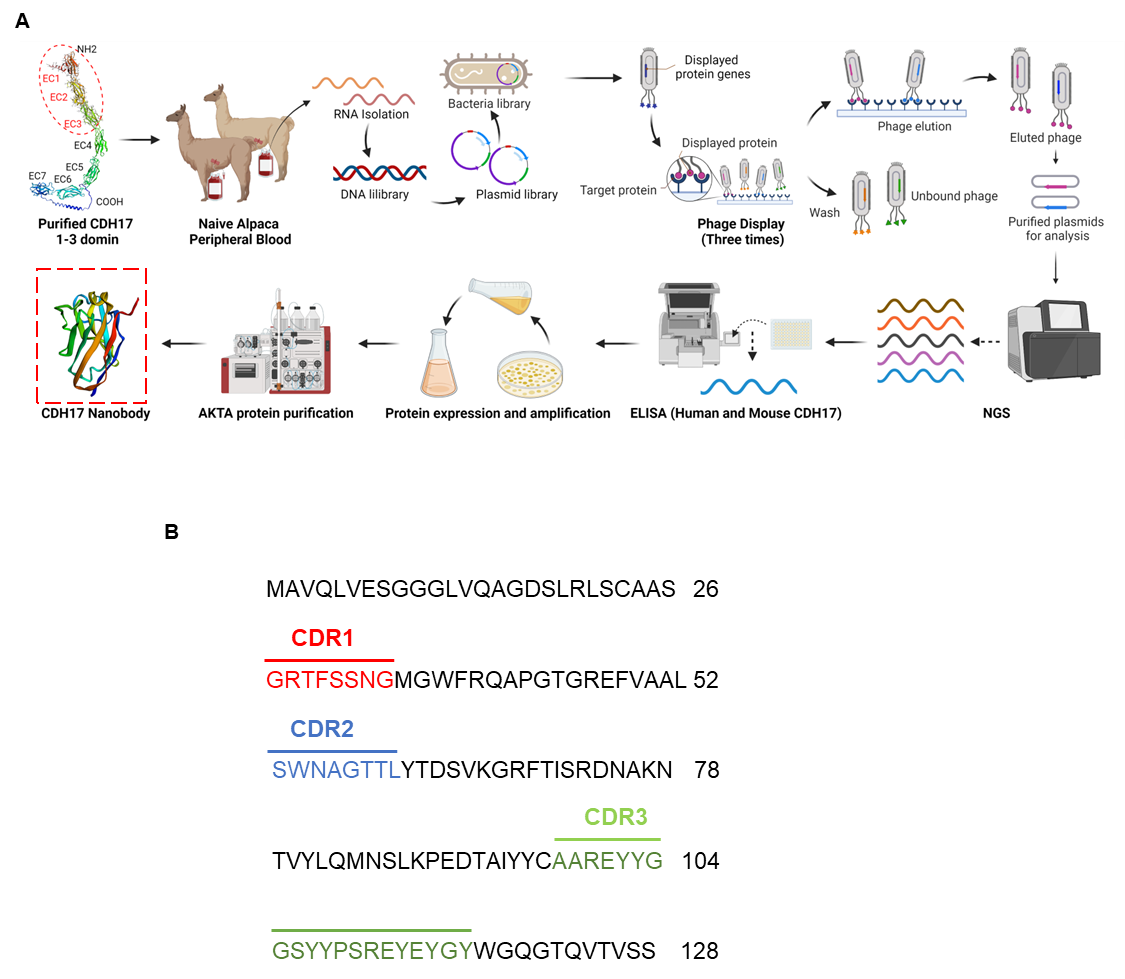


**Figure S1. CDH17 nanobody screening**

**A,** Screening procedure of CDH17 nanobody by phage display combined with deep sequencing. **B,** The sequence of CDH17 Nb289.


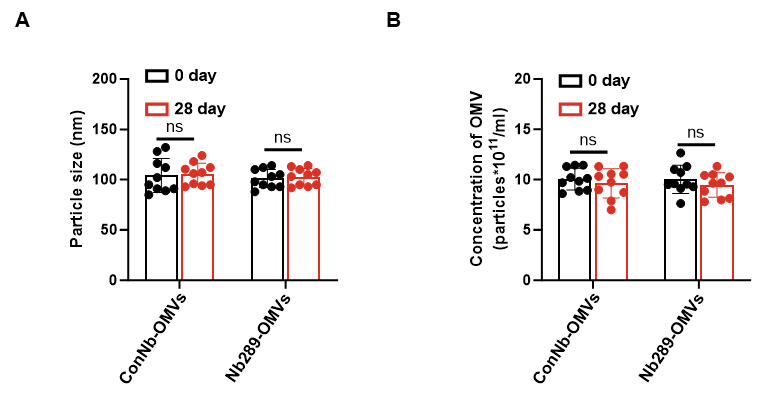


**Figure S2.** **Stability determination of engineered OMVs.**

**A,** The average particle size distribution of engineered OMVs incubated in PBS for 28 days at 4 degrees, determined by NTA (*n* = 10). **B**, Concentration determination of engineered OMVs incubated in PBS for 28 days, determined by NTA (*n* = 10). Results are presented as mean ± SD. Statistical significance was calculated using one-way ANOVA with Tukey’s post-test. ns indicates no significance.


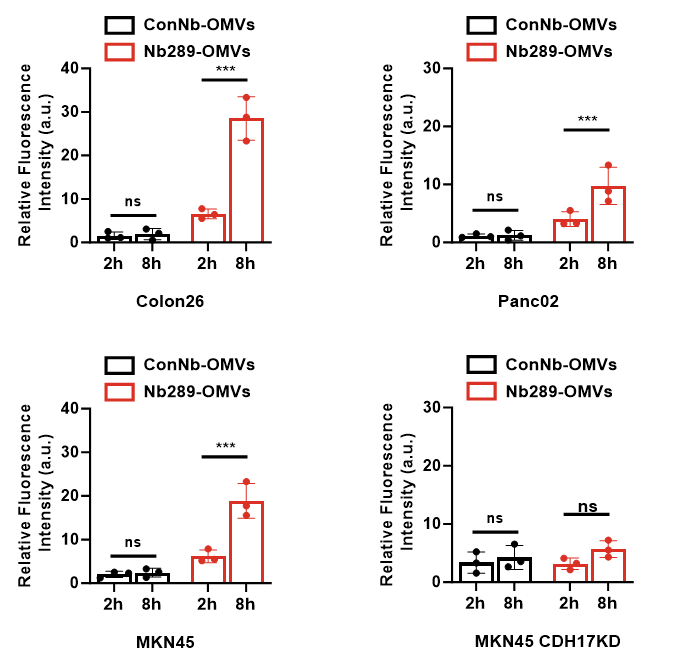


**Figure S3. Internalization efficiency of engineered OMVs.**

Quantification for internalization of PKH67-labeled OMVs (green) in **Figure 2C** (*n* = 3). The data are presented as mean ± SD. Statistical significance was calculated using unpaired student’s t-test. ****P* < 0.001, and ns means no significance.


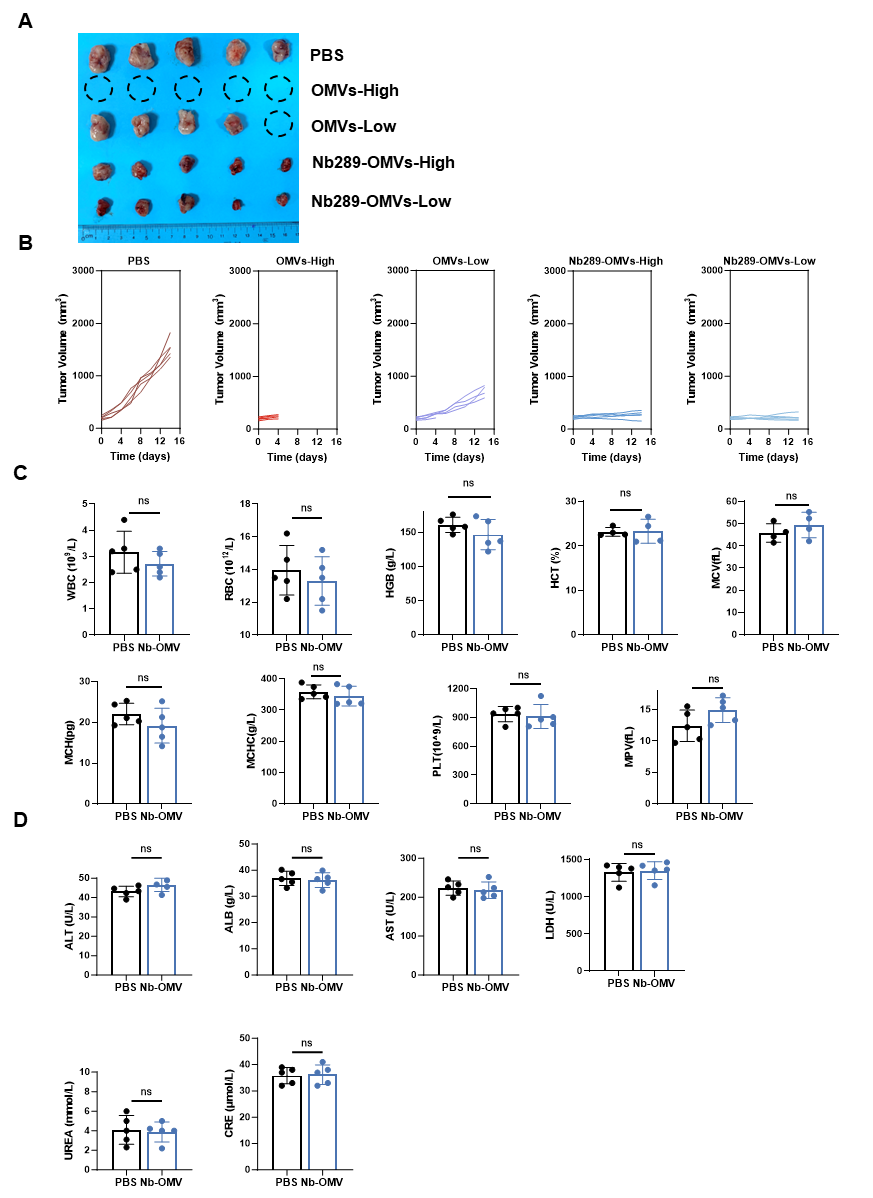


**Figure S4. The antitumor performance and safety assessment of nonengineered OMVs and Nb289-OMVs in Colon26 tumor-bearing mice.**

**A,** The tumor appearance at the end of the experiment (*n* = 5) after treatment with OMVs. **B,** Individual tumor growth curves for different groups (*n* = 5). **C,** Analysis of blood cell count for tumor-bearing mice receiving PBS and the low dose of Nb289-OMVs (*n* = 5). **D,** Analysis of serum biochemistry for liver and kidney functions (*n* = 5). The date are presented as mean ± SD. Statistical significance was calculated using two-tailed unpaired t-test analysis. ns means no significance. WBC, White blood cells; RBC, Red blood cells; HGB, Hemoglobin; HCT, Hematocrit; MCV, Mean corpuscular volume; MCH, Mean corpuscular hemoglobin; MCHC, Mean corpuscular hemoglobin concentration; PLT, Platelets; MPV, Mean platelet volume; ALT, Alanine aminotransferase; ALB. Albumin; AST, aspartate aminotransferase; LDH, Lactate Dehydrogenase; CRE, Creatinine.


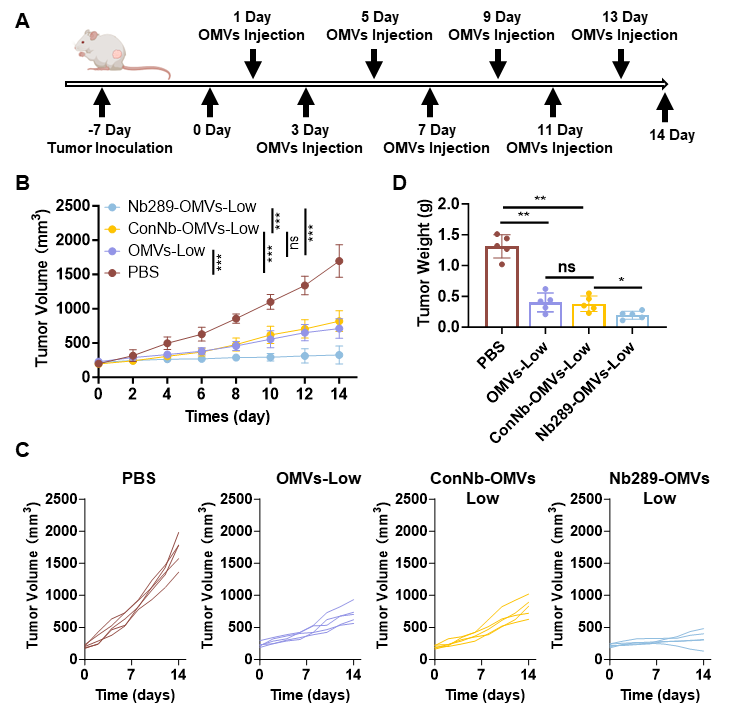


**Figure S5. The antitumor performance of OMVs, ConNb-OMVs and Nb289-OMVs in Colon26 tumor-bearing mice.**

**A**, Schematic representation of the treatment schedule with PBS (200 µL), Nb289-OMVs-Low (1×10^11^ particles/injection), ConNb-OMVs-Low (1×10^11^ particles/injection), and OMVs-Low (1×10^11^ particles/injection). **B**, Tumor growth curves under various treatments (*n*=5). **C**, Individual tumor growth curves during the treatments (*n*=5). **D,** Tumor weights after the different treatments (*n*=5). The date are presented as mean ± SD. Statistical significance was calculated using two-way ANOVA (B) or one-way ANOVA with Tukey’s post-test (D). ns indicates no significant difference. **P*<0.05, ***P*<0.01, and ****P*<0.001.


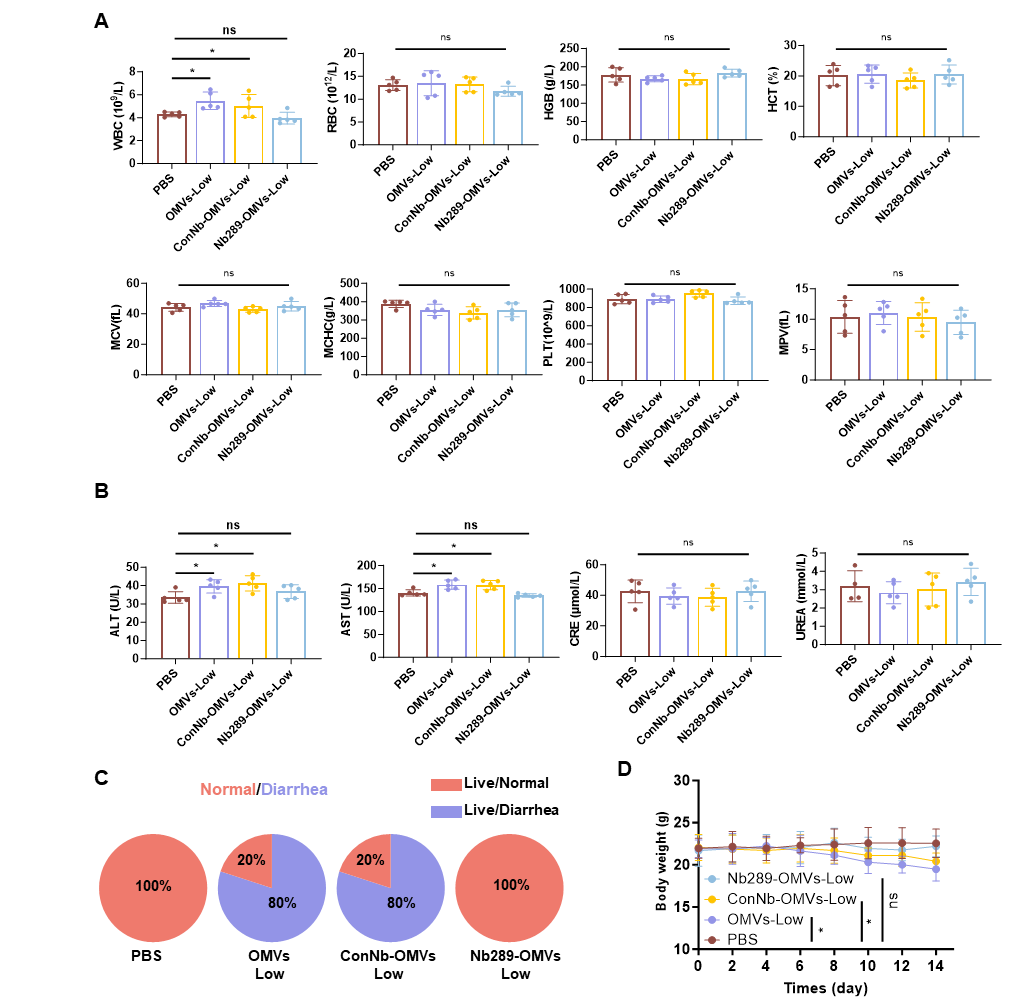


**Figure S6. The safety assessment of OMVs, ConNb-OMVs and Nb289-OMVs in Colon26 tumor-bearing mice.**

**A,** Parameter analysis for serum blood routine index (*n* = 5). **B,** Parameter analysis for serum blood biochemistry test (*n* = 5). **C**, Incidence of diarrhea in OMV-treated mice (*n*=5). **D**, Body weight changes during the treatment period (*n*=5). The date are presented as mean ± SD. Statistical significance was calculated using one-way ANOVA with Tukey’s post-test (A, B) and two-way ANOVA (D). ns indicates no significant difference. **P*<0.05, ***P*<0.01, and ****P*<0.001. WBC, White blood cells; RBC, Red blood cells; HGB, Hemoglobin; HCT, Hematocrit; ALT, Alanine aminotransferase; AST, aspartate aminotransferase; CRE, Creatinine.


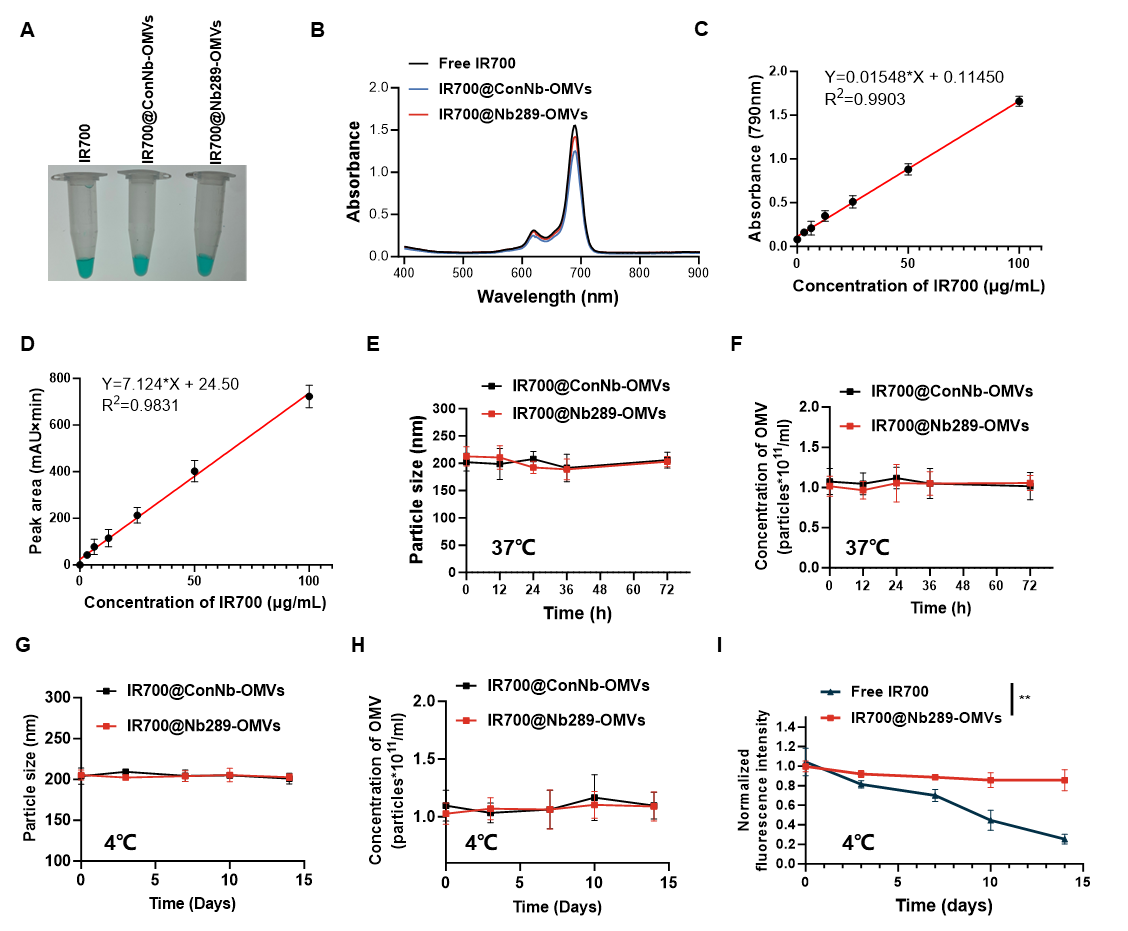


**Figure S7. The characterization of IR700-loaded OMVs.**

**A,** The general appearance of Free IR700, IR700@Con Nb-OMVs and IR700@Nb289-OMVs. **B,** UV–vis absorption spectra for Free IR700, IR700@Con Nb-OMVs and IR700@Nb289-OMV (*n* = 1). **C,** IR700 standard curve based on absorbance and unknown concentrations (*n* = 3). **D,** IR700 standard curve based on HPLC peak area and concentrations (n = 3). **E,** The particle sizes of IR700-loaded OMVs detected by NTA at 37°C within 72 h (n = 3). **F,** The concentration analysis of IR700-loaded OMVs detected by NTA at 37°C within 72 h (*n* = 3). **G,** The examination for particle sizes of IR700@Con Nb-OMVs and IR700@Nb-OMVs after incubation in PBS at 4 °C within 14 days (*n* = 3). **H,** The concentrations of IR700@Con Nb-OMVs and IR700@Nb289-OMVs after incubation in PBS at 4 °C within 14 days (*n* = 3). **I,** Fluorescence intensity changes for free IR700 and IR700@Nb289-OMVs after incubation in PBS at 4 °C with 14 days (*n* = 3). The data are presented as mean ± SD. Statistical significance was calculated using two-way ANOVA with Tukey’s post-test. ***P*< 0.01.


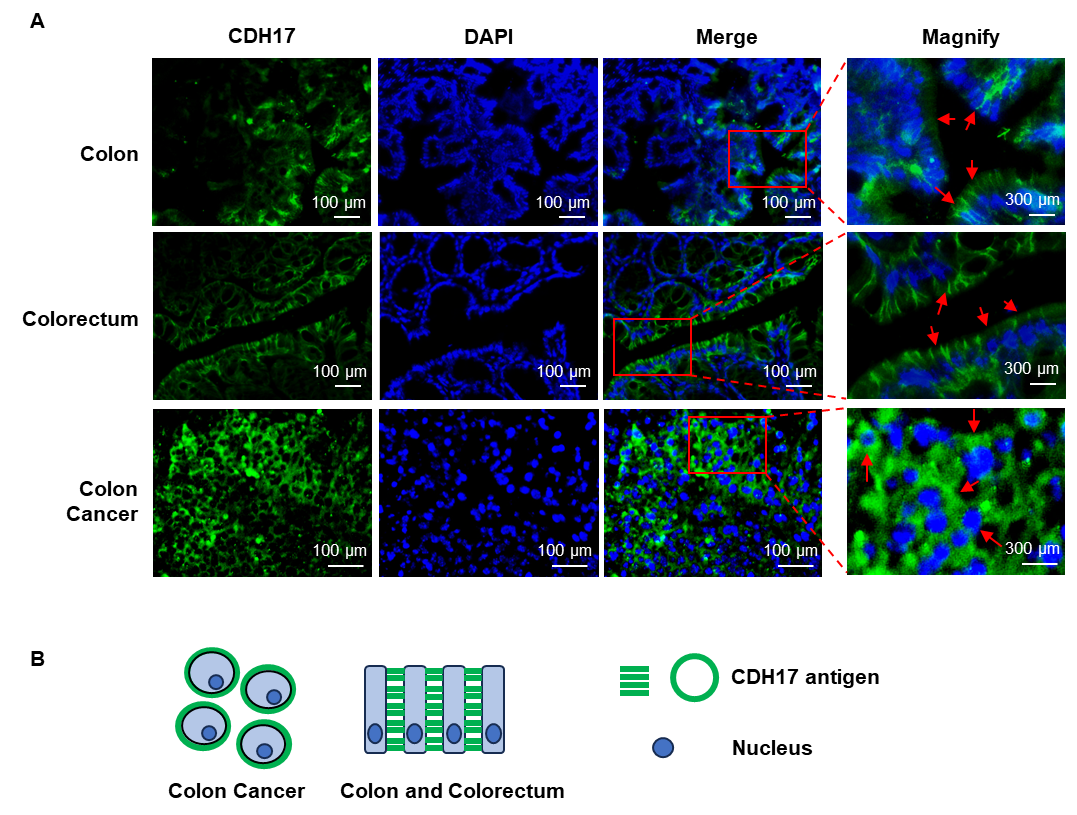


**Figure S8. Immunofluorescence analysis of CDH17 expression in normal intestinal epithelial cells and cancer cells.**

A, Immunofluorescence staining of CDH17 in normal mouse colon tissues, colorectal tissues and colon cancer tissues. The red arrows marked the luminal or basal surface of the normal tissues in which there is no CDH17 expression; but the expression of CDH17 surrounded the whole tumor cells in cancer tissues. Scale bars, 100 μm (left), 300 μm (right). B, Schematic representation depicting the expression localization of CDH17 on the colonic epithelial cells, colorectal epithelial cells, and tumor cells in Colon26-induced tumor tissues.


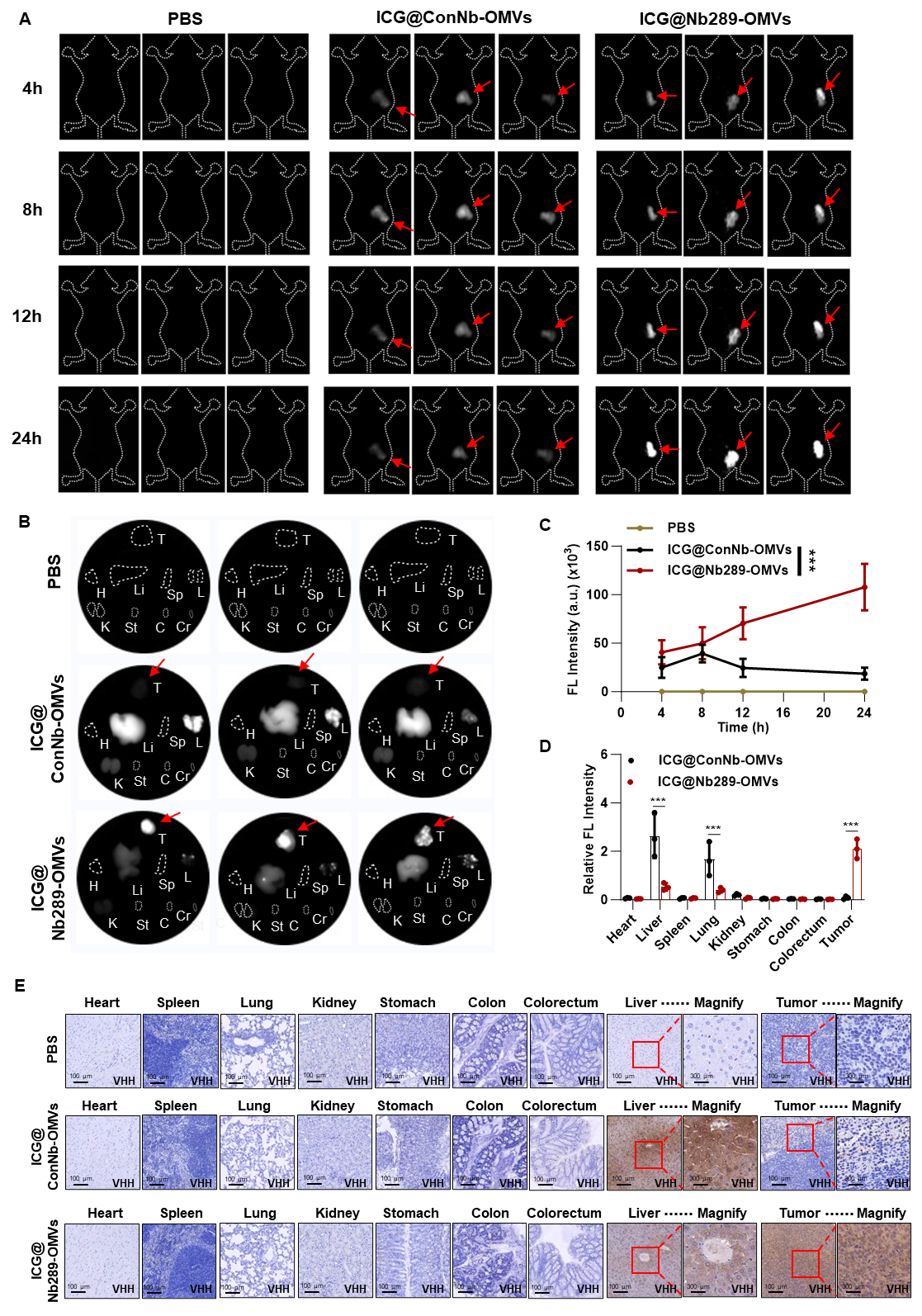


**Figure S9. *In vivo* NIR-II imaging of subcutaneous colon cancer using Nb289-OMVs loaded with ICG.**

**A,** Following intravenous injection of PBS (200 μl), ICG@ConNb-OMVs (100 μg/ml, 200 μl), or ICG@Nb289-OMVs (100 μg/ml, 200 μl) into subcutaneous Colon26 tumor-bearing mice, *in vivo* NIR-II imaging was performed at various time points (4 h, 8 h, 12 h, and 24 h) (*n* = 3). **B,** At 24 h post-injection, the main organs (heart, liver, spleen, lung, kidney, stomach, colon, and colorectum) and tumor tissues were dissected, and ex vivo NIR-II imaging was performed (*n* = 3). **C,** Quantitative tumor fluorescence analysis of NIR-II imaging in subcutaneous Colon26 tumor-bearing mice. **D,** Quantitative fluorescence analysis of NIR-II imaging for major organs and tumor tissues dissected from subcutaneous Colon26 tumor-bearing mice. **E,** Immunohistochemical staining of nanobodies in major organs and tumor tissues using anti-VHH antibodies. Scale bars: 100 μm. The data are presented as mean ± SD. Statistical significance was calculated using two-way ANOVA (C) or one-way ANOVA (D) with Tukey’s post-test. **P*<0.05, ***P*<0.01, and ****P*<0.001.


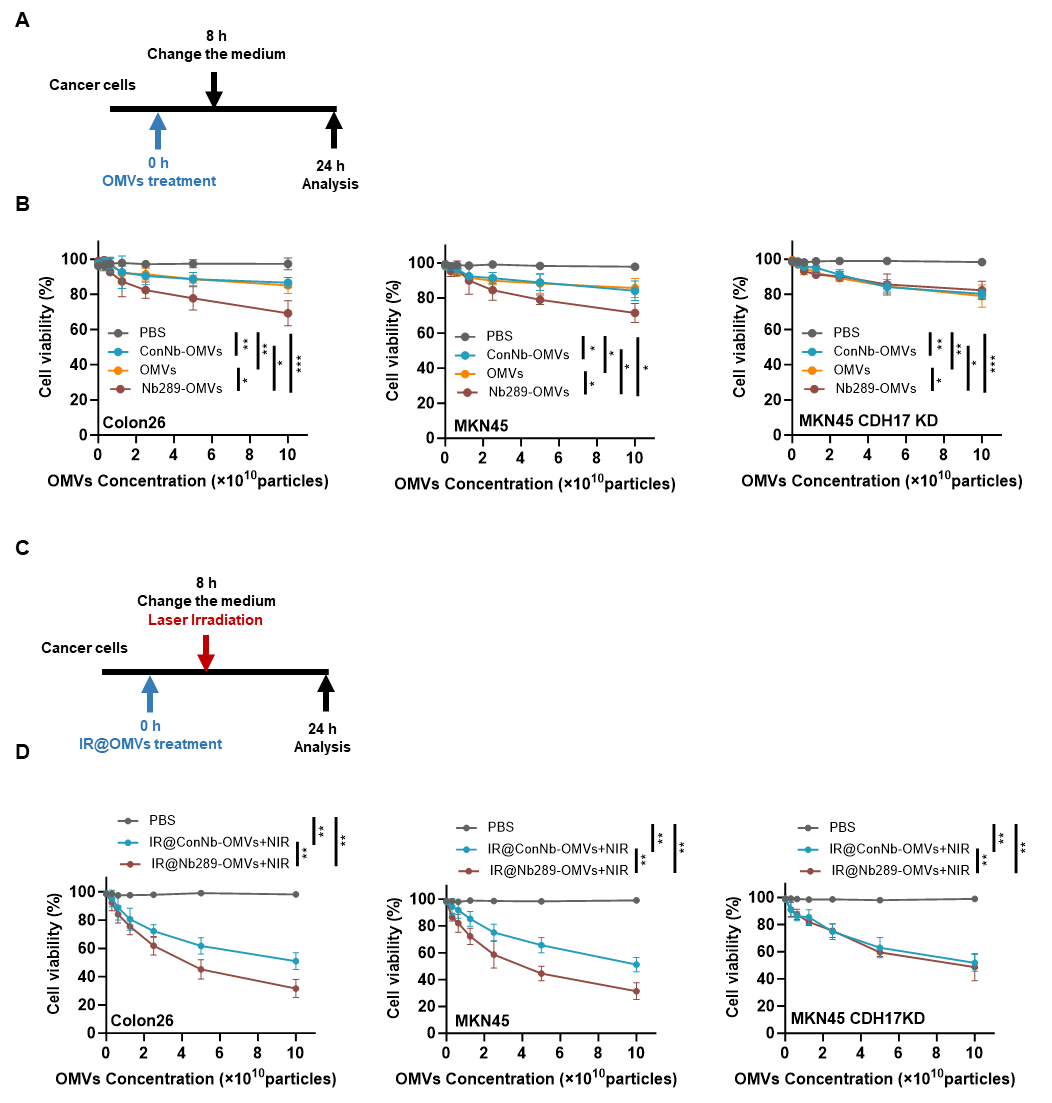


**Figure S10.** The *in vitro* antitumor performance of OMVs, ConNb-OMVs and Nb289-OMVs in cancer cells

**A**, Schematic of CCK-8 assay in Colon26, MKN45, and CDH17-knockdown MKN45 cells treated with PBS (200 µL), Nb289-OMVs, OMVs, or ConNb@OMVs (1×10¹⁰ particles/mL). B, Cell viability post-treatment (n=3). Cells were incubated with OMVs for 8 h, followed by media replacement. Viability was assessed 24 h later. C, Schematic of CCK-8 assay for cells treated with PBS, IR700@ConNb@OMVs (1×10¹⁰ particles/mL) + NIR (20 J/cm²), or IR700@Nb289-OMVs (1×10¹⁰ particles/mL) + NIR (20 J/cm²). **D**, Cell viability after photoimmunotherapy (n=3). Cells were incubated with OMVs for 8 h, followed by NIR irradiation and media replacement. Viability was measured 24 h post-treatment. The data are presented as mean ± SD. Statistical significance was calculated using two-way ANOVA (B, D). **P*<0.05, ***P*<0.01, and ****P*<0.001.


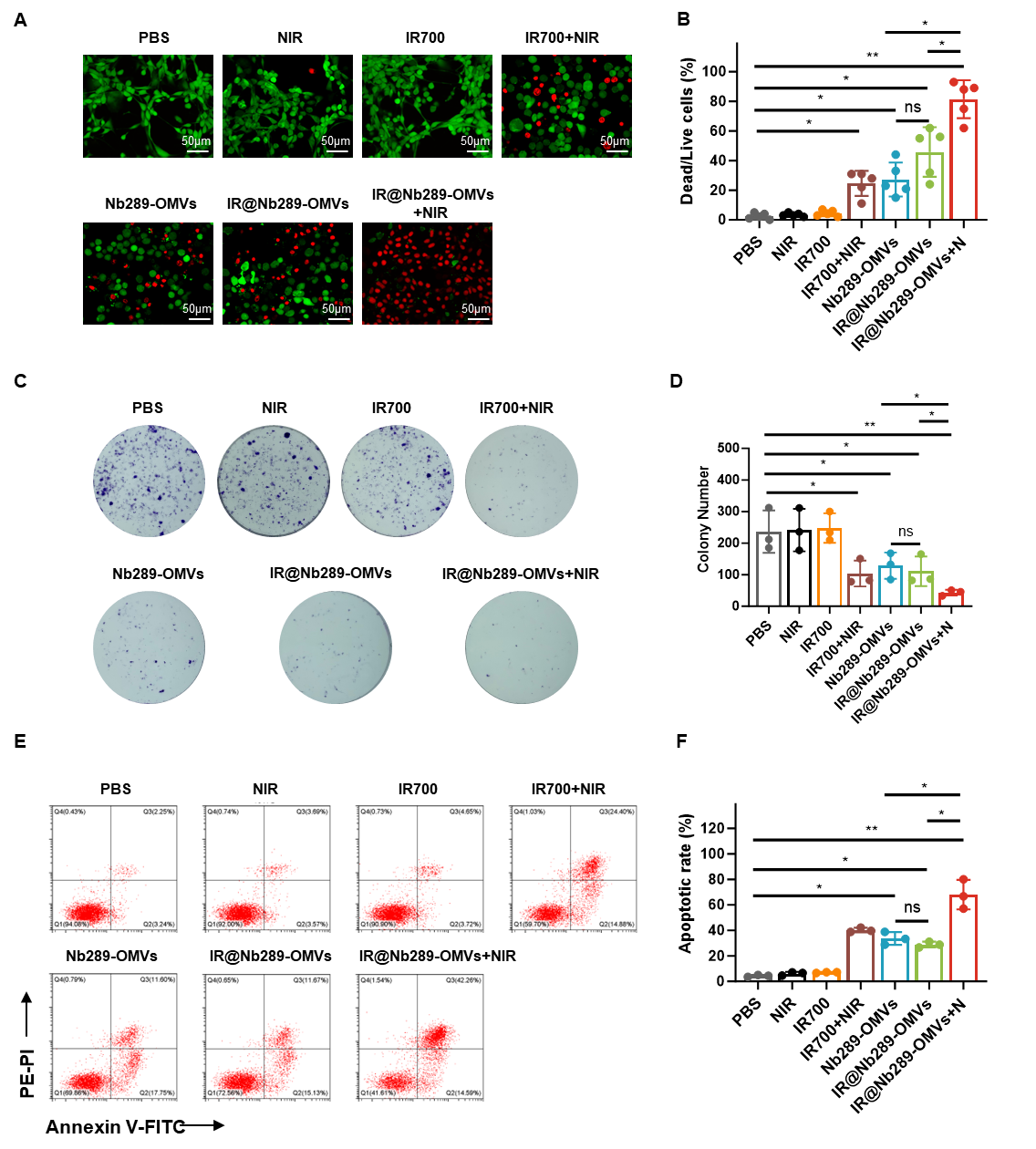


**Figure S11. The *in vitro* inhibitory effects of IR700@Nb289-OMVs on CDH17-positive Colon26 cancer cells**

**A,** LIVE/DEAD cell staining for Colon26 cells treated with different regimens (*n* = 3). Green staining indicates live cells and red for dead cells. **B,** Statistical results of LIVE/DEAD cell staining. **C,** Colony formation assay for Colon26 cells after various treatments (*n* = 3). **D,** Statistical results of Cell colony formation. **E,** Detection of apoptotic cells by flow cytometry in Colon26 cells receiving different treatments (*n* = 3). **F,** Statistical results of flow cytometry. Statistical significance was calculated using one-way ANOVA with Tukey’s post-test. The data are presented as mean ± SD. **P* < 0.05, ***P* < 0.01, ****P* < 0.001.


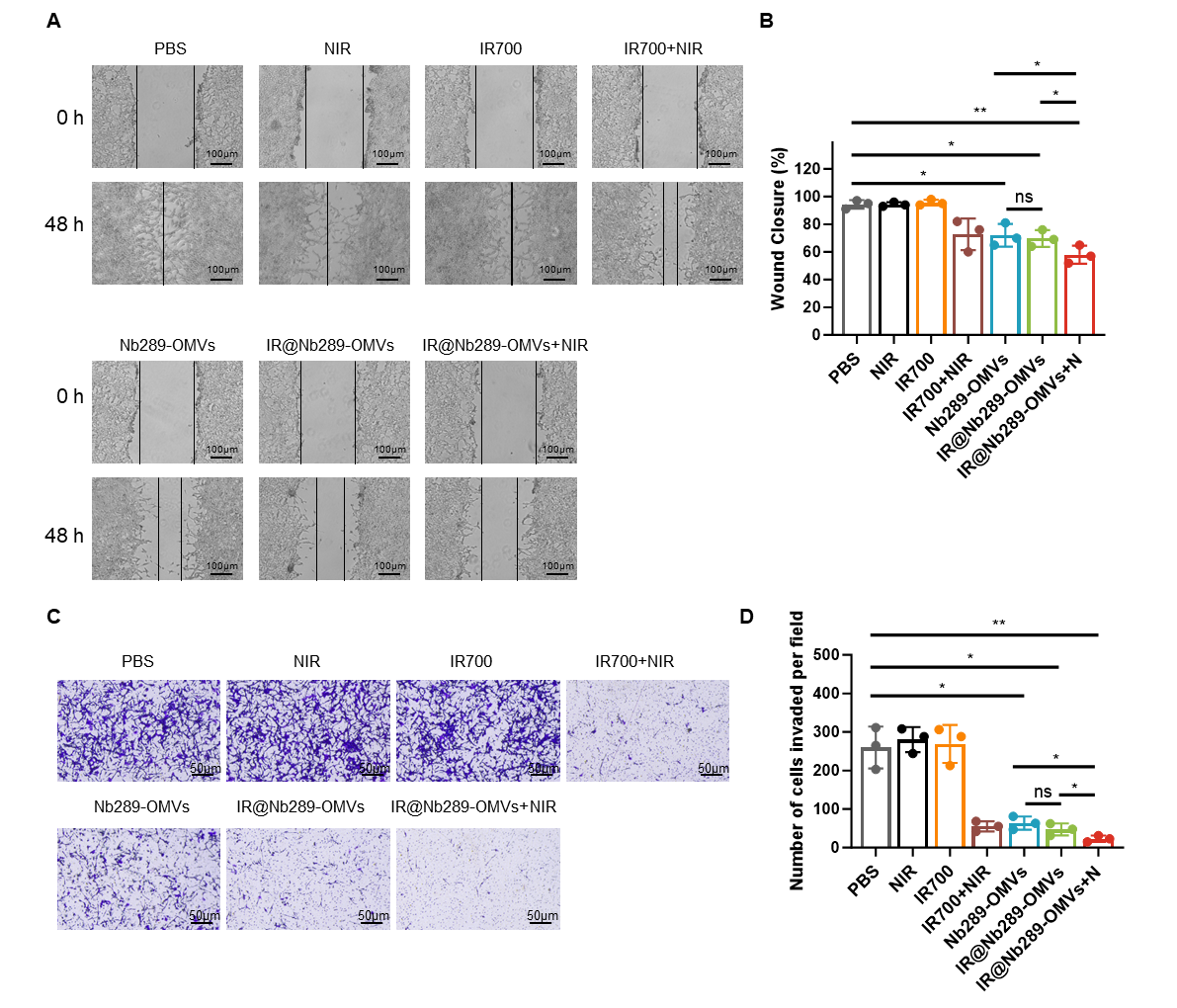


**Figure S12. IR700@Nb289-OMVs suppress the migration and invasion of tumor cells *in vitro*.**

**A,** Cell migration evaluated using wound-healing migration assay in Colon26 cancer cells receiving various treatments (*n* = 3). Scale bars, 100 μm. **B,** Statistical results of wound-healing assay. **C,** Cell invasion ability examined using transwell invasion assay under various treatments (*n* = 3). Scale bars, 50 μm. **D,** Statistical results of transwell invasion assay. The data are presented as mean ± SD. Statistical significance was calculated using one-way ANOVA with Tukey’s post-test. **P* < 0.05, ***P* < 0.01, ****P* < 0.001.


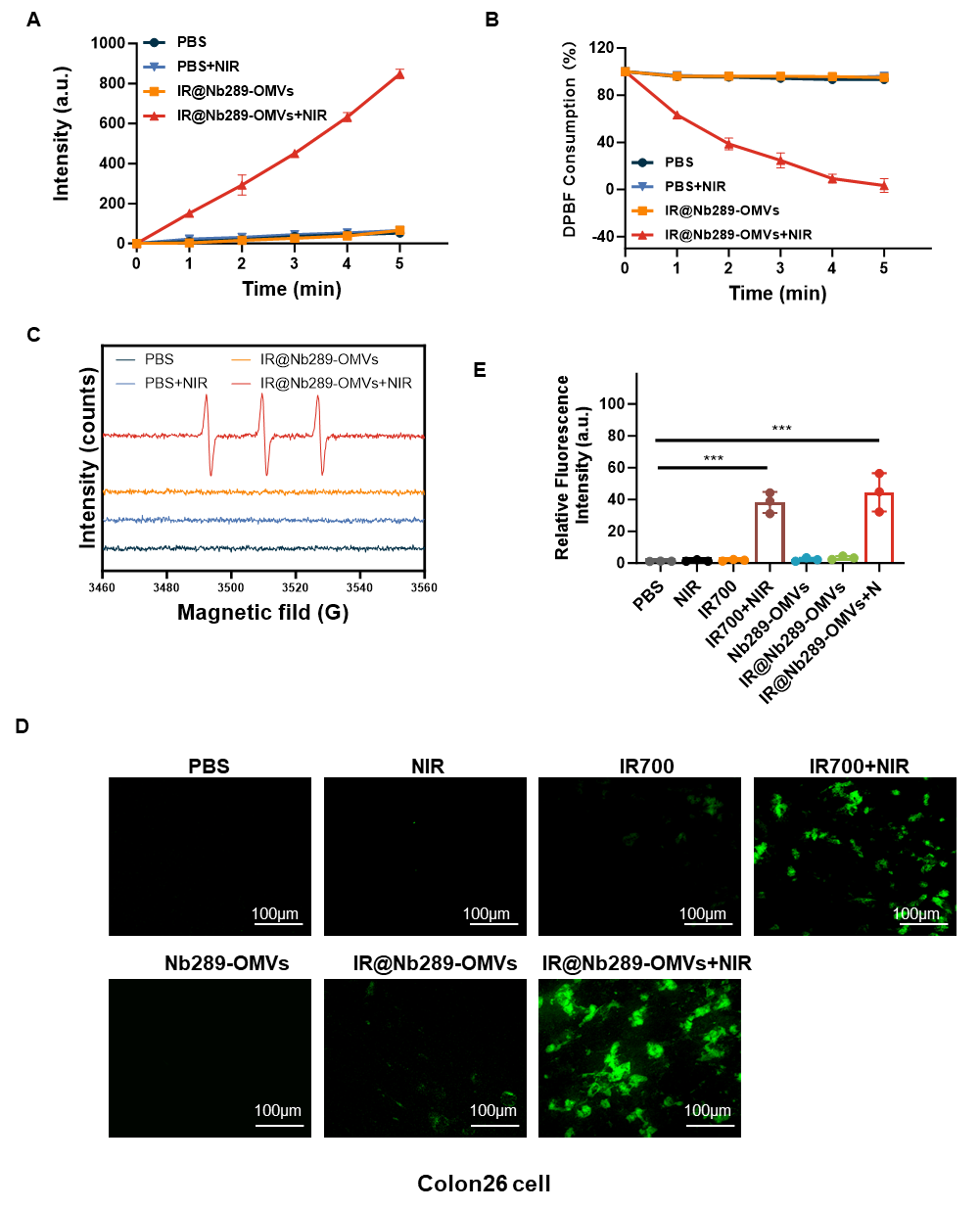


**Figure S13. Detection of intracellular ROS for IR700-modified OMVs**

**A,** Fluorescence intensity of DCFH-DA in solutions receiving different treatments (n = 3). **B,** DPBF consumption in solutions receiving different treatments (n = 3). **C,** ESR spectra of different conditions for the detection of singlet oxygen. **D,** Fluorescence images of ROS in Colon26 cells detected by DCFH-DA staining after different treatments (n = 3). **E,** Statistical results of **Figure D**. Scale bars: 100 µm. The data are presented as mean ± SD. Statistical significance was calculated using one-way ANOVA with Tukey’s post-test. **P* < 0.05, ***P* < 0.01, ****P* < 0.001.

**
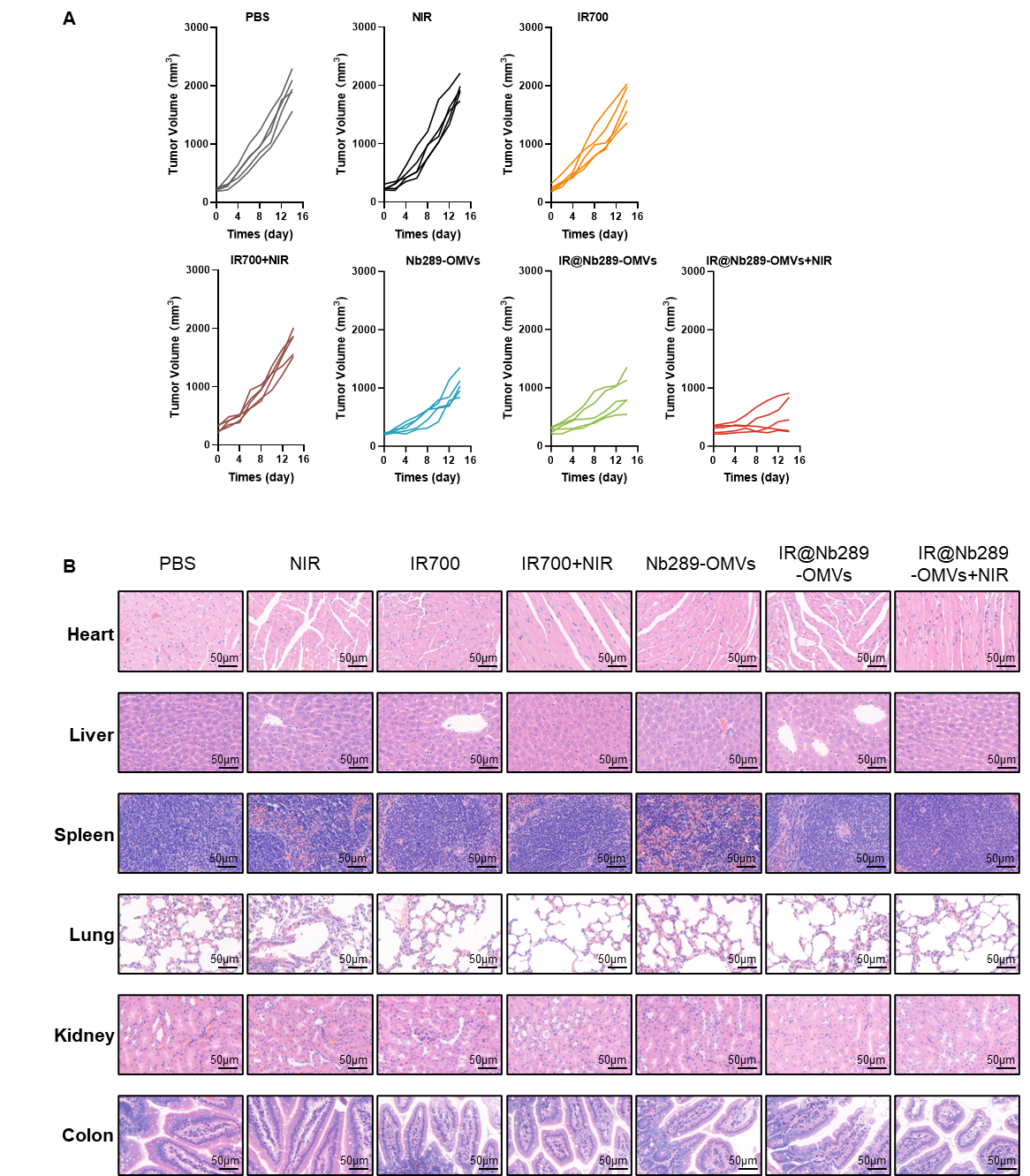
**

**Figure S14. IR700@Nb289-OMVs effectively suppress tumor growth with the good biosafety.**

**A,** Individual tumor growth curves of Colon26 tumors from different groups related to Figure 3H. **B,** H&E staining for major organs (heart, liver, spleen, lung, kidney and colon) from treated mice in (A) (*n* = 5).


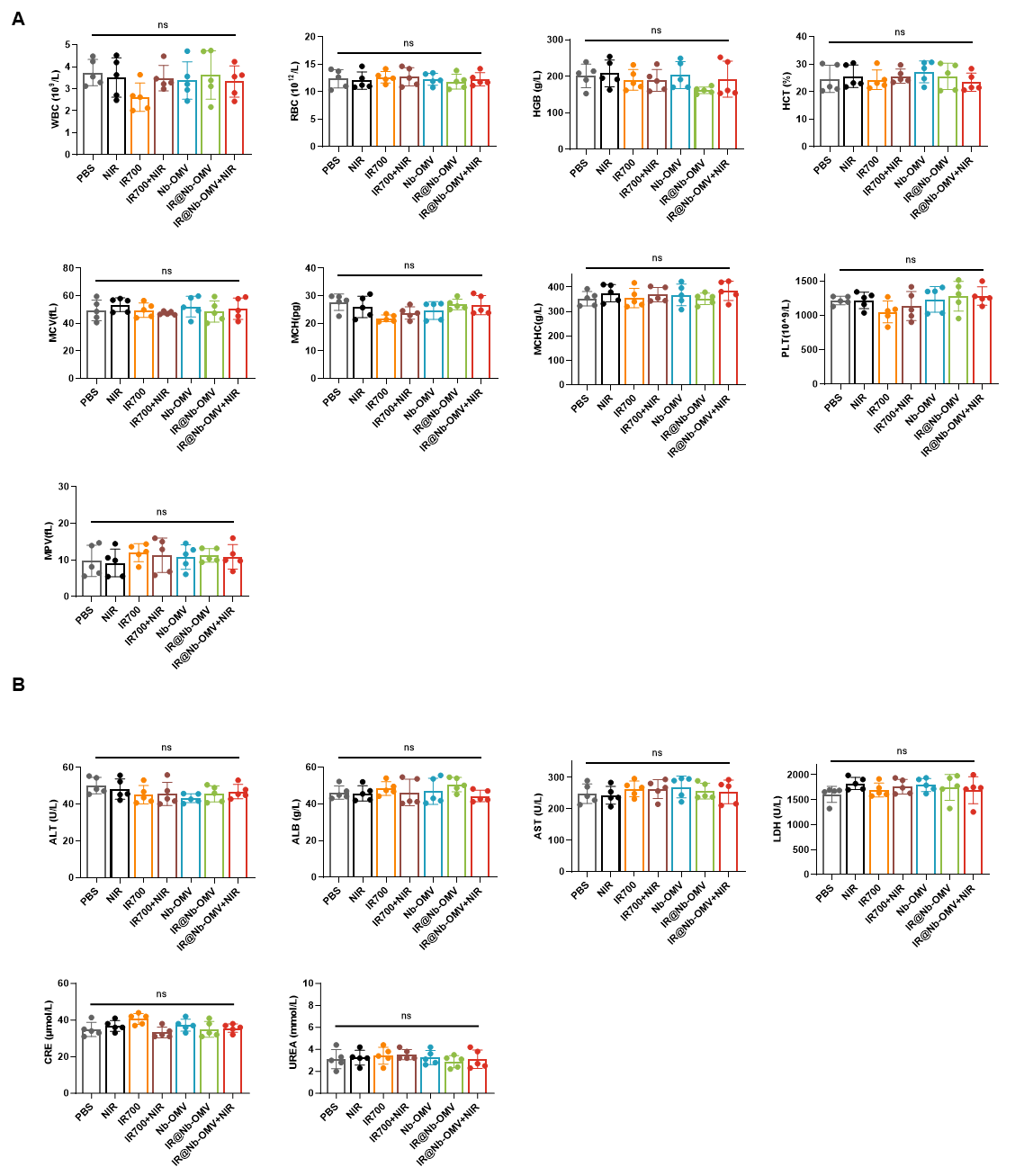


**Figure S15. The analysis for blood cell counts and biochemistry after the treatment with IR700@Nb289-OMVs.**

**A,** Parameter analysis for serum blood routine index (*n* = 5). **B,** Parameter analysis for serum blood biochemistry test (*n* = 5). The data are presented as mean ± SD. Statistical significance was calculated using one-way ANOVA with Tukey’s post-test. ns indicates no significant difference. WBC, White blood cells; RBC, Red blood cells; HGB, Hemoglobin; HCT, Hematocrit; MCV, Mean corpuscular volume; MCH, Mean corpuscular hemoglobin; MCHC, Mean corpuscular hemoglobin concentration; PLT, Platelets; MPV, Mean platelet volume; ALT, Alanine aminotransferase; ALB. Albumin; AST, aspartate aminotransferase; LDH, Lactate Dehydrogenase; CRE, Creatinine.


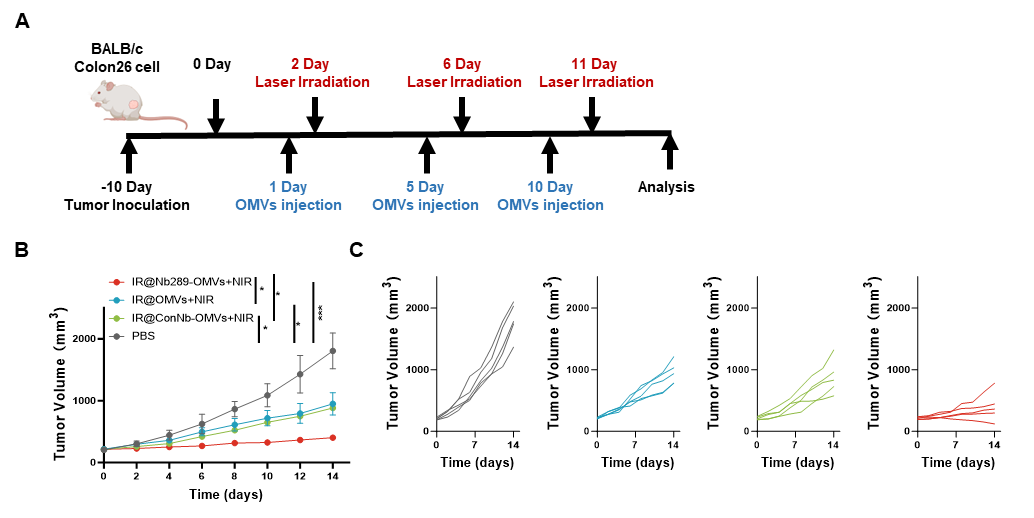


**FigureS16. The antitumor performance of various IR700-loaded OMVs in Colon26 tumor-bearing mice.**

**A,** Schematic representation of the treatment schedule with PBS (200 µL), IR700@Nb289-OMVs (1×10^11^ particles/injection) + NIR (50 J/cm^2^), R700@ConNb-OMVs (1×10^11^ particles/injection) + NIR (50 J/cm^2^), and IR700@ OMVs (1×10^11^ particles/injection) + NIR (50 J/cm^2^). **B,** Tumor growth curves under various treatments regiments with the PD-1 antibody (*n*=5). **C,** Individual tumor growth curves at the end of the experiment (*n*=5). The results are presented as mean ± SD. Statistical significance was calculated using two-way ANOVA with Tukey’s post-test. **P* < 0.05, ***P* < 0.01, ****P* < 0.001.

**
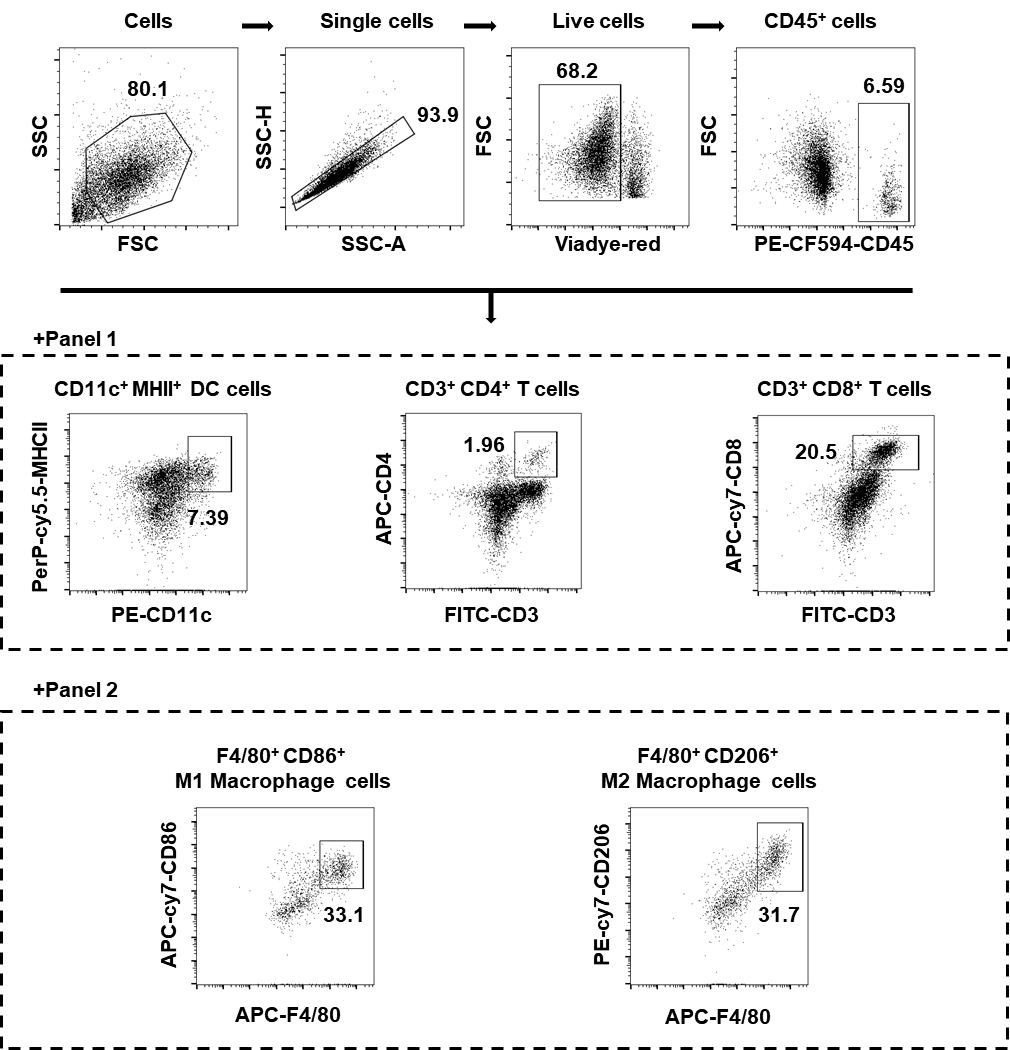
**

**Figure S17. The gating strategy for TME analysis**

The gating strategy for T cells, DCs, macrophages in tumors.

**
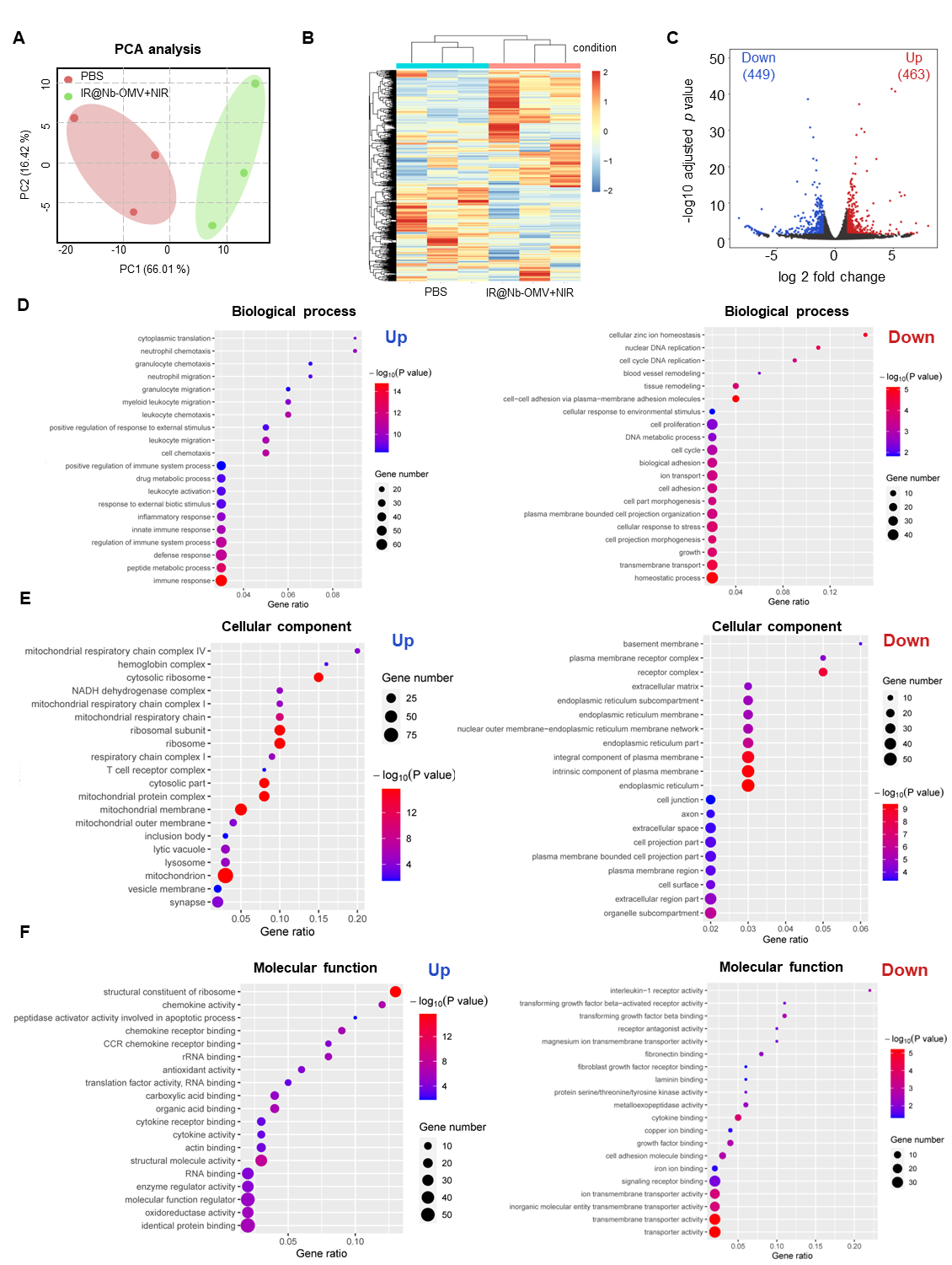
**

**Figure S18. RNA-seq analysis from tumors treated with** **IR700@Nb289-OMVs plus NIR.**

**A,** Principal component analysis (PCA) of RNA-Seq data (*n* = 3). **B, C,** Heat map and Volcano plot for differential gene expression between PBS and IR700@Nb289-OMVs plus NIR (*n*=3). **D-F,** Biological process (D), Cellular component (E) and Molecular function (F) GO categories enrichment analyses.


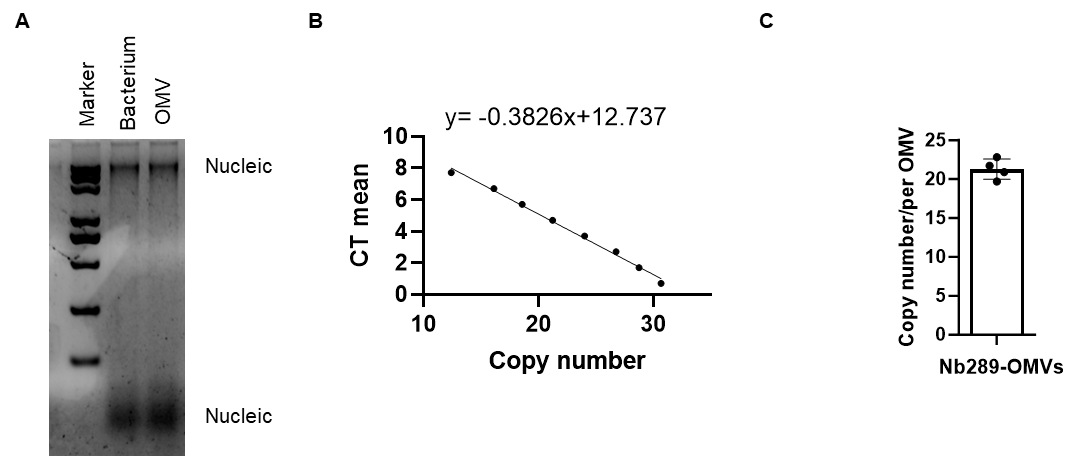


**Figure S19. The detection of nucleic acids in OMVs**

**A,** The agarose gel electrophoresis analysis for nucleic acids in the OMVs and supernatant from bacteria MG1655. **B,** Standard curve for DNA copy number established by qPCR with plasmids. **C,** Determination of copy number for 16S rDNA per Nb289-OMV (*n*=4). The results are presented as mean ± SD.


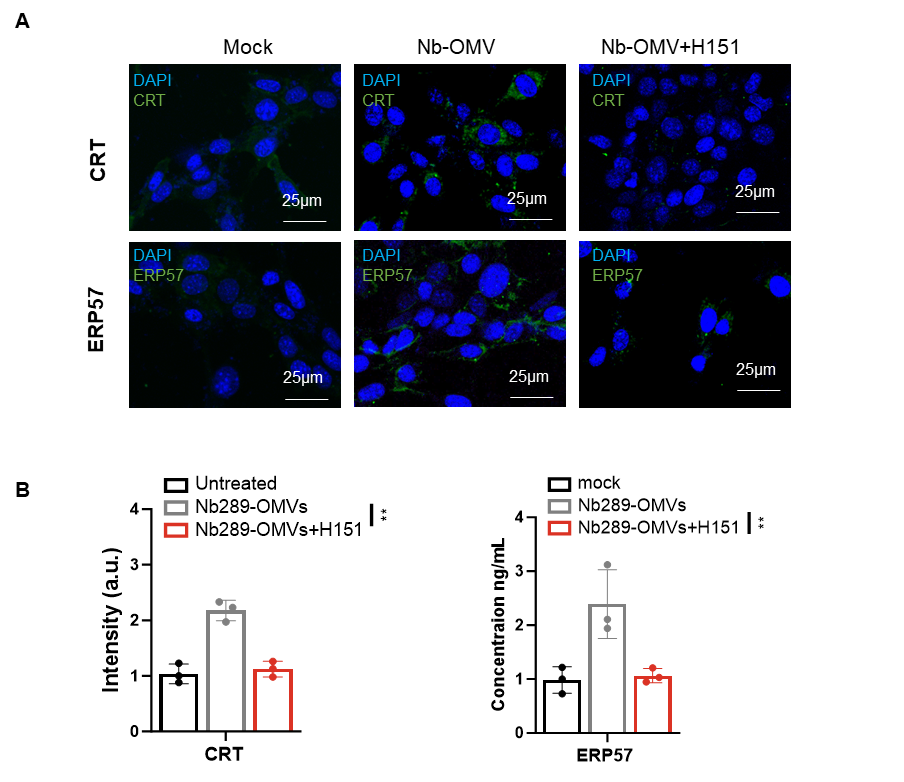


**Figure S20. STING activation by Nb289-OMVs induces ICD in cancer cells.**

**A,** Immunofluorescence detection of CRT and ERp57 surface expression levels in Colon26 cancer cells treated with Nb289-OMVs or combined with H151 (*n* = 3). **B**, Quantification of fluorescence intensity of CRT and ERp57 for (A). The results are presented as mean ± SD. Statistical significance was calculated using one-way ANOVA with Tukey’s post-test. ***P* < 0.01.


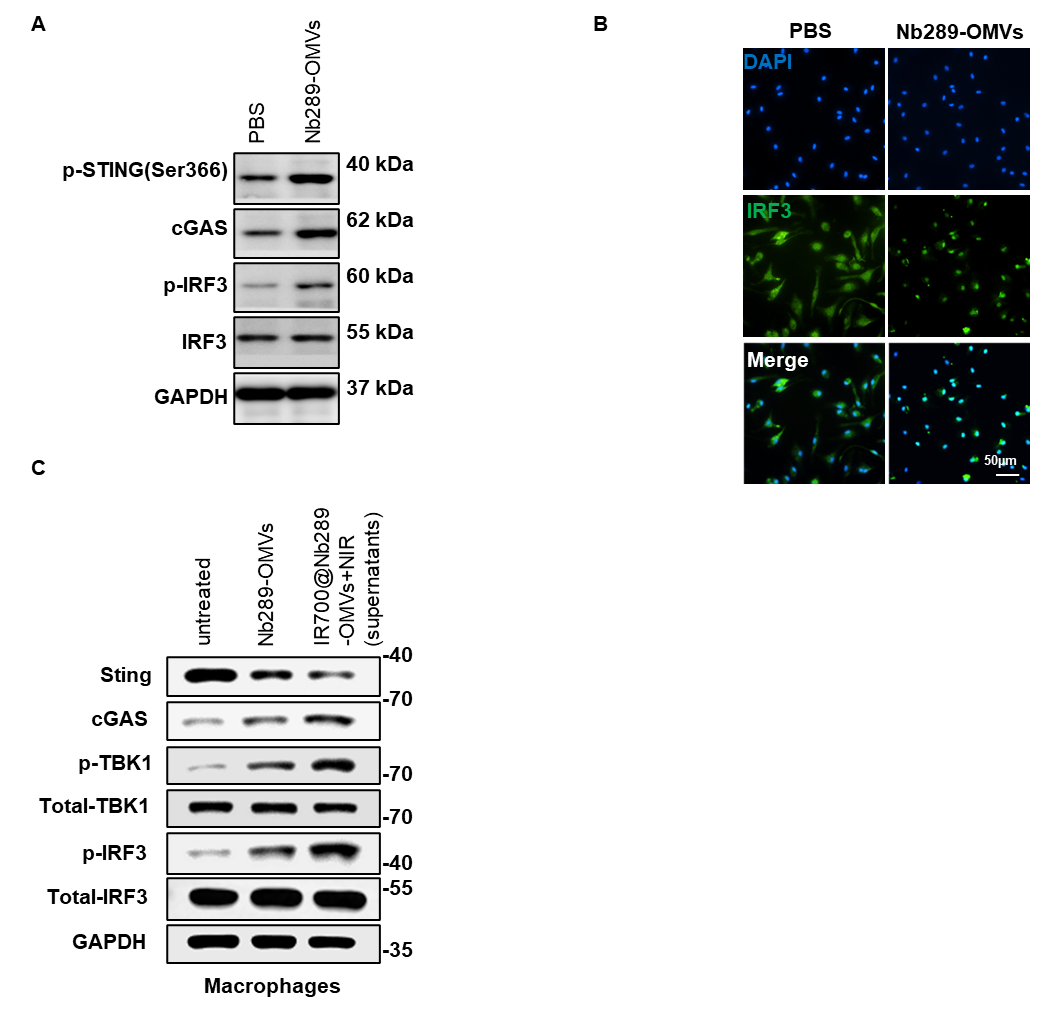


**Figure S21. Nb289-OMVs induce STING activation in macrophages.**

**A,** The examination of the activation of STING pathway in macrophages treated with Nb289-OMVs using western blotting (*n* = 3). **B,** IRF3 immunofluorescence staining in macrophages treated with Nb289-OMVs (n = 3). **C,** The examination of the STING pathway in macrophages treated with the supernatants from Colon26 cells. The Colon26 cells were first pretreated with IR700@Nb289-OMVs overnight. NIR (20 J/cm^2^) irradiation was applied for IR700@Nb289-OMVs-treated cells, and the cells were incubated overnight. The supernatants were then collected to treat the isolated primary macrophages for 4 hours to detect the STING pathway activation, and Nb289-OMVs were used as a control to directly incubate with macrophages for 4 hours (*n* = 3).


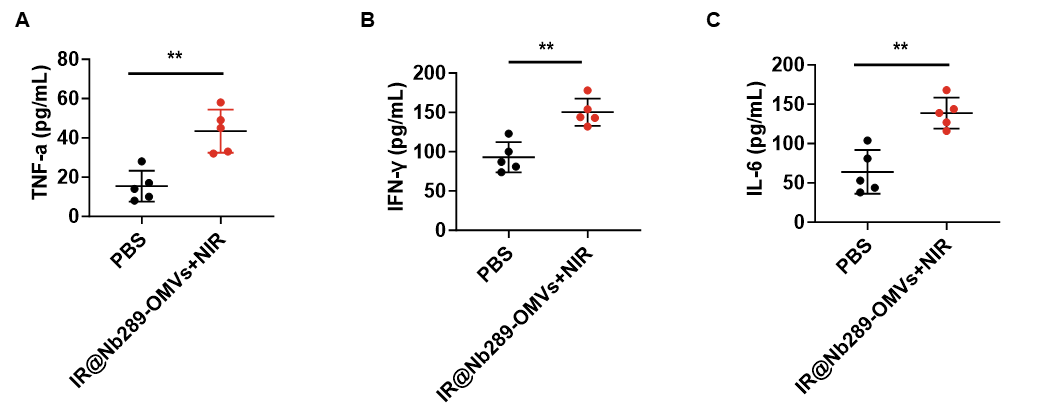


**Figure S22. The analysis of** **systemic proinflammatory cytokines in the blood of tumor-bearing mice treated with IR700@Nb289-OMVs plus NIR.**

**A-C,** The examination of systemic proinflammatory cytokines TNF-α (A), IFN-γ (B) and IL-6 (C) by ELISA (*n* = 5). The results are presented as mean ± SD. Statistical significance was calculated using two-tailed unpaired t-test analysis. ***P* < 0.01.


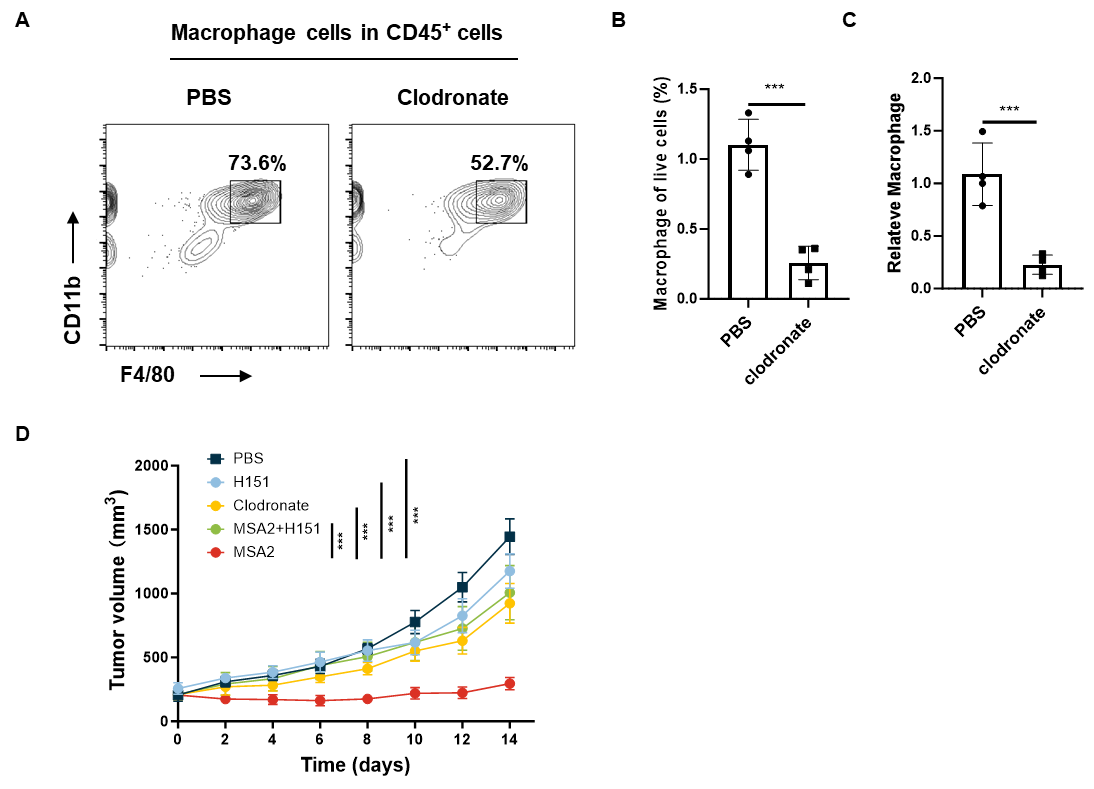


**Figure S23. Colon26 tumors receiving macrophage depletion and STING inhibition *in vivo***

**A,** Detection of macrophage depletion efficiency by flow cytometry in tumors (*n* = 4). **B, C,** Statistical results of macrophage depletion (*n* = 4). **D,** Tumor growth curves of Colon26 tumors under various treatments (*n* = 5). The 5 groups were given PBS (200 µL), H151 (10 mg/kg, intraperitoneal injection for 7 days), clodronate (200 µL, 5 mg/mL, peritumoral injection lasted 7 days), MSA2 (200 µL, 50 mg/kg, one injection inside the tumor) and MSA2 (200 µL, 50 mg/kg, single subcutaneous injection) + H151 (10 mg/kg, intraperitoneal injection for 7 days), respectively**.** The results are presented as Mean ± SD. Statistical significance was calculated using two-tailed unpaired t-test analysis (B, C) and two-way ANOVA (D). **P* < 0.05, ***P* < 0.01, ****P* < 0.001. ns indicates no significant difference.


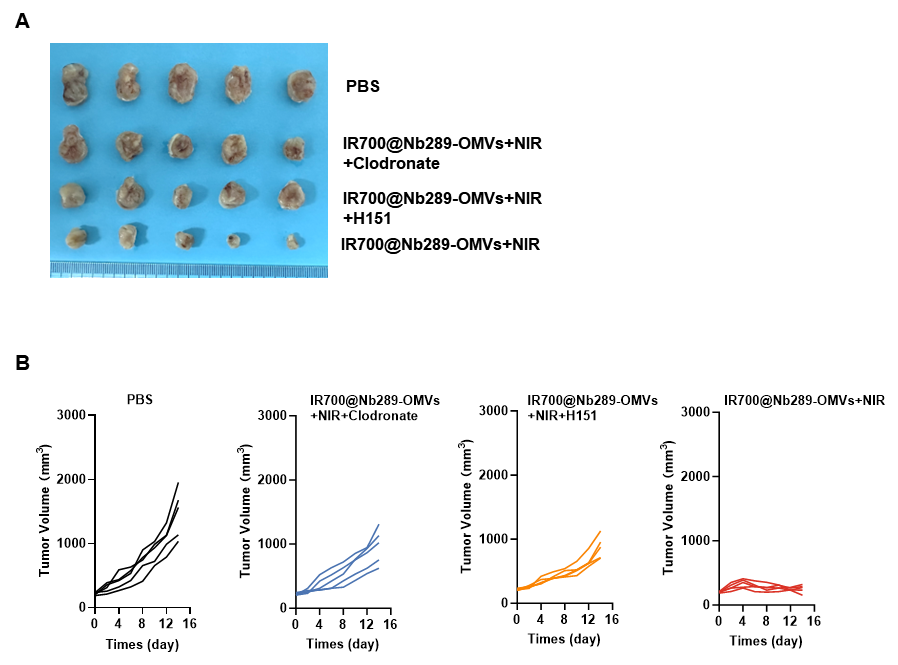


**Figure S24. The effect of macrophage depletion or STING inhibition on the therapeutic effect of IR700@Nb289-OMV-mediated photoimmunotherapy.**

**A,** Tumor images of subcutaneous Colon26 tumors received different treatments (*n* = 5) **B,** Individual tumor growth curve from mice with various treatments (*n* = 5).

**
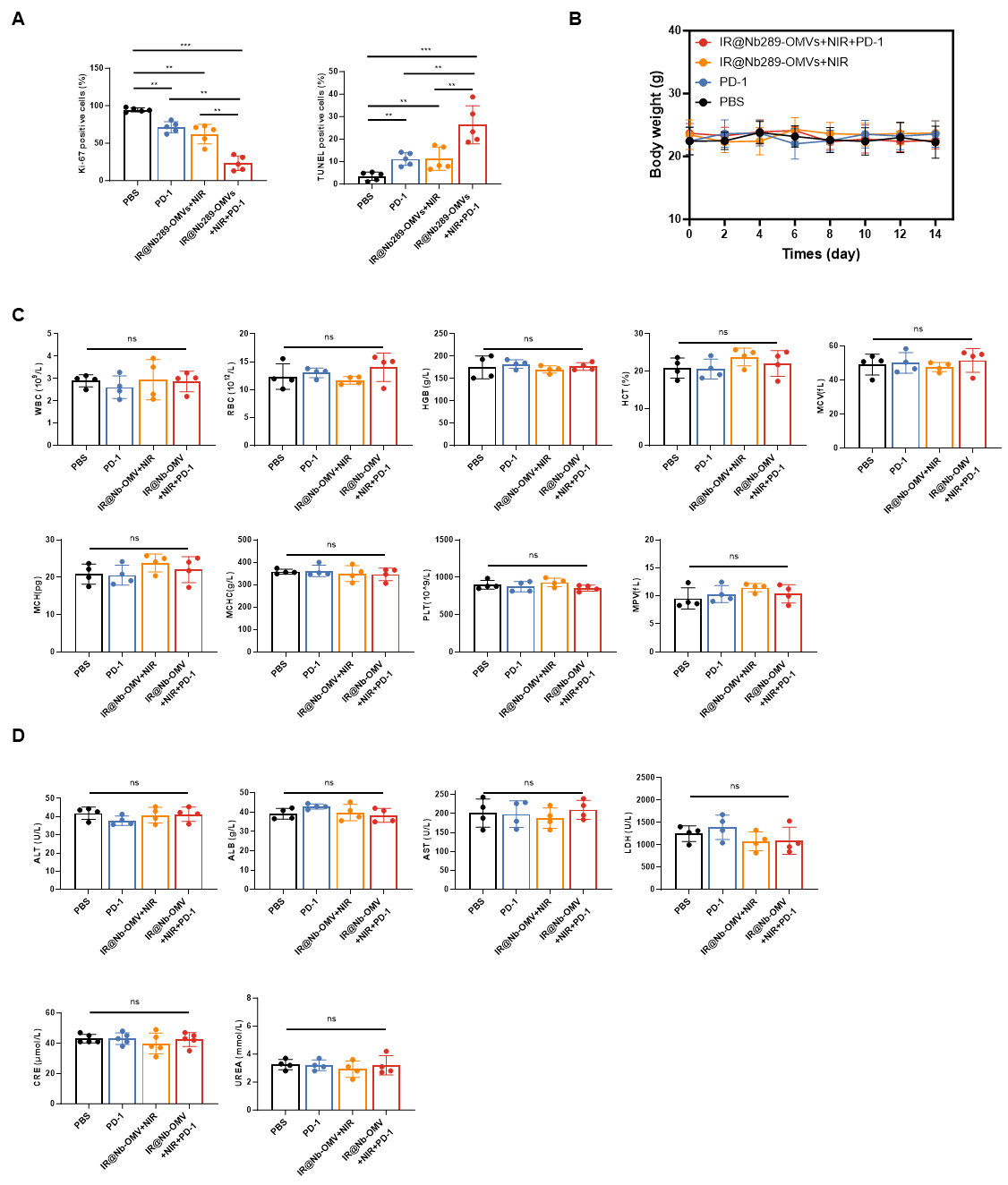
**

**Figure S25. The analysis for complete blood count and blood biochemistry from tumor bearing mice in the study of IR700@Nb289-OMVs plus NIR combined with the PD-1 blockade.**

**A,** Quantification of Ki67 positive cells and TUNEL positive cells in tumors. **B**, Body weight of mice received various treatments (*n* = 5). **C,** Parameter analysis for serum blood routine index from tumor bearing mice in the combination study with PD-1 antibodies. **D,** Blood biochemistry test from tumor bearing mice in the combination study with PD-1 antibody (*n* = 5). The results are presented as mean ± SD. Statistical significance was calculated using one-way ANOVA with Tukey’s post-test. ns indicates no significant difference. **P*<0.05, ***P*<0.01, and ****P*<0.001. WBC, White blood cells; RBC, Red blood cells; HGB, Hemoglobin; HCT, Hematocrit; MCV, Mean corpuscular volume; MCH, Mean corpuscular hemoglobin; MCHC, Mean corpuscular hemoglobin concentration; PLT, Platelets; MPV, Mean platelet volume; ALT, Alanine aminotransferase; ALB. Albumin; AST, aspartate aminotransferase; LDH, Lactate Dehydrogenase; CRE, Creatinine.

**
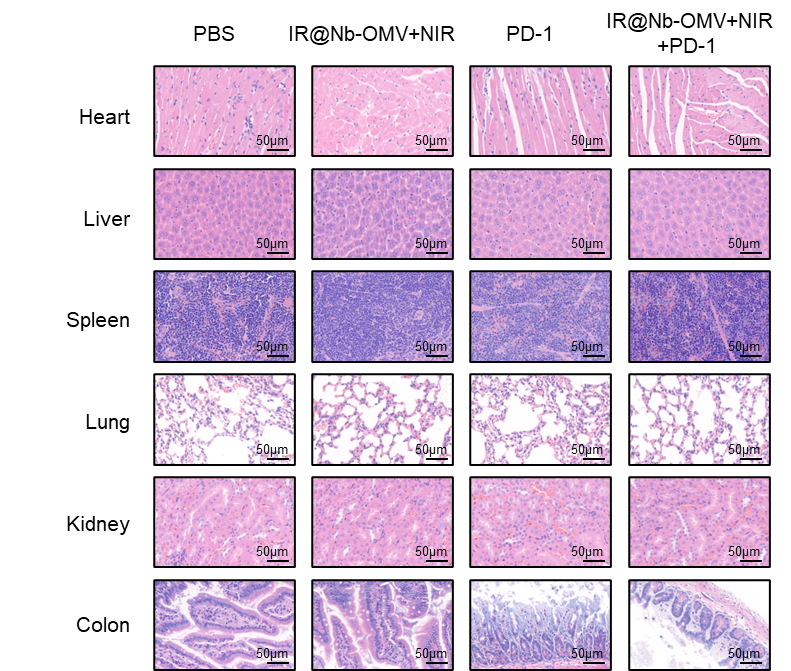
**

**Figure S26. Pathological analysis of major organs harvested** **from tumor bearing mice in the study of IR700@Nb289-OMVs plus NIR combined with the PD-1 blockade.**

H&E staining for major organs (heart, liver, spleen, lung, kidney and colon) from mice in each group for combination treatment study with PD-1 blockade (*n* = 5). Scale bars, 50 μm.

**
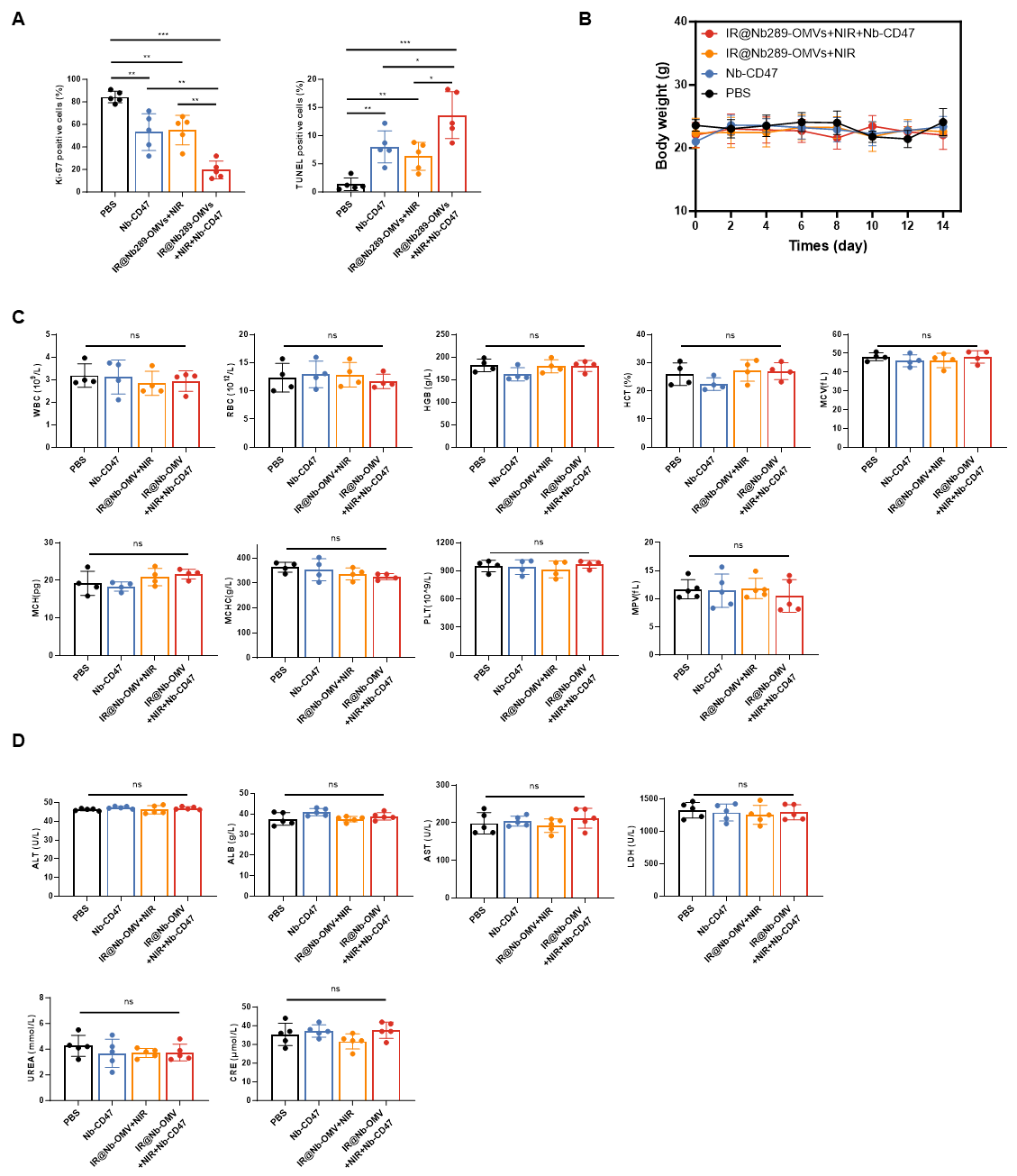
**

**Figure S27. The analysis for complete blood count and blood biochemistry from tumor bearing mice in the study of IR700@Nb289-OMVs plus NIR combined with the CD47 blockade.**

**A,** Quantification of Ki67 positive cells and TUNEL positive cells in tumors. **B,** Body weight of mice received various treatments (*n* = 5). **C,** Parameter analysis for serum blood routine index from tumor bearing mice in the combination study with CD47 nanobodies. **D,** Blood biochemistry test from tumor bearing mice in the combination study with CD47 nanobody (*n* = 5). The results are presented as mean ± SD. Statistical significance was calculated using one-way ANOVA with Tukey’s post-test. ns indicates no significant difference. **P*<0.05, ***P*<0.01, and ****P*<0.001. WBC, White blood cells; RBC, Red blood cells; HGB, Hemoglobin; HCT, Hematocrit; MCV, Mean corpuscular volume; MCH, Mean corpuscular hemoglobin; MCHC, Mean corpuscular hemoglobin concentration; PLT, Platelets; MPV, Mean platelet volume; ALT, Alanine aminotransferase; ALB. Albumin; AST, aspartate aminotransferase; LDH, Lactate Dehydrogenase; CRE, Creatinine.

**
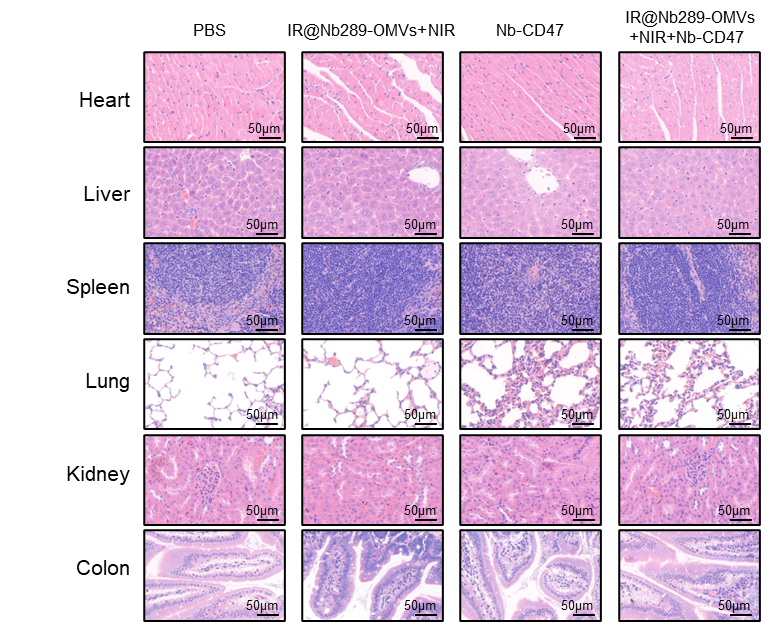
**

**Figure S28. Pathological analysis of major organs harvested from tumor bearing mice in the study of IR700@Nb289-OMVs plus NIR combined with the CD47 blockade.**

H&E staining for major organs (heart, liver, spleen, lung, kidney and colon) from mice in each group for combination treatment study with CD47 blockade (*n* = 5). Scale bars, 50 μm.

**
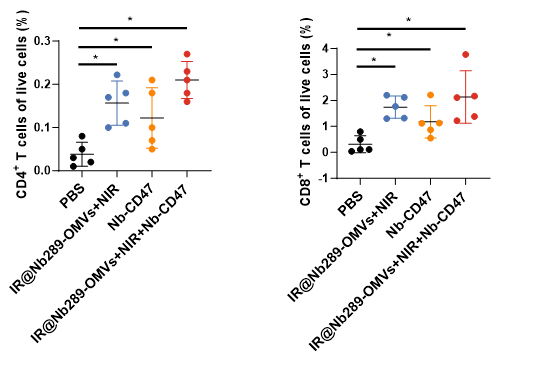
**

**Figure S29. The analysis for T cells from tumor bearing mice in the study of IR700@Nb289-OMVs plus NIR combined with the CD47 blockade.**

Quantification for CD4^+^ T cell and CD8^+^ T cells from tumor bearing mice (n = 5). The data are presented as mean ± SD. Statistical significance was calculated using one-way ANOVA with Tukey’s post-test. **P*<0.05.

**
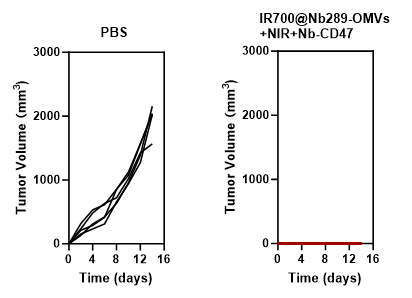
**

**Figure S30. Tumor growth analysis for rechallenging study.**

Individual tumor growth curves for tumor-cured mice and age-matched control mice receiving Colon26-Luc cell challenge (*n* = 5).

**
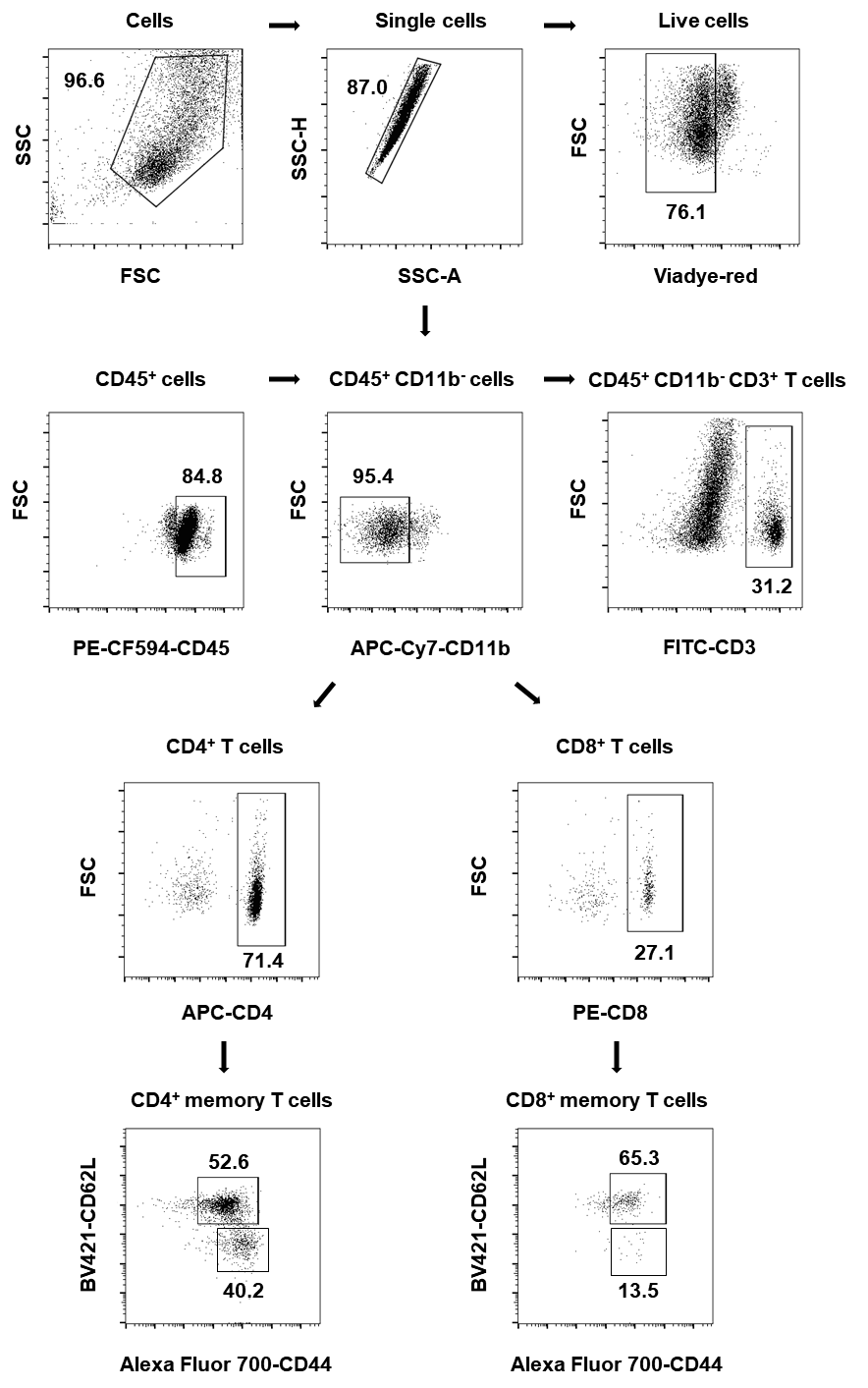
**

**Figure S31. The gating strategy for immune memory T cells in splenocytes.**

Effector memory T cells (CD62L^-^CD44^+^) and central memory T cells (CD62L^+^CD44^+^) were gated from CD4^+^ T cells (CD45^+^CD11b^-^CD3^+^CD4^+^) and CD8^+^ T cells (CD45^+^CD11b^-^CD3^+^CD8^+^).

**Supplementary materials and methods**

**Experimental Reagents**

DMEM medium, RPMI1640 medium, fetal bovine serum (FBS), penicillin-streptomycin (Pen-Strep), and Detoxi-Gel™ Endotoxin Removing Columns were obtained from Thermo Fisher Scientific (Waltham, MA, USA). Annexin V-FITC/PI apoptosis kit was obtained from MultiSciences (Hangzhou, China). 4% Paraformaldehyde (PFA) was purchased from Biyuntian Company (Shanghai, China). PKH67 Green Fluorescent Cell Linker Mini Kit, TUNEL staining kit, and BCA protein assay kit were obtained from Sigma-Aldrich (St. Louis, MO, USA). Ultracentrifuge tubes were purchased from Beckman (Fullerton, California, USA). CCK-8 was purchased from Yeasen (Shanghai, China). 2-(4-Amidinophenyl)-6-indolecarbamidine dihydrochloride (DAPI) was obtained from Beyotime (Shanghai, China).

**Loading of OMVs with IR700**

OMVs and IR700 dye were thoroughly mixed and transferred to a 4 mm electroporation cuvette. Electrotransformation was performed using a Bio-Rad electroporation instrument with parameters set at 400 V and a pulse duration of 1 ms. To remove unencapsulated IR700, the suspension was subjected to ultracentrifugation at 120,000 g for 90 minutes. The concentration of unbound IR700 was quantified using High-Performance Liquid Chromatography (HPLC). Encapsulation efficiency was calculated by comparing this measurement to the initial IR700 concentration. The encapsulation rate was determined using the following formula: (Initial molecular weight - Free molecular weight) / Initial molecular weight × 100%.

***In vivo* NIR-I fluorescence imaging**

Colon26 tumor-bearing mice with tumor volumes of approximately 500 mm³ were randomly allocated into four groups (n = 3 per group). Each group received an intravenous injection of either PBS (200 µL), free IR700 (100 µg), IR700@ConNb-OMVs (IR700: 100 µg; ConNb-OMVs: 1×10^11^ particles), or IR700@Nb289-OMVs (IR700: 100 µg; Nb289-OMVs: 1×10^11^ particles). Mice were imaged at 4, 8, 12, and 24 hours post-injection using an *in vivo* NIR-I fluorescence imaging system (IVIS, PerkinElmer, USA). At 24 hours post-injection, mice were euthanized, and tumors along with major organs (heart, liver, spleen, lung, kidney, and colon) were excised for ex vivo imaging and nanobody staining.

***In vivo* NIR-II fluorescence imaging**

Subcutaneous Colon26 tumor-bearing mice (tumor volume ~500 mm³) were randomly assigned to three groups (n = 3 per group). Mice were administered via tail vein injection with either 200 µL of PBS, 200 µL of ICG@ConNb-OMVs (ICG: 100 μg/mL; ConNb-OMVs: 1 × 10^11^ particles), or 200 µL of ICG@Nb289-OMVs (ICG: 100 μg/mL; Nb289-OMVs: 1 × 10^11^ particles). Near-infrared-II (NIR-II) fluorescence imaging was performed at 4, 8, 12, and 24 hours post-injection using an Artemis Intelligent Imaging System (Artemis Intelligent Imaging Inc., Shanghai, China). At 24 hours post-injection, the mice were euthanized, and major organs—including the tumor, heart, liver, spleen, lungs, kidneys, stomach, colon, and rectum—were excised for ex vivo NIR-II fluorescence imaging.

**Mouse Xenograft Models and Treatments**

The treatment options are summarized in **Table S3**.

To evaluate the therapeutic efficacy of IR@Nb289-OMVs, Colon26 tumor-bearing mice were randomly assigned to seven treatment groups (n = 5 per group): (1) PBS (200 µL), (2) NIR (50 J/cm²), (3) IR700 (100 μg), (4) IR700 (100 μg, equal dose with OMV-encapsulated IR700) + NIR (50 J/cm²), (5) Nb289-OMVs (1×10¹^1^ particles/injection), (6) IR700@Nb289-OMVs (1×10¹^1^ particles/injection), and (7) IR700@Nb289-OMVs (1×10¹^1^ particles/injection) + NIR (50 J/cm²). Treatments were administered three times according to the established schedule.

To assess the efficacy of IR700@Nb289-OMVs in multiple tumor models, Panc02 tumor-bearing mice were randomly divided into four groups (n = 5 per group) and treated with: (1) PBS (200 µL), (2) IR700 (100 μg, equal dose with OMV-encapsulated IR700) + NIR (50 J/cm²), (3) Nb289-OMVs (1×10¹^1^ particles/injection), or (4) IR700@Nb289-OMVs (1×10¹^1^ particles/injection) + NIR (50 J/cm²). Treatments were administered three times according to the schedule.

For combination immunotherapy, Colon26 tumor-bearing mice were randomly assigned to four groups (n = 5 per group): (1) PBS (200 µL), (2) PD-1 antibodies (12.5 mg/kg) or CD47 nanobodies (10 mg/kg), (3) IR@Nb289-OMVs (1×10¹^1^ particles/injection) + NIR (50 J/cm²), and (4) IR@Nb289-OMVs (1×10¹^1^ particles/injection) + NIR (50 J/cm²) + PD-1 antibodies (12.5 mg/kg) or CD47 nanobodies (10 mg/kg). Mice received one cycle of NIR treatment followed by two intraperitoneal injections of PD-1 antibodies/ CD47 nanobodies according to the therapeutic schedule.

For the treatment of colorectal cancer metastasis models, mice with Colon26 lung or abdominal metastases were randomly divided into four groups (n = 5-7 per group) and treated with: (1) PBS (200 µL), (2) IR@Nb289-OMVs (1×10^11^ particles/injection) + NIR (50 J/cm²), (3) Nb-CD47 (10 mg/kg), or (4) IR@Nb289-OMVs (1×10^11^ particles/injection) + NIR (50 J/cm²) + CD47 nanobodies (10 mg/kg). Mice received one cycle of NIR treatment followed by two intraperitoneal injections of CD47 nanobodies according to the therapeutic schedule.

To investigate the effects of macrophage depletion or STING pathway inhibition on tumor growth, Colon26 tumor-bearing mice in the pilot study were randomly assigned to five groups (n = 5 per group) and treated with: (1) PBS (200 µL), (2) H151 (10 mg/kg, intraperitoneal injection for 7 days), (3) clodronate liposomes (200 µL, 5 mg/mL, peritumoral injection for 7 days), (4) MSA2 (200 µL, 50 mg/kg, single subcutaneous injection), or (5) MSA2 (200 µL, 50 mg/kg, single subcutaneous injection) + H151 (10 mg/kg, intraperitoneal injection for 7 days).

To examine the impact of macrophage depletion or STING pathway inhibition on OMV-mediated photoimmunotherapy, Colon26 tumor-bearing mice were randomly divided into four groups (n = 5 per group) and treated with: (1) PBS (200 µL), (2) IR700@Nb289-OMVs (1×10^11^ particles/injection) + NIR (50 J/cm²), (3) IR700@Nb289-OMVs (1×10^11^ particles/injection) + NIR (50 J/cm²) + clodronate liposomes (200 µL, 5 mg/mL, peritumoral injection for 7 days), or (4) IR700@Nb289-OMVs (1×10^11^ particles/injection) + NIR (50 J/cm²) + H151 (10 mg/kg, intraperitoneal injection for 7 days).

**Peritoneal macrophage extraction**

A 4% thioglycolate solution was prepared and deionized. 6-8-week-old BALB/c mice were injected intraperitoneally with 1 mL of the prepared 4% thioglycolate solution. After 48 hours, mice were sacrificed, and the peritoneal cavity was lavaged with 5 mL of DMEM medium. The collected media were centrifuged at 400 × g for 5 min to pellet the cells. The cells were resuspended in DMEM containing 10% fetal bovine serum. The cells were then counted, adjusted to a concentration of 3 million cells/mL, and added to culture dishes at 37°C in an incubator with 5% CO2. After 48h, macrophages were confirmed by flow cytometry using F4/80 and CD11b antibodies and were employed for subsequent experiments.

**Protein purification**

For the expression and purification of target proteins, the recombinant plasmids pET-14B-CDH17-domains 1-3 (human and mouse) were transformed into BL21(DE3) cells. Bacterial clones were incubated at 37°C and 225 rpm until reaching an OD600 value of 0.6. The cultures were induced with 0.2 mM IPTG at 16°C and 225 rpm overnight. The cultures were then pelleted by centrifugation at 8000 g for 15 min at 4°C. Cell pellets were dissolved in lysis buffer (300 mM NaCl, 50 mM NaH_2_PO_4_, 10 mM imidazole, pH 8.0, 1 mM PMSF) and subjected to high-pressure homogenization at low temperature for 3 cycles. The lysate was centrifuged for 45 min at 12,000 × g, and the supernatants were loaded onto a gravity column containing 1 mL Ni-NTA agarose resin (Qiagen, Germany). The protein-bound resin was washed with 50 mL Wash Buffer I (300 mM NaCl, 50 mM NaH_2_PO_4_, 20 mM imidazole, pH 8.0, 1 mM PMSF) and 50 mL Wash Buffer II (300 mM NaCl, 50 mM NaH_2_PO_4_, 40 mM imidazole, pH 8.0, 1 mM PMSF), then eluted with 25 mL Elution Buffer (300 mM NaCl, 50 mM NaH2PO4, 250 mM imidazole, pH 8.0, 1 mM PMSF). Finally, the eluate was fractionated by Superdex-150 gel chromatography using an AKTA Pure System (GE Healthcare Life Sciences, USA) in 1× PBS. The purified proteins were identified by SDS-PAGE, then quickly frozen in liquid nitrogen and stored at -80°C until use. Nanobodies were purified using Ni-NTA agarose resin following the same procedures. The purified nanobodies were analyzed and identified by western blot using 6×His tag, HA tag, and anti-VHH antibodies.

**Flow analysis of immune cell infiltration in tumors**

Immune cell infiltration in tumor tissues from tumor-bearing mouse models was analyzed using a spectral flow cytometer (Cytek® Northern Lights™, Shanghai, China). Single-cell suspensions from collected tumor tissues were prepared by enzymatic digestion (RWD Life Science Co., Ltd, Shenzhen, China). Following red blood cell lysis of the cell suspension with ACK buffer, live cells were counted using Trypan Blue staining. Approximately 4×10^6^ cells per sample were resuspended in 100 μL FACS buffer. CD16/32 antibody was added and incubated at 4°C for 30 min to block Fc sites on the cell surface. Fluorescent antibodies were added to the samples according to immune cell types and incubated for 30 min on ice. Macrophages were detected using the following surface markers: CD45, F4/80, CD11b, CD80, and CD206. Dendritic cells were detected using CD45 and CD11c. T cells were detected using CD45, CD3, CD4, and CD8. Detection and analysis of single-stained tubes and sample tubes were performed using a spectral flow analyzer (Cytek, USA).

**Detection of Memory T Cells in Spleen by Flow Cytometry**

Spleen tissue was obtained by euthanizing mice and removing connective tissue from the spleen. The spleen tissue was cut into small pieces and dissociated into individual cells as much as possible, mixed with FACS buffer (PBS + 1% BSA), and filtered using a 70 μm filter membrane. The cell suspension was centrifuged at 1000 g at 4°C for 5 min, and the supernatant was discarded. ACK buffer was then added to lyse the red blood cells, followed by centrifugation at 500 g for 10 min. After discarding the supernatant, FACS Buffer was added to the cell pellet for resuspension. CD16/32 antibody was added for Fc receptor blocking, and finally, specific antibodies for memory T cells were added for cell labelling (CD45, CD3, CD4, CD8, CD62L and CD44).

**qRT-PCR**

Total RNA was extracted from tissues or cells using Invitrogen TRIzol reagent (Thermo Fisher Scientific, Waltham, MA, USA). The purity and integrity of total RNA were assessed by detect the bands of the 28S and 18S bands by agarose gel electrophoresis. Approximately 2 μg of total RNA was reverse transcribed to obtain complementary DNA. Quantitative real-time PCR (qPCR) was performed using SYBR Green RT-PCR Master Mix (BioRad, Hercules, CA, USA) on a CFX 96 RT-PCR system with specific primers (Table 1). The RT-PCR conditions were as follows: 95°C for 10 min, followed by 40 cycles of 95°C for 15 s and 60°C for 1 min. The levels of target genes were normalized using the 2^−ΔΔCt^ method with β-actin as the reference gene.

**Enzyme-Linked Immunosorbent Assay (ELISA)**

CDH17 antigen (10 μg/mL) was coated on ELISA plates overnight at 4°C. Non-specific binding sites were blocked with 3% BSA. Nanobody or OMV solutions were serially diluted in 0.1% PBST and then added to the ELISA plate. After incubation at room temperature (RT) for 1 h, the plate was washed with 0.1% PBST three times. The plate was then incubated with the detection antibody solution (anti-HA HRP antibody) in blocking buffer (3% BSA/0.1% PBST) for 1 h at RT. TMB substrate solution was added to each well, and the plate was incubated for 3-5 min at RT. Once the blue color developed, the reaction was stopped by adding 50 μL of 1 M H_2_SO_4_. Absorbance was measured at 450 nm using a spectrophotometer.

**In-cell ELISA**

To analyze the binding activity of nanobodies with gastric, colon, and pancreatic cancer cell lines, in-cell ELISA was performed as follows: Cancer cell lines were cultured overnight in 96-well plates at a density of 5×10^4^ cells/well. Cells were fixed with 4% paraformaldehyde for 5 min and then incubated with 4% donkey serum solution at RT for 1 h. Nanobodies were diluted from 4000 nM to 62.5 nM with 1× PBS containing 0.1% Tween 20 (PBST) and incubated for 1 h at RT. The plates were then washed three times with PBST, and a rabbit anti-HA antibody (Creative Biomart, USA) was added and incubated at RT for 1 h. Next, the plates were washed and incubated with an Alexa Fluor 488-linked donkey anti-rabbit IgG antibody (Invitrogen, USA) for 1 h at RT. The plates were washed three times with PBST, and fluorescence intensity was measured using a Sapphire Capture System (Sapphire, USA).

**CCK-8 assay and colony formation assay**

CCK-8 assays were performed using a commercially available kit (HY-K0301, Shanghai, China). A colony formation assay was conducted to quantify the colony-forming capacity of cells. Briefly, 1000 cells were seeded and cultured for 2 weeks. The cells were then fixed with 4% paraformaldehyde for 0.5 h and stained with 0.5% crystal violet for 1 h. Photographs were obtained, and the stained colonies were counted.

**Cellular uptake of OMVs**

PKH67 dye (4 μL) was added to an appropriate amount of OMVs (1×10^11^ particles/mL) and incubated at room temperature for 30 min in the dark. Subsequently, the solution was centrifuged at 100,000 g at 4°C for 60 min to collect the precipitate. Tumor cells were seeded into 96-well plates at a density of 2×10^4^cells/well and cultured for 24 h. The labeled OMVs were then added to the tumor cells and incubated for 2 h or 8 h. Cells were washed 3 times with PBS and fixed with 4% PFA solution for 30 min. Finally, the nuclei were stained with DAPI solution, and the cells were imaged using a confocal microscope.

**Scratch wound-healing motility assay and transwell invasion assay**

For the scratch wound-healing motility assay, Colon26 cells were seeded in 6-well plates (1×10^6^ cells/well) and cultured in DMEM medium without FBS supplementation for 24 h. An artificial wound was created using a 10 μL sterile pipette tip. Cells were then subjected with various treatments. Images of the scratches were captured at 0 h and 24 h. The ratio of the scratch area to the total image area was calculated to determine the cell migration rate.

Transwell invasion assays were performed using Matrigel-coated chambers (6.5 mm diameter, 8 μm pore size; Corning Life Sciences, NY, USA) in 24-well plates. In the upper chamber, 1 × 10^5^ cells were seeded in serum-free medium, while medium containing 10% FBS was placed in the lower chamber. After 48 h of incubation with various treatments, cells remaining in the upper chamber were removed. Invaded cells were fixed with 4% PFA and stained with 0.1% crystal violet solution.

**Cell apoptosis analysis**

Cells were grown to 80% confluence in 6-well plates and then incubated with various drugs for 24 h. Apoptosis was detected using an Annexin V-FITC/PI Apoptosis Kit (Lianke, Hangzhou, Zhejiang, China). After staining, the cells were analyzed using a flow cytometer (CytoFLEX; Beckman Coulter, Inc., Fullerton, CA, USA). Data were analyzed using FlowJo software Version 10.0.7 (FlowJo, LLC, Oregon, USA).

**Terminal deoxynucleotidyltransferase-mediated dUTP-biotin nick end labeling (TUNEL) staining**

Tissue sections were dewaxed and treated with 20 μg/mL DNase-free protease K (Beyotime Biotechnology, Hangzhou, Zhejiang, China) at 37°C for 30 min. Sections were washed with PBS three times. Then, 50 μL TUNEL reaction mixture (Beyotime Biotechnology) was added to the samples and incubated for 60 min at 37°C, protected from light. The slides were dried and sealed with anti-fluorescence quenching mounting medium. Tissue sections were observed under a fluorescence confocal microscope (Leica Biosystems, Wetzlar, Hessen, Germany).

**Immunofluorescence and immunohistochemical staining**

For immunofluorescence, sections were fixed using acetone/methanol (4:1) at -20 degree for 10 min. Non-specific binding was blocked with 4% donkey serum in PBS for 30 min at RT. Sections were then incubated with primary antibodies overnight at 4°C. The following day, sections were stained with secondary antibodies conjugated fluorophores in 0.5% donkey serum/PBS for 1 h at RT in the dark. Sections were mounted with mounting medium containing 4′,6-diamidino-2-phenylindole (DAPI, Sigma-Aldrich, St Louis, MO, USA).

For immunohistochemistry, tissues were fixed, embedded, and sectioned. Paraffin-embedded tissues were deparaffinized and rehydrated using xylene and ethanol. Antigen retrieval was performed by steaming slides for 15 min in Tris-ethylenediaminetetraacetic acid (EDTA) buffer (pH 9.0). Slides were then incubated in 0.3% H_2_O_2_-methanol (Aladdin, Shanghai, China) for 10 min. Non-specific binding was blocked with 4% donkey serum in Tris-buffered saline with 0.1% Tween 20 (TBST) for 1 h. Slides were incubated with primary antibodies overnight at 4°C, followed by incubation with biotinylated secondary antibodies for 60 min. Slides were then incubated for 30 min with VECTASTAIN Elite ABC Reagent (Vectorlabs, Burlingame, CA, USA) and reacted with 3,3′-diaminobenzidine (DAB, Vectorlabs) peroxidase substrate solution until the desired stain intensity developed. Dehydrated slides (70% ethanol-xylene substitute) were air-dried and mounted with neutral mounting medium (Solaibao, Beijing, China).

**Western blotting analysis**

Total proteins were extracted using Radioimmunoprecipitation assay (RIPA) buffer (Beyotime Biotechnology, Shanghai, China). Protein concentrations in supernatants were quantified using Bicinchoninic Acid (BCA) protein assays (Beyotime Biotechnology). Proteins were separated by sodium dodecyl sulfate-polyacrylamide gel electrophoresis (SDS-PAGE) (10%, 12.5%, and 15%) and transferred onto polyvinylidene fluoride (PVDF) membranes (Millipore, Billerica, MA, USA). Membranes were blocked for 1-2 h with 5% nonfat dry milk (Biosharp, Hefei, Anhui, China) and incubated with various primary antibodies overnight at 4°C. Subsequently, the membranes were incubated with HRP-conjugated secondary antibodies. Protein bands were detected using enhanced chemiluminescence (ECL) detection reagent (GE Healthcare, Amersham, Buckinghamshire, UK).

**Fluorescent Western Blotting and Antibodies for copy number determination**

Nanobodies with known concentrations and engineered OMVs were separated by 15% SDS-PAGE and transferred onto PVDF membranes (Millipore, USA). Membranes were blocked with 5% nonfat dry milk for 1 h and incubated with HA-tag antibody (Abmart, China, #M20003) overnight at 4°C. Subsequently, membranes were incubated with IRDye 680 antibody (LI-COR, #D01202-12). Membranes were washed in TBST three times for 5 min each, with the final wash performed in TBS. Protein bands were detected using a fully automated electrophoretic fluorescence immunoassay analyzer (Azure Biosystems, CA, USA). The fluorescence intensity of protein bands was calculated and analyzed using ImageJ software to establish a standard curve, which was further used to determine the copy numbers of nanobody displayed on each OMV.

**RNA-sequencing (RNA-seq)**

Total RNA was extracted from PBS-treated and IR700@Nb-OMVs plus NIR-treated Colon26 tumor samples. RNA concentration and purity were assessed using a NanoDrop 2000 spectrophotometer (NanoDrop Technologies, Wilmington, DE, USA). RNA integrity was evaluated using agarose gel electrophoresis, and the RNA integrity number (RIN) was determined using an Agilent 2100 Bioanalyzer (Agilent Technologies, Palo Alto, CA, USA).

For transcriptome analysis, mRNA was isolated from total RNA via A-T base pairing of Oligo (dT) magnetic beads with poly (A) tails. Fragmentation buffer was added to randomly fragment the mRNA, and fragments of approximately 300 bp were isolated using magnetic beads. Libraries were sequenced using the Illumina NovaSeq 6000 platform (Illumina, San Diego, CA, USA). Differentially expressed genes (DEGs) were defined as genes with a P value < 0.05 and an absolute log2 (fold change) > 2.

**Transmission Electron Microscopy (TEM)**

The morphology of OMVs was observed by TEM (JEOL JEM-1400 Plus, JEOL, Tokyo, Japan). Briefly, approximately 30 μL of sample was pipetted onto a carbon-coated copper grid (Xinxing Braim, Beijing, China). After 5 minutes, excess sample was removed, and a drop of 4% uranyl acetate stain was added to the grid. After air-drying, the samples were examined by TEM.

**Immunogold Labeling of Whole-Mount EVs**

EVs were resuspended in 50-100 µL of 2% paraformaldehyde (PFA). A 5 µL aliquot of the EV suspension was deposited onto a Formvar carbon-coated electron microscope grid and allowed to adsorb for 20 minutes in a dry environment. The grid was subsequently transferred to phosphate-buffered saline (PBS) and washed twice for 3 minutes each. Following this, the grid was transferred to PBS containing 50 mM glycine for 3 minutes, with this step repeated three times. The grid was then incubated with 5% bovine serum albumin (BSA) blocking buffer for 10 minutes. The HA magnetic beads, diluted 1:20 in blocking buffer, were applied to the grid for 30 minutes. Post-antibody labeling, the grid was incubated with 1% glutaraldehyde for 5 minutes, followed by eight sequential washes in distilled water for 2 minutes each. Use 10ul uranyl acetate to negatively stain the grid for 90s, then dry the grid. Samples were visualized using an electron microscope operated at 120 kV.

**Statistical Analyses**

Data are presented as mean ± standard deviation (SD) unless otherwise specified. Statistical analyses were conducted using Prism software (GraphPad Prism, version 10.1). To compare datasets from two distinct groups, two-tailed unpaired t-test was employed. Assessment of in vitro cell viability and tumor growth curves was performed using one-way Analysis of Variance (ANOVA) or two-way ANOVA with Tukey’s post-test. Survival rates were analyzed using the Kaplan-Meier method and Mantel–Cox test. Statistical significance was determined at P values of 0.05 or lower. The notation "ns" indicates a lack of statistical significance, with **P* < 0.05, ***P* < 0.01, and ****P* < 0.001 denoting various levels of significance.

**Table S1**, The primary antibody used in this study

| Antibody | Catalogue number | Supplier name |
| --- | --- | --- |
| Calretinin antibody | ab92341 | Abcam |
| Donkey Anti-Goat IgG H&L Biotin | ab6884 | Abcam |
| ERp57 antibody | ab10287 | Abcam |
| HA tag (C29F4) Rabbit mAb (#3724), | 10014108 | Cell Signaling Technology |
| HA tag (6E2) Mouse mAb | 2350 | Cell Signaling Technology |
| F4/80 | MCA497R | Bio-RAD |
| His-Tag | 66005-1-Ig | Cell Signaling Technology |
| HA-tag antibody | M20003 | Abmart |
| IRDye 680 antibody | D01202-12 | LI-COR |
| *cGAS antibody* | *A8335* | ABclonal |
| STING antibody | *A21051* | ABclonal |
| TBK1 antibody | *A3458* | ABclonal |
| Phospho-TBK1 antibody | *AP0847* | ABclonal |
| IRF3 antibody | *A2172* | ABclonal |
| Phospho-IRF3 antibody | *AP0623* | ABclonal |
| FITC anti-mouse CD3 Antibody | 100203 | Biolegend |
| PE anti-mouse CD45 | 147711 | Biolegend |
| APC/Cyanine7 anti-mouse CD8b.2 Antibody | 140421 | Biolegend |
| APC anti-mouse CD4 Antibody | 100411 | Biolegend |
| APC/Cyanine7 anti-mouse CD86 Antibody | 159217 | Biolegend |
| PE/Cyanine7 anti-mouse CD206 (MMR) Antibody | 141719 | Biolegend |
| PE anti-mouse CD11c Antibody | 117307 | Biolegend |
| Brilliant Violet 421™ anti-mouse CD62L Antibody | 104435 | Biolegend |
| Alexa Fluor® 700 anti-mouse CD44 Antibody | 156009 | Biolegend |
| APC/Cyanine7 anti-mouse CD11b Antibody | 101225 | Biolegend |
| PE anti-mouse CD8b.2 Antibody | 140408 | Biolegend |

**Table S2**, List of genes examined by real-time PCR and the primer sequences used

| Gene name | Forward primer | Reverse primer |
| --- | --- | --- |
| OMV-16S rDNA | CCTACGGGAGGCAGCAG | ATTACCGCGGCTGCTGGC |
| m_CCL5 | GCTGCTTTGCCTACCTCTCC | TCGAGTGACAAACACGACTGC |
| m_IFNB1 | CAGCTCCAAGAAAGGACGAAC | GGCAGTGTAACTCTTCTGCAT |
| m_CXCL10 | CCAAGTGCTGCCGTCATTTTC | GGCTCGCAGGGATGATTTCAA |
| m_TNF-α | GGTCCCCAAAGGGATGAGAAGT | TTGCTACGACGTGGGCTACA |
| m_IL6 | CCAAGAGGTGAGTGCTTCCC | CTGTTGTTCAGACTCTCTCCCT |
| m_IFNG | ATGAACGCTACACACTGCATC | CCATCCTTTTGCCAGTTCCTC |
| *m_ISG15* | GGTGTCCGTGACTAACTCCAT | TGGAAAGGGTAAGACCGTCCT |
| *m_MX1* | GACCATAGGGGTCTTGACCAA | AGACTTGCTCTTTCTGAAAAGCC |
| *m_IFIH1* | AGATCAACACCTGTGGTAACACC | CTCTAGGGCCTCCACGAACA |
| *m_IFIT1* | CTGAGATGTCACTTCACATGGAA | GTGCATCCCCAATGGGTTCT |
| *m_IFIT2* | AGTACAACGAGTAAGGAGTCACT | AGGCCAGTATGTTGCACATGG |
| *m_ISG15* | GGTGTCCGTGACTAACTCCAT | TGGAAAGGGTAAGACCGTCCT |
| *m_OASL1* | CAGGAGCTGTACGGCTTCC | CCTACCTTGAGTACCTTGAGCAC |
| *m_β-actin* | GGCTGTATTCCCCTCCATCG | CCAGTTGGTAACAATGCCATGT |

**Table S3**, Drug doses and treatment frequencies for different experimental schemes

| **Therapy regimen** | **Treatment Frequency** | **OMV dose (per injection)** | **IR700 dose (per injection)** | **CD47 dose** | **PD-1 dose** | **Figures** |
| --- | --- | --- | --- | --- | --- | --- |
| OMV | 7×OMV | 1×10^11^ particles |  |  |  | 2D |
| OMV+IR700 | 3×IR@OMV | 1×10^11^ particles | 100 μg |  |  | 3G,3Q |
| OMV+IR700+CD47 | 1×IR@OMV  2×CD47 | 1×10^11^ particles | 100 μg | 10 mg/kg |  | 6A-G |
| OMV+IR700+PD-1 | 1×IR@OMV  2×PD-1 | 1×10^11^ particles | 100 μg |  | 12.5 mg/kg | 6H-M |
